# Reaction Mechanism of Human PAICS Elucidated by Quantum Chemical Calculations

**DOI:** 10.1021/jacs.2c05072

**Published:** 2022-08-01

**Authors:** Mario Prejanò, Jana Škerlová, Pål Stenmark, Fahmi Himo

**Affiliations:** † Department of Organic Chemistry, Arrhenius Laboratory, Stockholm University, SE-10691 Stockholm, Sweden; ‡ Institute of Organic Chemistry and Biochemistry, Czech Academy of Sciences, Flemingovo nam. 2, 160 00 Prague, Czech Republic; § Department of Biochemistry and Biophysics, Stockholm University, SE-10691 Stockholm, Sweden

## Abstract

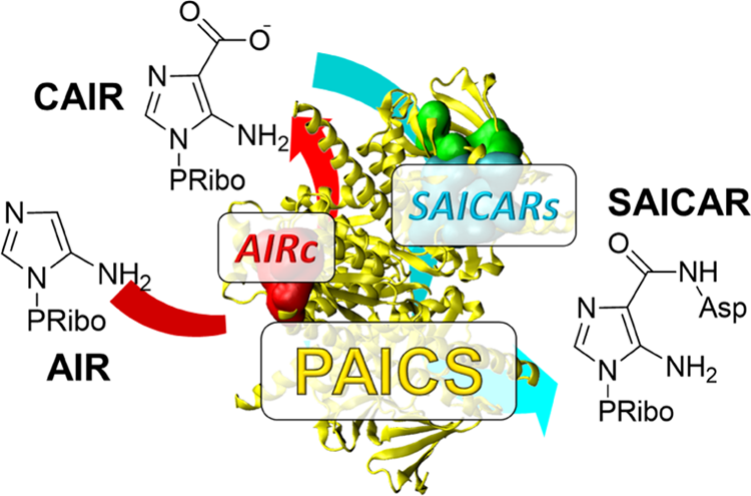

Human PAICS is a bifunctional enzyme that is involved in the *de novo* purine biosynthesis, catalyzing the conversion of aminoimidazole ribonucleotide (AIR) into *N*-succinylcarboxamide-5-aminoimidazole ribonucleotide (SAICAR). It comprises two distinct active sites, AIR carboxylase (AIRc) where the AIR is initially converted to carboxyaminoimidazole ribonucleotide (CAIR) by reaction with CO_2_ and SAICAR synthetase (SAICARs) in which CAIR then reacts with an aspartate to form SAICAR, in an ATP-dependent reaction. Human PAICS is a promising target for the treatment of various types of cancer, and it is therefore of high interest to develop a detailed understanding of its reaction mechanism. In the present work, density functional theory calculations are employed to investigate the PAICS reaction mechanism. Starting from the available crystal structures, two large models of the AIRc and SAICARs active sites are built and different mechanistic proposals for the carboxylation and phosphorylation–condensation mechanisms are examined. For the carboxylation reaction, it is demonstrated that it takes place in a two-step mechanism, involving a C–C bond formation followed by a deprotonation of the formed tetrahedral intermediate (known as isoCAIR) assisted by an active site histidine residue. For the phosphorylation–condensation reaction, it is shown that the phosphorylation of CAIR takes place before the condensation reaction with the aspartate. It is further demonstrated that the three active site magnesium ions are involved in binding the substrates and stabilizing the transition states and intermediates of the reaction. The calculated barriers are in good agreement with available experimental data.

## 1 Introduction

The *de novo* purine biosynthesis pathway comprises a series of reaction steps converting phosphoribosyl pyrophosphate into the final product, inosine monophosphate, which represents a key building block in nucleotide metabolism. The pathway is ubiquitous in all domains of life. Eukaryotic *de novo* purine biosynthesis pathway includes 10 enzymatic reactions, and in humans, reaction steps 6 and 7 are catalyzed by the bifunctional enzyme PAICS (phosphoribosylaminoimidazole carboxylase and phosphoribosylaminoimidazolesuccinocarboxamide synthetase).^1^

Cancer cells strongly depend on the *de novo* purine biosynthesis pathway, in contrast to healthy cells, which preferentially produce purines through the nucleotide salvage pathway. Therefore, all enzymes that participate in the *de novo* purine biosynthesis pathway can be considered potential targets for anticancer therapy.^2^ Indeed, PAICS is overexpressed in several types of cancer and has been shown to play an important role in cancer cell proliferation and invasion, correlating therefore with poor prognosis.^3^ Accordingly, PAICS has been explored as a potential cancer therapy target. Its knockdown resulted in reduced tumor growth in a mouse model of prostate cancer,^3g^ and shRNA-mediated PAICS depletion reduced tumor growth in mouse models of lung,^3k^ pancreas,^4^ breast,^5^ colorectal,^6^ and gastric cancer.^7^ PAICS has also been suggested as a target for treating neuroblastoma.^8^ A recent study has shown that pharmacologic inhibition of PAICS by a recently developed inhibitor had an antileukemia effect both *in vitro* and *in vivo* in a mouse xenograft model of acute myeloid leukemia, thus identifying PAICS as a chemotherapy target.^9^

Substrate/product-based inhibitors of PAICS homologues have been synthesized in the past as tools in enzymatic analysis^10^ and recent efforts have been made to develop inhibitors of prokaryotic PAICS homologues to combat antibiotic resistance.^11^

**Figure 1:**
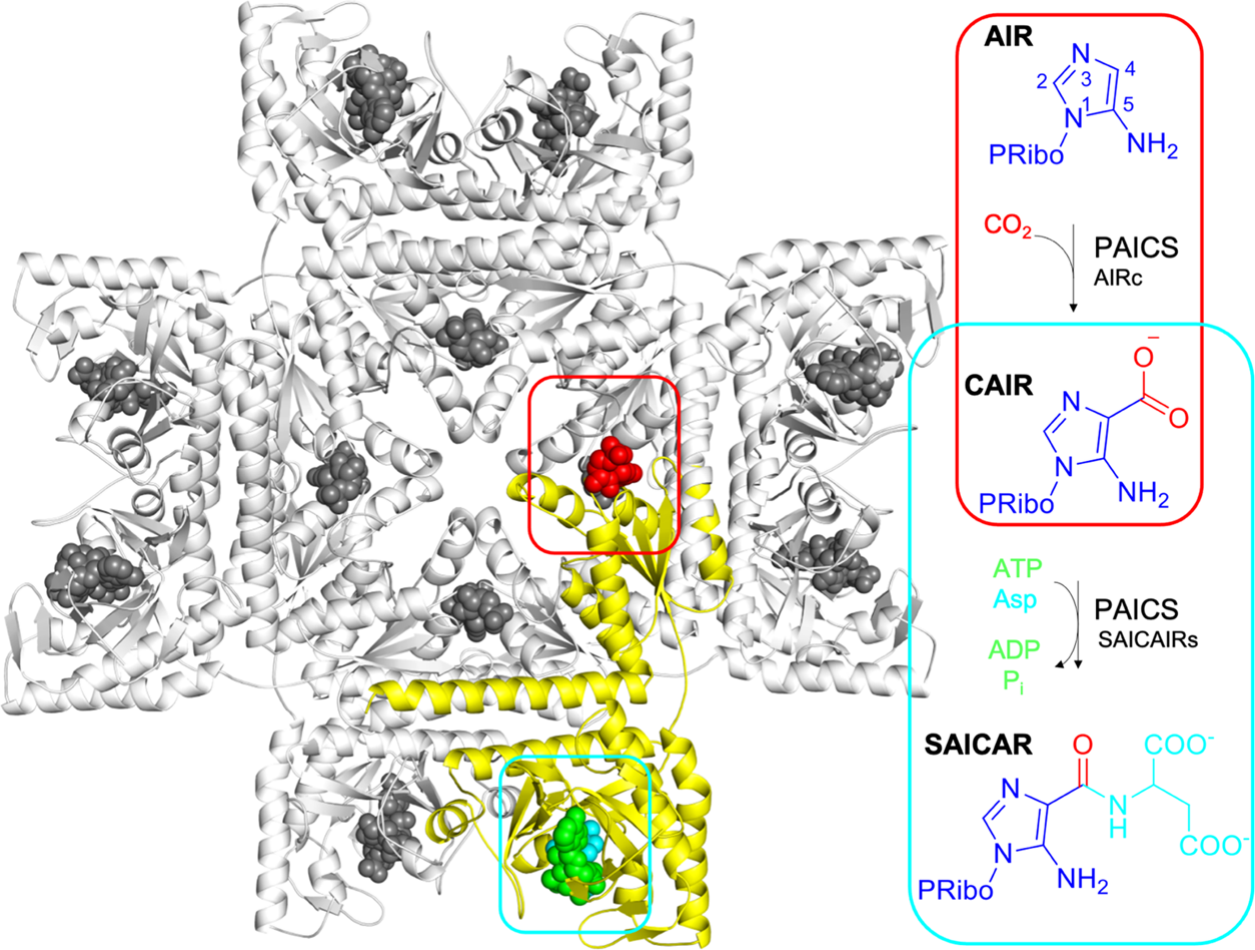
Molecular structure of human PAICS and the reactions catalyzed. The structure of the human PAICS octamer is shown in a cartoon representation, with one monomer of the enzyme highlighted in yellow. Native ligands are shown as spheres, with **CAIR**, **SAICAR**, and adenosine 5′-(β,γ-imido)triphosphate (**AMP-PNP**) colored in red, cyan, and green, respectively, in the highlighted monomer.

In animals, the bifunctional enzyme PAICS catalyzes sequentially the carboxylation of aminoimidazole ribonucleotide (**AIR**) to carboxyaminoimidazole ribonucleotide (**CAIR**) and, in the following reaction, the condensation of **CAIR** and l-aspartate (**Asp**) to *N*-succinylcarboxamide-5-aminoimidazole ribonucleotide (**SAICAR**). The latter reaction takes place in an ATP-dependent manner. The AIR carboxylase (AIRc) and SAICAR synthetase (SAICARs) activities are combined in one polypeptide chain (see Figure 1).

While the biosynthesis of **SAICAR** is universal across the tree of life, **CAIR** is synthesized in two different ways. In animals, the bifunctional PAICS contains a type II PurE domain (AIRc) that catalyzes the biosynthesis of **CAIR** in one step, as a direct carboxylation of **AIR** by **CO**_**2**_ at C^4^ (see Figure 1). This is in contrast to the catalytic strategy adopted by plants, yeast, and most prokaryotes, which synthesize **CAIR** in two steps using two enzyme activities. In the first reaction, PurK catalyzes the carboxylation of **AIR** at N^5^ with bicarbonate, in the presence of **ATP**. The product, N^5^–CAIR, is then converted to **CAIR** by an N^5^–CAIR mutase (type I PurE, a homolog of PurE-II).^12^

The structural characterization of prokaryotic PurE-I and PurE-II, prokaryotic and yeast SAICARs (PurC) enzymes, as well as bifunctional PAICS enzymes, including their complexes with natural ligands (substrates and products)^13^ has led to a number of mechanistic proposals for PAICS. More specifically, two alternative proposals have been put forward for the carboxylation of **AIR**, common to both the PurE-II reaction and the second PurE-I half-reaction,^13c,13d,13l,13o,14^ and three mechanisms have been proposed for SAICARs^13j,13s,15^ (see the Results and Discussion section below).

A detailed understanding of the catalytic mechanism of eukaryotic bifunctional PAICS and the molecular structure of intermediates and transition states is of crucial importance for emerging cancer therapies targeting human PAICS, because it can aid the rational design of novel PAICS inhibitors. For this reason, we report here a detailed computational investigation of the reaction mechanism of human PAICS, adopting the quantum chemical cluster approach, a methodology that has been successfully applied to study a vast number of enzymatic systems.^16^ We focus on the three chemical reactions involved in the conversion of **AIR** to **SAICAR**, namely, carboxylation in AIRc and phosphorylation and condensation in SAICARs. We use two large active site models, one for the AIRc and one for the SAICARs catalytic pocket. The results of the investigation are in good agreement with available experimental observations. They shed light on the source of the catalytic power of the bifunctional enzyme and help to rule out several suggestions regarding the chemical steps toward **SAICAR**. The current computational study can thus contribute to the rational design of potent and selective PAICS inhibitors as novel anticancer therapeutics.

## 2 Computational Methods

### 2.1 Active Site Models

Two active site models were designed from the recently solved crystal structures of PAICS from *Homo sapiens*, in complex with **CAIR** (PDB 6YB8) and with **SAICAR** and **AMP-PNP**, an analogue of **ATP** (PDB 6YB9).^13u^

In the AIRc active site model, the **CAIR** product from the crystal structure was manually replaced by the **AIR** and **CO_2_** substrates. The model consists of the amino acids composing the active site and interacting with the substrates: Met272, Gly273, Ser274, Thr275, Ser276, Asp277, Val328, Ala329, Gly330, Arg331, Ser332, Asn333, Gly334, Leu335, Ser301, Ala302, His303, and Lys304. One crystallographic water, bridging Ser332 and His303, was also explicitly included. The final size of the model is 269 atoms and the total charge is 0.

**Figure 2:**
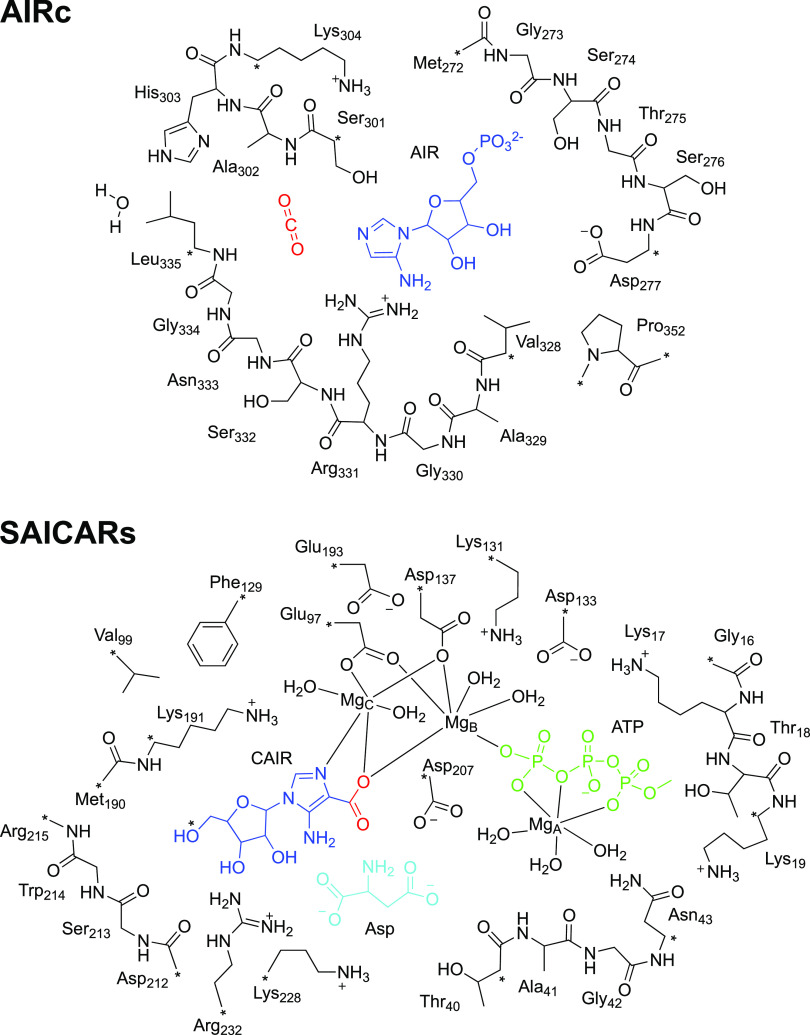
Schematic illustration of the active site models used in the current study. Carbon atoms labeled with “*” were kept fixed during the geometry optimizations.

In the SAICARs active site model, the **CAIR** and **Asp** substrates were obtained by splitting the **SAICAR** product from the crystal structure, while **AMP-PNP** was changed into **ATP**. The amino acids belonging to the **ATP** binding site (Gly16, Lys17, Thr18, and Lys19) and **SACAIR** binding site (Thr40, Ala41, Gly42, Asn43, Glu97, Val99, Lys131, Asp133, Asp137, Phe129, Met190, Lys191, Glu193, Asp207, Asp212, Ser213, Trp214, Arg215, Lys228, Arg232) were included. In addition, three hexa-coordinated Mg ions (Mg*_A_*, Mg*_B_*, and Mg*_C_*, see Figure 2) and seven water molecules coordinating to the metals were included in the model, resulting in a model size of 369 atoms and an overall charge of −1. Mg*_A_* and Mg*_C_* were present in the crystal structure, while Mg*_B_* was added manually in the model. Although Mg*_B_* is absent in the electron density map of the *H. sapiens* PAICS crystal structure, its existence is supported by enzyme kinetics experiments indicating that a higher number of Mg ions are required for catalysis than those required for the saturation of **ATP**.^13j,13s,15^ This thus suggests that the enzyme carries out the catalytic activity in the presence of three cations. In addition, the crystal structure of SAICARs from *E. coli* in complex with **ADP** and **CAIR** contains three ions, providing further evidence for the presence of Mg*_B_* in PAICS. Accordingly, the position of Mg*_B_* in the active site model was identified by the superposition of the *H. sapiens* crystal structure with the one of SAICARs:ADP:CAIR from *E. coli*, which contains all three metal ions (see Figure S1).^13j^

The amino acids, cofactors, and substrates were truncated, as shown in Figure 2. Hydrogen atoms were added manually to saturate the carbon atoms. The phosphate group of the **AIR** substrate was modeled in the dianionic state. However, during the geometry optimizations, a proton moved over spontaneously from the positively charged Lys304 to one of the oxygens. The **Asp** substrate in the phosphorylation–condensation reaction was modeled in the dianionic state, *i.e.*, with the two carboxylates in the deprotonated form (−COO^–^) and a neutral amino group (−NH_2_). This choice is based on visual inspection of the active site showing that the two carboxylate moieties can form salt bridges to several positively charged groups (Arg232, Lys228, and Lys191), while the amino group can accept a hydrogen bond from the **CAIR** substrate and in the neutral form can act as a nucleophile in the reaction (see below).

A number of carbon atoms, typically where the truncation was made (labeled with “*” in Figure 2), were kept fixed during the geometry optimizations to avoid large artificial movements as compared to the crystal structure. This procedure leads to a number of imaginary frequencies (<50*i* cm^–1^) that can be ignored since they do not affect the relative energies of the optimized structures.^16a^

### 2.2 Computational Details

All calculations were carried out using the B3LYP-D3(BJ) functional^17^ as implemented in Gaussian16 C.01.^18^ The geometry optimizations were performed employing the LANL2DZ pseudopotential^19^ for Mg and the 6–31G(d,p) basis set for other atoms. At the same level of theory, the effect of protein surrounding was calculated using single-point energy calculations with the solvation model based on density (SMD) solvation method (ε = 4),^20^ and frequency calculations were performed to obtain zero-point energy (ZPE). The entropy contribution from the inclusion of a small gas molecule was estimated to be equal to the translational entropy of the free molecule. The calculated entropy for **CO_2_** is 11.1 kcal/mol at room temperature, which is added to the energy of the formation of the enzyme–CO_2_ adduct, a procedure used previously in other studies.^21^ To obtain more accurate electronic energies, single-point energy calculations were performed using the LANL2DZ basis set for Mg and the larger 6–311+G(2d,2p) basis set for other atoms. The energies presented in the paper are the larger basis set energies including solvent and ZPE corrections.

## 3 Results and Discussion

**Scheme 1:**
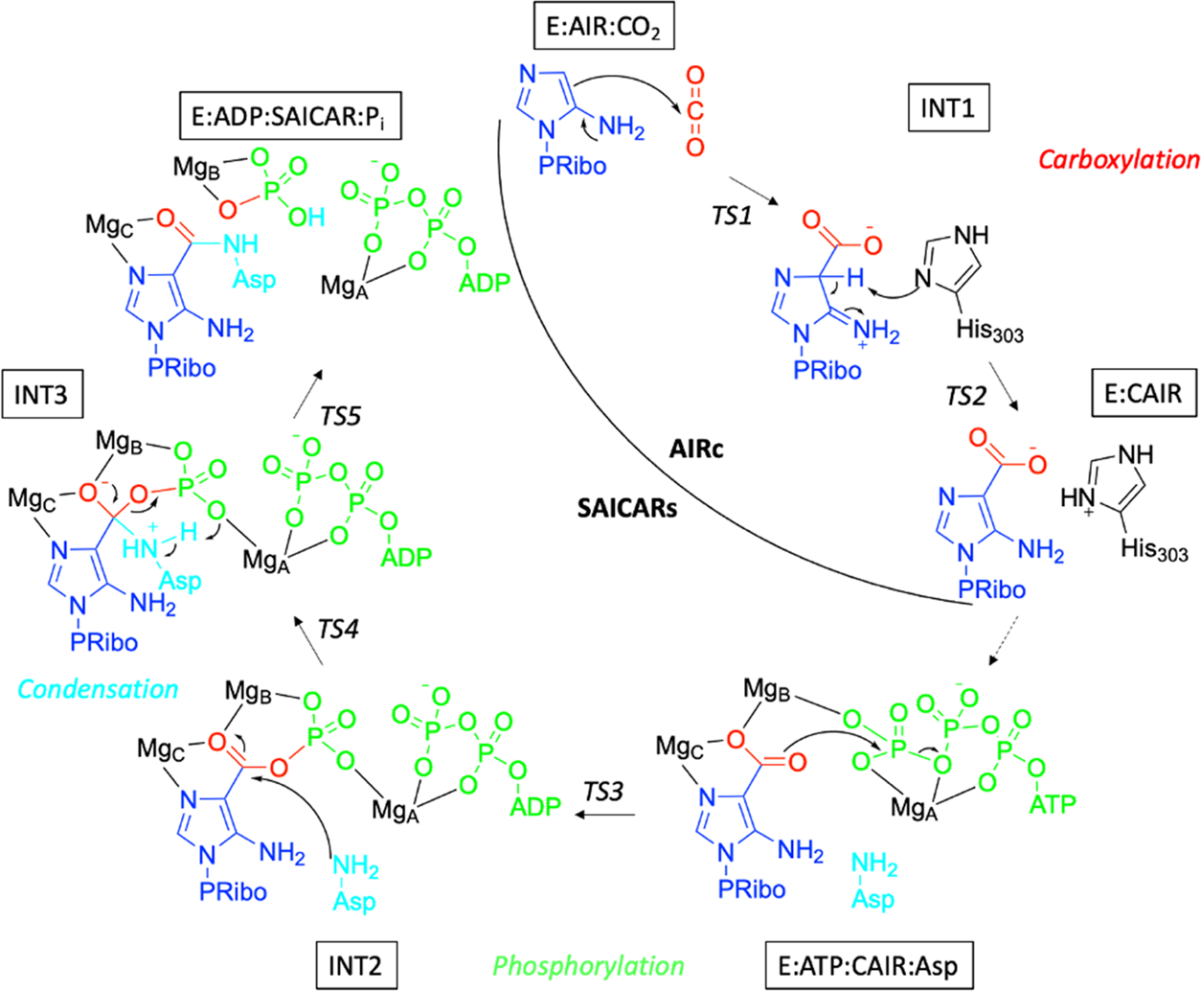
Reaction Mechanism of Human PAICS Proposed on the Basis of the Current Calculations

The complete catalytic mechanism of the human PAICS proposed here is characterized by three different reactions, taking place consecutively in the AIRc and SAICARs subunits of the enzyme (Scheme 1). The **AIR** substrate is first carboxylated in the AIRc active site by reaction with **CO_2_**, thus generating **CAIR**. Subsequently, **CAIR** is phosphorylated by **ATP** in the SAICARs active site, followed by a reaction with **Asp** to yield the **SAICAR** product. The mechanistic details of each reaction, as obtained by the current calculations, are presented in the following sections.

### 3.1 Carboxylation in the AIRc Active Site

**Figure 3:**
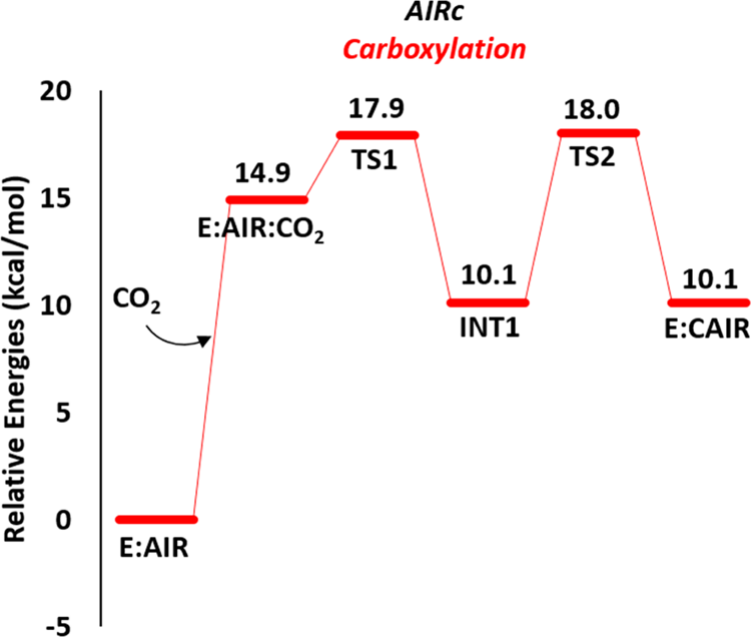
Calculated energy profile for the carboxylation reaction.

**Figure 4:**
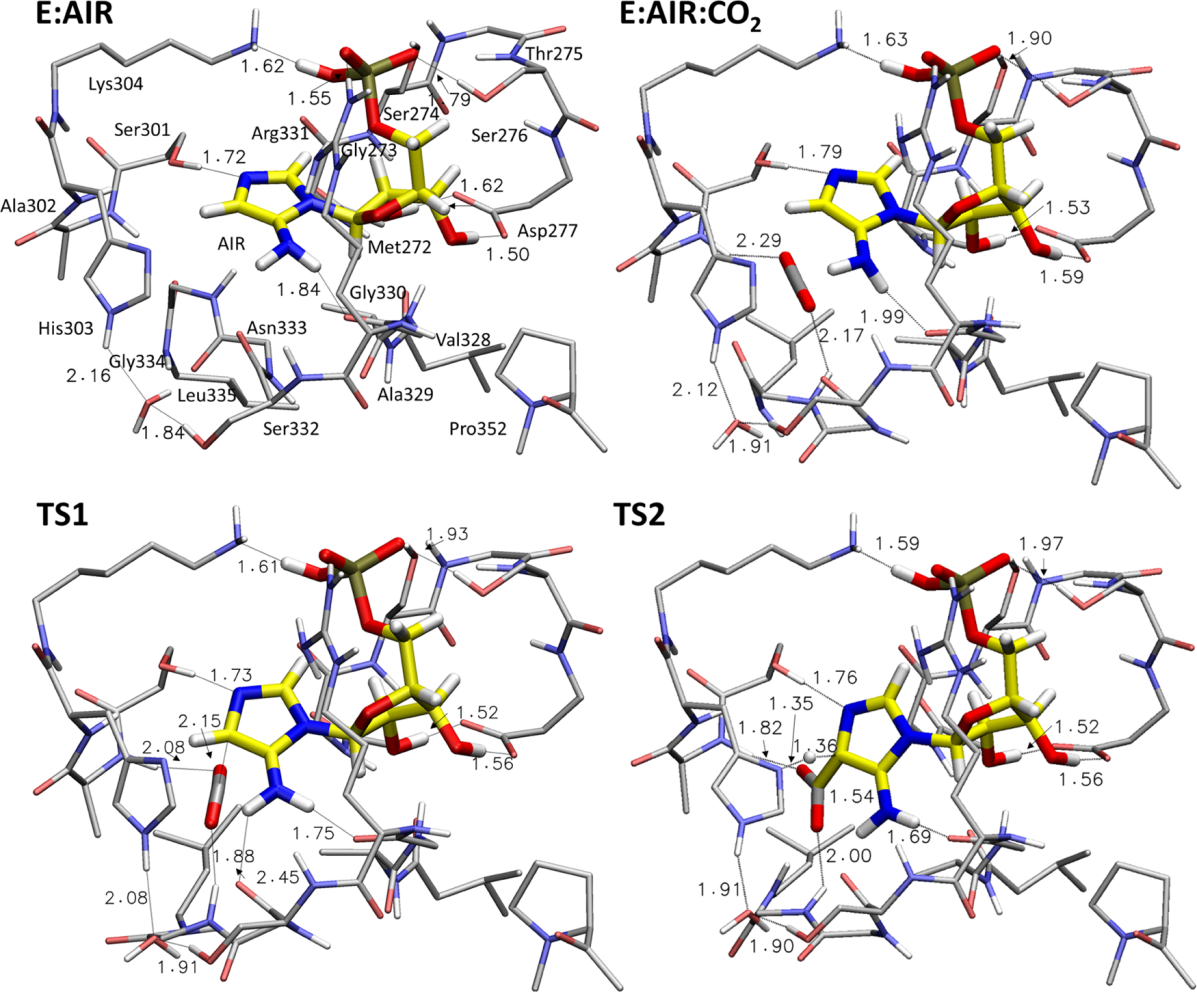
Optimized structures of the enzyme–substrate **E:AIR** and **E:AIR:CO_2_** complexes, on the top, and of the transition states **TS1** and **TS2**, on the bottom. Selected distances are given in Å. For clarity, most of the hydrogens are omitted.

The energy profile of the carboxylation step is depicted in Figure 3. In the optimized enzyme–substrate complex (**E:AIR**), the aminoimidazole ring of the substrate is involved in hydrogen bonds with the side chain of Ser301 and the backbone carbonyl of Gly330, while the hydroxyl groups of the ribose ring interact with the carboxylate moiety of Asp277 (see Figure 4). The phosphate group of the substrate is surrounded by the side chain of Lys304 and by the hydroxyl groups of Ser274 and Ser276, thus maintaining a similar conformation as in the crystal structure (see superposition in Figure S2). Comparison of the structures reveals that the guanidinium group of Arg331 shifts slightly with respect to its position in the crystal structure, favoring the interaction with the phosphate group of **AIR**. We also note that since the **AIR** substrate lacks the carboxylate moiety as compared to the **CAIR** product in the crystal structure, the positions of Asn333, Gly334, and Leu335 in the **E:AIR** complex are somewhat shifted compared to that structure (Figure S2).

Next, **CO_2_** binds to the AIRc active site. The calculations show that it can bind in different positions relative to the **AIR** substrate, *i.e.*, either in the pocket occupied by the carboxylate moiety of the **CAIR** product in the crystal structure (the complex is called **E:AIR:CO_2_**, Figure 4) or on the other side of the aminoimidazole ring toward the solvent-exposed area (called **E:AIR:CO_2_′**, see Figure S3). **E:AIR:CO_2_′** is energetically more favorable than **E:AIR:CO_2_** (+5.8 *vs* +14.9 kcal/mol, respectively, compared to the **E:AIR** complex). However, although **E:AIR:CO_2_** is higher in energy, it is the only productive binding mode, as C–C bond formation starting from **E:AIR:CO_2_′** is associated with prohibitively high energies (see Figure S3).

In **E:AIR:CO_2_**, the **CO_2_** binding site is located at the bottom of the active site pocket, below the bound **AIR**. The oxygen atoms of **CO_2_** engage in interactions with the amide groups of Ala302 and Gly334 (Figure 4). The presence of **CO_2_** causes some shift in the positions of the amino acids of the binding site of the gas molecule, without substantially altering the hydrogen-bonding interactions of the **AIR** substrate, as can be seen from the superposition of the optimized **E:AIR** and **E:AIR:CO_2_** structures provided in Figure S4. Furthermore, the position of **CO_2_** in its binding site in the **E:AIR:CO_2_** model is in good agreement with the structure of *Acetobacter aceti* PurE-II in complex with **AIR** and **CO_2_** (PDB 5CLJ).

From **E:AIR:CO_2_**, the carbon dioxide is next attacked by the C=C double bond of **AIR** with a barrier (**TS1**) of 3.0 kcal/mol relative to **E:AIR:CO_2_** (*i.e.*, 17.9 kcal/mol relative to **E:AIR**), generating **INT1**, which lies at +10.1 kcal/mol. Upon C–C bond formation, the aminoimidazole ring of the substrate in **INT1** is tetrahedral at the C^4^ position and represents thus the nonaromatic form of **CAIR** (named **isoCAIR**). This species has been suggested as an intermediate in the second half-reaction of PurE-I^13l^ and has also been implicated in the nonenzymatic decarboxylation of **CAIR**.^22^ In **INT1**, two hydrogen-bonding interactions between the formed carboxylate group and the amide NH groups of the peptide bonds of Ala302 and Gly334 help to stabilize the intermediate (see structures in Figure S5).

In the next step of the reaction, a proton transfer takes place from the C^4^ of the substrate to His303, resulting in the enzyme–product complex **E:CAIR**. The calculated energy barrier for this step (**TS2**) is +18.0 kcal/mol and **E:CAIR** is 10.1 kcal/mol higher than **E:AIR**. The conformations of the active site residues in **E:CAIR** are in agreement with the available X-ray structure of human PAICS, as can be seen from the superposition of the two structures given in Figure S6.^13^ A small deviation with respect to crystallographic data is observed for the side chain of Arg331, which moves closer to the phosphate group of the substrate (see Figure S6).

The barriers for the C–C bond formation (**TS1**) and the deprotonation (**TS2**) steps are very close (17.9 and 18.0 kcal/mol, respectively), and it is therefore not possible to determine on the basis of the calculations which step is the rate-limiting one for this part of the reaction. Since there are no experimental rate constants available for human PAICS, a direct comparison of the calculated barriers with experiments is not possible either. However, *k*_cat_ values of 32 and 77 s^–1^ have been measured for the AIRc domain of chicken PAICS and for type II PurE of *Treponema denticola*, respectively.^13o^ These values correspond to a barrier of *ca*. 15 kcal/mol, which is in good agreement with the barriers calculated here.

The calculations highlight the catalytic role of His303 as a general base in the second step of the reaction. Inspection of the AIRc active site shows that this residue is the only one that can act as a proton acceptor in the proximity of the substrate. Consistently with this observation, this histidine is universally conserved in all class I and class II PurE enzymes.^13h,13o^ The importance of the His303 residue for the carboxylation of **AIR** is also in line with functional complementation experiments carried out using human PAICS mutants introduced into a ΔpurK strain of *Escherichia coli*, where the introduction of the His303Tyr mutation was linked to the absence of bacterial growth.^13m^ Similar experiments were also done with chicken PAICS, where the Ser302Ala/His304Tyr double mutant (corresponding to human PAICS residues Ser301 and His303) abolished the complementation.^23^ Furthermore, inactivation of the homologous type I PurE mutase from *E. coli* has been observed in the case of active site His mutations to Asn, Gln, and Trp in mutational experiments combined with enzymatic and functional complementation assays.^13l^ Similar experiments conducted on PurE-I from *A. aceti* showed complete inactivation of the enzyme when the His was mutated to Asn, Phe, Ala, or Ser, while only traces of activity were obtained when it was mutated to Asp and Gln.^13h^

In addition to the mechanism reported in Scheme 1, we have also investigated two additional mechanistic proposals. It has been proposed that the carboxylation reaction proceeds *via* an ylide mechanism, *i.e.*, starting with the **AIR** substrate being in the ylide form, with a protonated N^3^ and a deprotonated C^4^. This proposal was put forward on the basis of evidence from nonenzymatic carboxylation of **AIR**.^13,14,24^ To examine this mechanism, we calculated the energy of the proposed ylide intermediate both in solution and inside the active site model. It turns out that it is as much as 44 kcal/mol higher than the **AIR** substrate in the solution and 41 kcal/mol higher in the enzyme (see Figure S7). These results are thus sufficient to rule out the ylide mechanism. This is consistent with previous studies on PurE intermediate analogs^14^ and also with the study on the prokaryotic class II PurE from *T. denticola* that suggested that **AIR** does not get protonated at the N^3^ position at neutral pH and remains a hydrogen bond acceptor of the conserved Ser301 during the entire carboxylation reaction.^13o^

Finally, we also carried out additional calculations to investigate whether the carboxylation of **AIR** can take place through the reaction with bicarbonate (**HCO_3_^–^**) instead of **CO_2_**. The calculations showed that this mechanistic scenario is also associated with very high energies and can be ruled out (see Figure S8 for details).

To summarize this section, we can conclude that the carboxylation occurring *via* the **isoCAIR** mechanism^13l,14^ is associated with feasible energy barriers. We anticipate that the results and the mechanistic details provided by the current calculations for human PAICS can be extended to both class I and class II PurE enzymes in general.^12d,13h,13l,14^

### 3.2 Phosphorylation–Condensation in the SAICARs Active Site

After the carboxylation, it has been suggested that **CAIR** moves from the AIRc to the SAICARs active site without being released into the solution, thus becoming the substrate of the phosphorylation–condensation reaction.^13u^ As observed in other enzymes with multiple catalytic sites, this often takes place *via* an intermediate tunnel system that connects the active sites.^25^ In the case of human PAICS, the channel was recently recognized by inspection of the octameric structure of the bifunctional enzyme.^13m,13u^

In the SAICARs catalytic pocket, **CAIR** is first phosphorylated in a reaction with **ATP** before the reaction with **Asp**, acting analogously to adenylosuccinate synthetase.^13j,15^ Although the addition of the individual substrates to the active site has been shown to be random,^15^ it has also been suggested that **Asp** and **ATP** saturate the SAICARs active site before the binding of **CAIR**(15) and that the recognition of **ATP** by SAICARs might facilitate the binding of **CAIR**.^13j^ In the model, we include all three components (**ATP**, **CAIR**, and **Asp**) from the start of the reaction. As discussed above, three Mg ions are included in the model in analogy with the SAICARs:ADP:CAIR structure from *E. coli*.^13^

**Figure 5:**
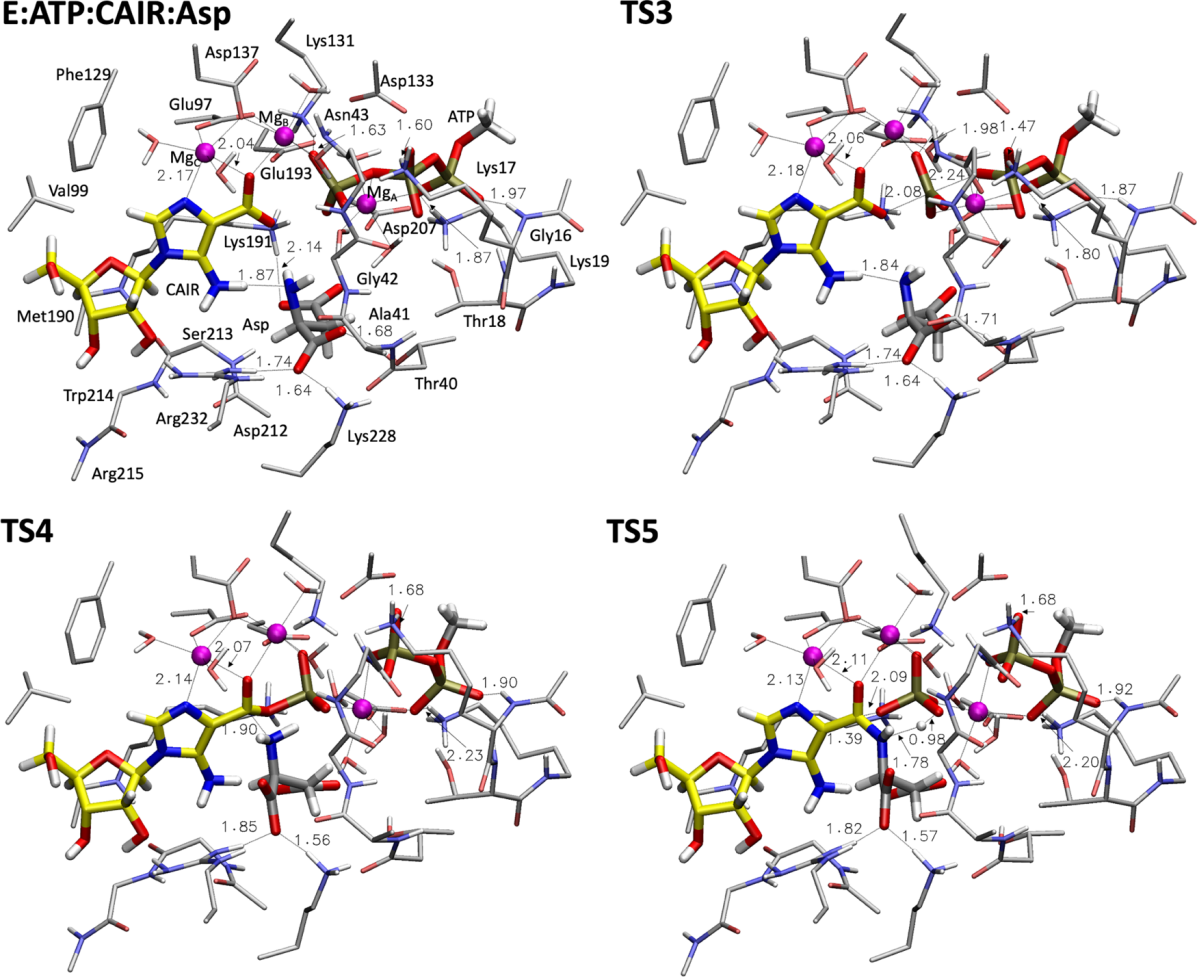
Optimized structures of the enzyme–substrate complex **E:ATP:CAIR:Asp** and the following transition states for the phosphorylation–condensation reaction. Selected distances are given in Å. For clarity, most of the hydrogens are omitted.

In the optimized **E:ATP:CAIR:Asp** complex, the carboxyl group and the N^3^ of the aminoimidazole ring of the **CAIR** substrate coordinate Mg*_C_*. It is likely that **CAIR** associates with Mg*_C_* on its way from the AIRc to the SAICARs active site, because SAICARs recognizes the substrate in a complex with magnesium.^13j^ The Mg*_C_* ion is additionally coordinated by Glu97, Asp137, and two water molecules. The Glu97 and Asp137 residues are further bound to Mg*_B_*, Asp137 bridging the two ions in a μ(1,1) fashion, while Glu97 bridging in a μ(1,3) fashion. The γ-phosphate of **ATP** and two water molecules complete the coordination sphere of Mg*_B_*. The **ATP**, which coordinates to Mg*_A_* with the oxygens from the α-, β-, and γ-phosphates, is involved in hydrogen-bonding interactions with the backbones and side chains of a number of amino acids, such as Lys19, Asn43, and Lys131 (see Figure 5). The above interactions are preserved in all intermediates and transition states throughout the reaction and match well with those observed in X-ray structures of ternary complexes PAICS:AMP-PNP:SAICAR^13u^ and *E. coli* SAICARs:ADP:CAIR,^13j^ with two and three Mg ions, respectively. Important distances of the coordination spheres of the Mg ions are provided in Table S1.

The two carboxylate moieties of the **Asp** substrate are involved in hydrogen bonds with the Arg232, Lys228, Thr40, and Lys191 residues, which constitute the binding region of the substrate, as also observed in the SAICARs:Asp complex of *Saccharomyces cerevisiae* (PDB 2CNU). The amino group of **Asp** interacts with the N^5^ amino group of **CAIR**, suggesting that the latter can play a role in the recognition of **Asp**. This is consistent with a previous hypothesis that SAICARs enzymes recognize the amino group of **Asp** to discriminate the substrate from other dicarboxylic acids, like the intermediates of the citric acid cycle.^15^ Interestingly, the calculations show that such recognition is mediated through the **CAIR** substrate rather than through the active site residues.

It is interesting to mention here that we have also considered an alternative binding mode of the **ATP** in the **E:ATP:CAIR:Asp** complex where Mg*_B_* is coordinated by two phosphates (**E:ATP:CAIR:Asp′**, Figure S9). Such binding mode would be analogous to the crystal structures of enzymes having a similar fold but binding only two Mg ions (*e.g.*, inositol phosphate multikinase and inositol hexakisphosphate kinase, PDB codes 5W2I and 4O4D, respectively).^26^ However, the energy was found to be more than 5 kcal/mol higher than the **E:ATP:CAIR:Asp** complex, mainly due to differences in the hydrogen-bonding network of the α- and β-phosphate groups (see Figure S9).

**Figure 6:**
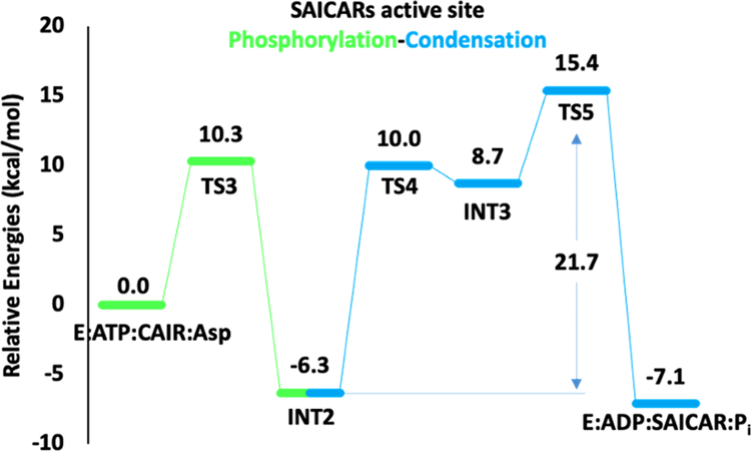
Calculated energy profile for the phosphorylation–condensation reaction.

The present calculations show that the phosphorylation proceeds through a concerted S*_N_*2 attack of the carboxylate moiety of **CAIR** on the P^γ^ of **ATP**, in agreement with the proposal on the phosphorylation of **CAIR** for the *E. coli* SAICARs.^13j^ The transition state for this step (**TS3**, Figure 5) has an energy of +10.3 kcal/mol relative to the E**:ATP:CAIR:Asp** complex (see Figure 6). The forming O–P^γ^ and the breaking P^γ^–O^β^ bonds at **TS3** are 2.08 and 2.24 Å, respectively (Figure 5).

Interestingly, initial attempts to locate **TS3** using models that did not include the Mg*_A_* and Mg*_B_* ions and the amino acids composing the **ATP** binding site were not successful and resulted in the return to **E:ATP:CAIR:Asp**. We attribute such behavior to the absence of the positive charge that stabilizes the negative charges of the oxygen atoms of the α- and β-phosphates of the nascent **ADP**. A large active site model was therefore necessary to study the phosphorylation reaction.

In **INT2**, which is 6.3 kcal/mol below the reactants, the phosphorylated **CAIR** (**phospho–CAIR**) is formed and can react with **Asp** in the following condensation reaction. In this intermediate, the two carboxylate groups of **Asp** are engaged in hydrogen bonds with Thr40, Lys191, Lys228, and Arg232 (see Figure S10). The first step of the condensation reaction (**TS4**), in which the formation of the N_Asp_–C^4^_CAIR_ bond takes place, has a barrier of 16.3 kcal/mol, with respect to **INT2**, and the resulting tetrahedral intermediate **INT3** is 15.0 kcal/mol higher than **INT2**. The protonated N_Asp_ in **INT3** is involved in the hydrogen-bonding interaction with the oxygen of the γ-phosphate. Importantly, the formed oxyanion is stabilized by binding to both the Mg*_C_* and Mg*_B_* ions (see structures in Figure S10).

In **TS5**, the last step of the condensation reaction, the dephosphorylation takes place by a concerted cleavage of C^4^_CAIR_–OP^γ^ bond and deprotonation of N_Asp_, forming the amide bond. The calculated barrier for this step is 21.7 kcal/mol relative to **INT2**. The products **SAICAR** and **HPO_4_^2–^** (**P_i_**) are finally obtained in the complex **E:ADP:SAICAR:P__i__**, with a favorable thermodynamics of −7.1 kcal/mol relative to **E:ATP:CAIR:Asp** (see Figure 6).

Comparison of the optimized geometry of **E:ADP:SAICAR:P__i__** with the available crystal structure of PAICS:AMP-PNP:SAICAR^13u^ reveals some interesting differences that are mainly due to the lack of Mg*_B_* and **P_i_** in the crystal structure (superposition of the two structures is reported in Figure S11). The **P_i_** moiety in **E:ADP:SAICAR:P__i__** coordinates to all three Mg ions, which results in a different conformation of the **SAICAR** product in the active site as compared to the crystal structure. For example, the carboxylate groups of **SAICAR** are involved in hydrogen-bonding interactions with Lys228 and Thr40, which is not the case in the crystal structure. Consistently with this, the previous crystallographic analysis showed that the *N*-succinyl moiety of **SAICAR** is rather flexible, which might also be important for the release of the product from the active site.^13u^

The calculated energy profile in Figure 6 reveals that the final step, *i.e.*, the dephosphorylation (**TS5**), is the rate-determining step of the phosphorylation–condensation reaction. The overall barrier from **INT2** (21.7 kcal/mol) is in quite good agreement with the available *k*_cat_ of 3 s^–1^, which corresponds to a barrier of *ca*. 17 kcal/mol.^27^

We have further examined the possibility of a phosphorylation reaction taking place through a transfer of the γ-phosphate from **ATP** first to Glu193, then to Glu97, and finally to **CAIR**, in accordance with a proposal based on the crystal structure of SAICARs from *Streptococcus pneumoniae*.^13^ The calculations show that already the first intermediate in such a mechanism, *i.e.*, the phosphorylation of Glu193, is 16.3 kcal/mol higher than **E:ATP:CAIR:Asp** (see Figure S12). This mechanistic proposal can thus be ruled out since it is associated with a much higher energy than the direct phosphorylation of **CAIR***via***TS3**.

We have also tested an alternative mechanistic proposal in which the condensation reaction takes place before the phosphorylation (see Figure S13).^13j^ The first intermediate in such a mechanism corresponds to a geminal diolate on the C^4^ center of **CAIR**. Geometry optimization of such species was not possible and always returned to the **E:ATP:CAIR:Asp** complex. However, a constrained optimization with a fixed C^4^_CAIR_–N_Asp_ has an energy of more than 30 kcal/mol higher than **E:ATP:CAIR:Asp**, *i.e.*, more than 15 kcal/mol higher in energy than the highest transition state (**TS5**). This result rules out the possibility of the condensation reaction taking place prior to the phosphorylation.

The current calculations show that the Mg ions catalyze the reaction by binding the **CAIR** and **ATP** moieties and stabilizing the negative charges that arise at the transition states and intermediates of the reaction. This role of the Mg ions can be linked to enzyme kinetics studies indicating that a higher number of magnesium cations than those needed for the saturation of **ATP** are required for catalysis.^13j,15^

Finally, it is interesting to point out that the proposed mechanism for the phosphorylation–condensation reaction (Scheme 1) does not require the direct involvement of any SAICARs active site residues. In related reactions, active site residues might act as nucleophiles or general acids/general bases. Instead, the amino acids of the SAICARs active site bind the substrates and provide the environment necessary to coordinate the Mg ions that facilitate the reaction.

## 4 Conclusions

In the present work, we have employed the quantum chemical cluster approach to study the detailed reaction mechanism of the bifunctional human PAICS enzyme. This enzyme is essential for the *de novo* purine biosynthesis and therefore constitutes a target for new cancer therapies.

The recently solved crystal structures of human PAICS in complex with **CAIR**, **SAICAR**, and **AMP-PNP**(13u) were used to design two models of the AIRc and SAICARs active sites, consisting of 269 and 369 atoms, respectively. Using these models, different mechanistic proposals were examined and compared in terms of energetic feasibility.

It is shown that in the case of carboxylation, the reaction proceeds *via* the “**isoCAIR** mechanism”, where **CO_2_** undergoes a nucleophilic attack of C^4^ of **AIR**. His303 acts then as a general base to form the **CAIR** product.

With regard to the phosphorylation–condensation reaction occurring in the SAICARs domain, the calculations show that the **CAIR** substrate must first be phosphorylated to react with **Asp**. The phosphorylation of **CAIR** takes place directly from **ATP**, by an S*_N_*2 mechanism. The **SAICAR** product is formed through a two-step condensation mechanism, involving first a C–N bond formation between **Asp** and the **phospho–CAIR** and then the dissociation of **P_i_**. According to the calculations, the last step is rate-limiting for the entire reaction. All three Mg ions are demonstrated to be directly involved in the substrate binding and the stabilization of the transition states and intermediates along the phosphorylation–condensation reaction.

We believe that the insights provided by the current calculations will be an important asset in the continued design of new, and the optimization of existing, inhibitors of human PAICS for applications in anticancer therapies.

## Supporting Information Available

Additional results discussed in the text; absolute energies; and Cartesian coordinates of optimized geometries (PDF)

## Abbreviations

AIR: aminoimidazole ribonucleotide
AIRc: AIR carboxylase
AMP-PNP: adenosine 5′-(β,γ-imido)triphosphate
CAIR: carboxyaminoimidazole ribonucleotide
PAICS: phosphoribosylaminoimidazole carboxylase and phosphoribosylaminoimidazolesuccinocarboxamide synthetase
SAICAR: *N*-succinylcarboxamide-5-aminoimidazole ribonucleotide
SAICARs: SAICAR synthetase
