# Supplementary material for: Reaction Mechanism of Human PAICS Elucidated by Quantum Chemical Calculations
Source: J Am Chem Soc. 2022 Aug 1;144(31):14258–68. doi: 10.1021/jacs.2c05072 (PMC9376930; doi:10.1021/jacs.2c05072)
Supplement: Supplementary file 1 — ja2c05072_si_001.pdf [file ja2c05072_si_001.pdf]

Supporting Information

Reaction Mechanism of Human PAICS Elucidated by Quantum Chemical Calculations

Mario Prejanò,[a] Jana Škerlová,[b] Pål Stenmark[c] and Fahmi Himo[a] *

[a] *Department of Organic Chemistry, Arrhenius Laboratory, Stockholm University, SE-10691 Stockholm, Sweden.*

[b] *Institute of Organic Chemistry and Biochemistry, Czech Academy of Sciences, Flemingovo nam. 2, 160 00 Prague, Czech Republic.*

[c] *Department of Biochemistry and Biophysics, Stockholm University, SE-10691 Stockholm, Sweden.*

* Corresponding author: fahmi.himo@su.se

Contents

1. The SAICARs active site model. ***S3***

2. Results on the carboxylation mechanism. ***S4***

2.1. Superposition of **E:AIR** with the crystal structure. S4

2.2. Alternative binding mode of **CO_2**. S5

2.3. Superposition of **E:AIR** and **E:AIR:CO_2**. S6

2.4. Structures of **INT1** and **E:CAIR**. S7

2.5. Superposition of **E:CAIR** with the crystal structure. S8

2.6. Results on the alternative ylide mechanism. S9

2.7. Results on the alternative mechanism involving bicarbonate. S10

3. Results on the phosphorylation-condensation mechanism. ***S11***

3.1. Alternative binding mode of **ATP**. S11

3.2. Structures of **INT2**, **INT3** and the **E:ADP:SAICAR:P_i** complex. S12

3.3 Superposition of **E:ADP:SAICAR:P_i** with the crystal structure. S13

3.4 The alternative stepwise phosphorylation mechanism. S14

3.5. Results on the condensation-first mechanism. S15

3.6. Metal-ligand bond distances. S16

4. Absolute and relative energies. ***S17***

5. Cartesian coordinates ***S18***

1. The SAICARs active site model.

In the crystal structure of human PAICS in complex with **SAICAR** and **AMP-PNP** (PDB 6YB9), the Mg_B ion is absent. Instead, a water molecule (w1) takes its place and interacts with Asp137, Glu97 and w2 (Figure S1). In the active site model, Mg_B was manually inserted in the place of w1, in accordance with the crystal structure of SAICARs in complex with **ADP** and **CAIR** isolated from *E. coli* (PDB 2GQS). In the final model, Mg_B is thus coordinated to Asp137 and Glu97 that bridge the Mg_B and Mg_C in a μ(1,1) and μ(1,3) fashion, respectively. The **CAIR** substrate, the **ATP** cofactor, and two water molecules complete the coordination sphere of Mg_B.

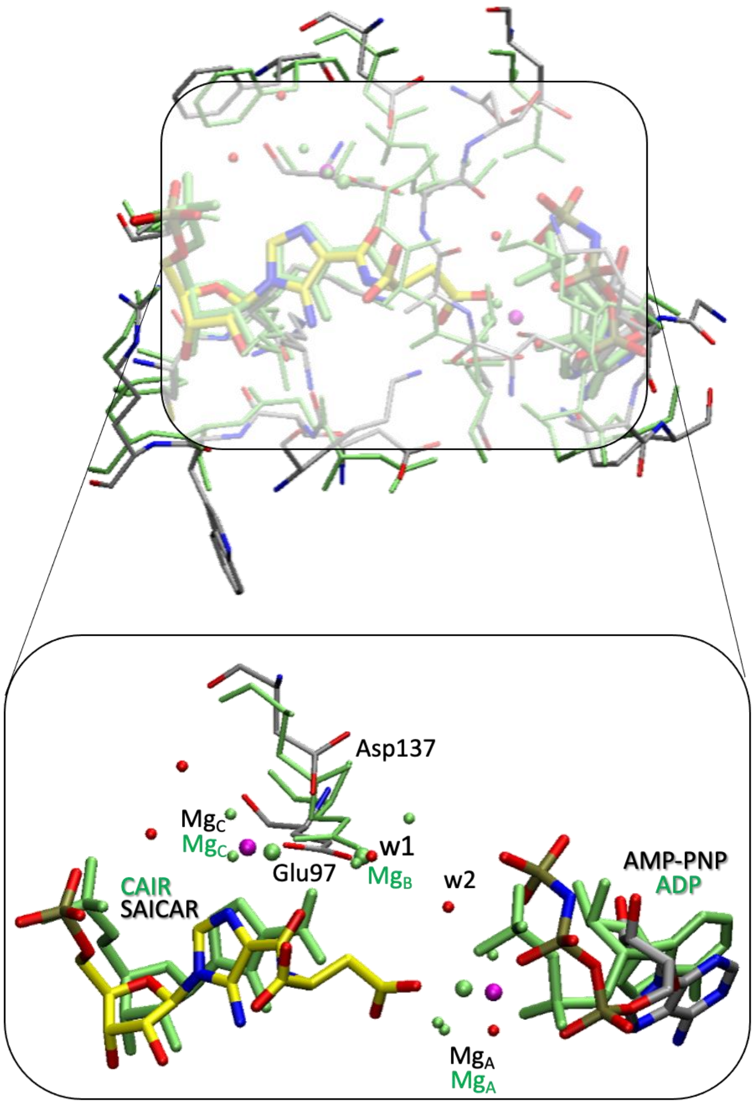

Figure S1. *Superposition of the SAICARs:AMP-PNP:SAICAR (PDB 6YB9) and SAICARs:ADP:CAIR (PDB 2GQS, green color) crystal structures.*

2. Results on the carboxylation mechanism.

*2.1. Superposition of **E:AIR** with the crystal structure.*

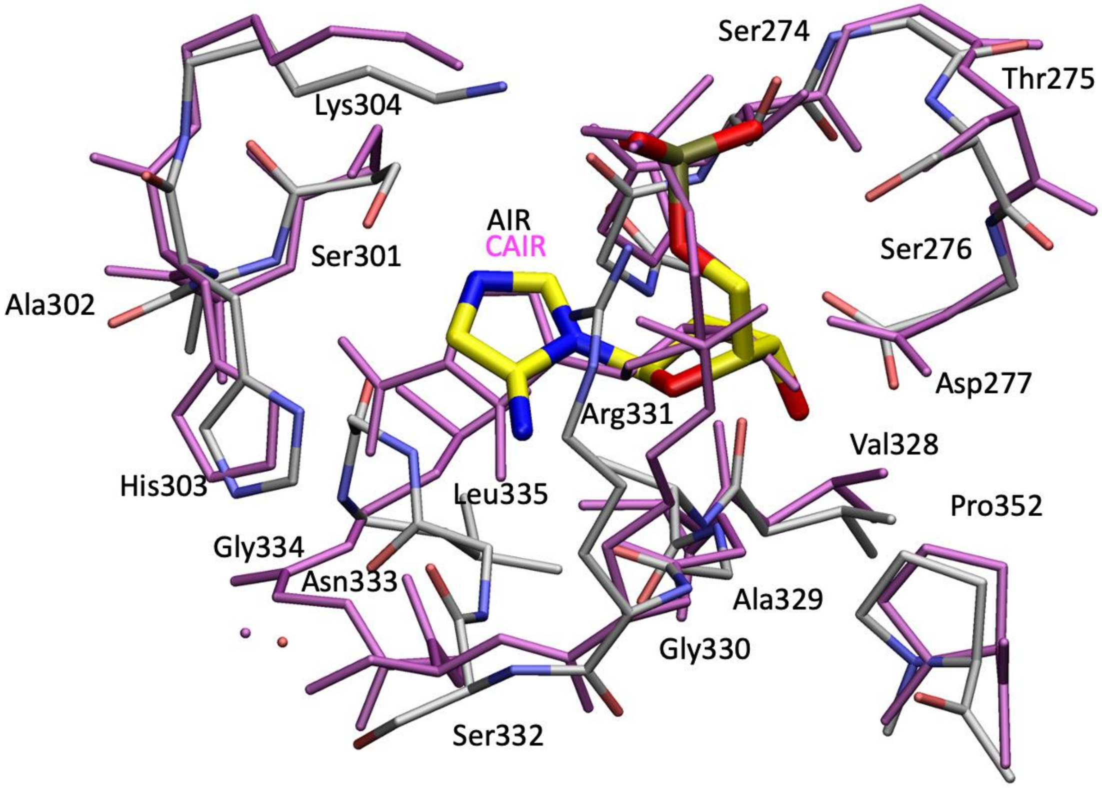

Figure S2. *Superposition of the optimized structure of complex* ***E:AIR*** *with the crystal structure (PDB 6YB8, in pink).*

2.2. Alternative binding mode of ***CO_2*****.**

As discussed in the main text, **CO_2** can bind on the other side of the **AIR** substrate as compared to **E:AIR:CO_2**. The optimized structure of this complex (**E:AIR:CO_2’**) is shown in Figure S3 below. Starting from this binding mode, the transition state for the C-C bond formation (**TS1’**) and the resulting intermediate (**INT1’**) were optimized and their energies were evaluated. The calculations show that this pathway is associated with prohibitively high energies and can thus be discarded. The main reason for the high energies is that the nascent carboxylate group lacks many of the stabilizing hydrogen bonds that are present in **TS1** and **INT1**.

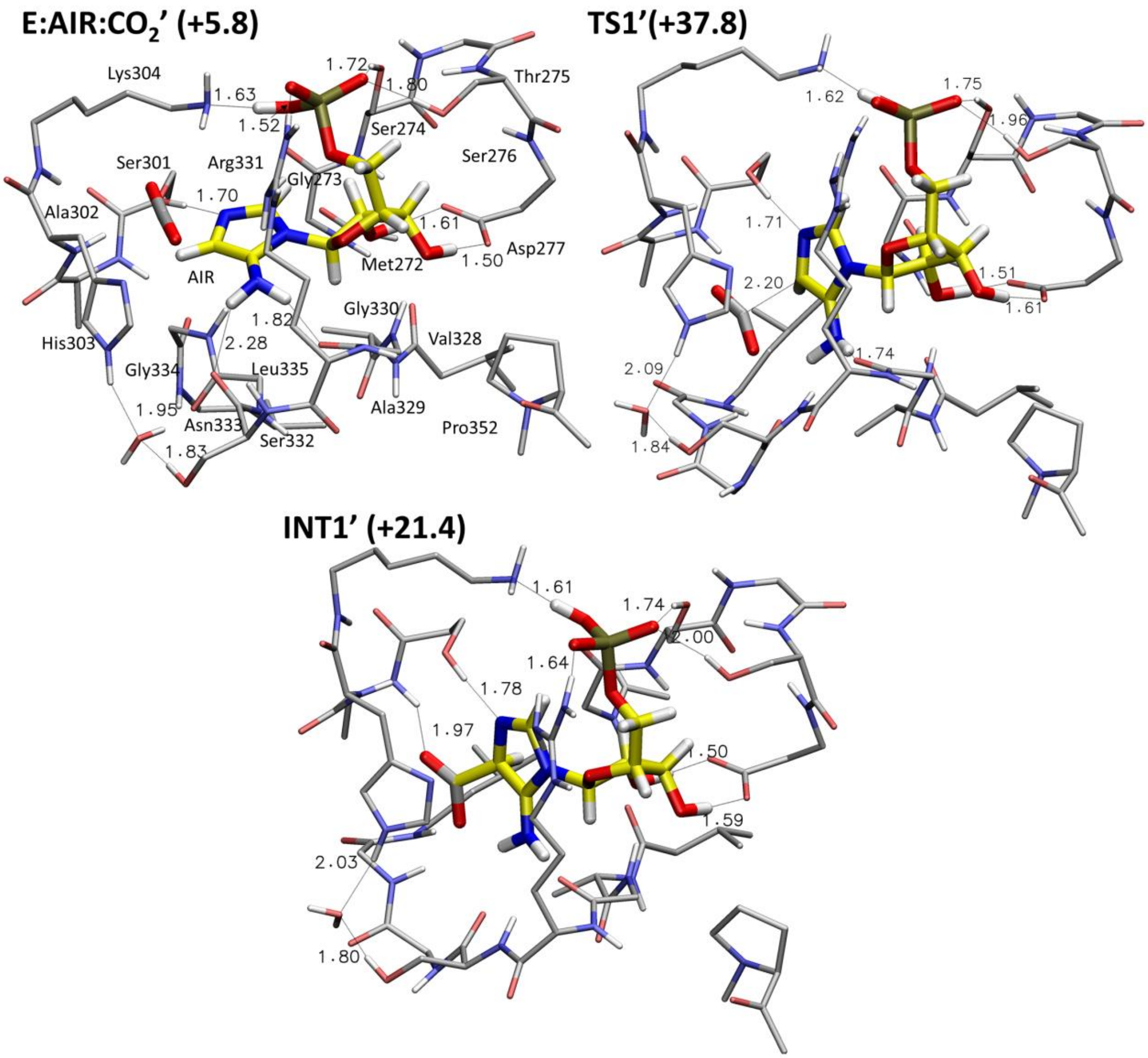

Figure S3. *Optimized structures of* ***E:AIR:CO_2’****,* ***TS1’****and* ***INT1’****. Selected distances are given in Å. For clarity, most of the hydrogens are omitted. Energies are given in kcal/mol relative to* ***E:AIR****.*

*2.3. Superposition of **E:AIR** and **E:AIR:CO_2**.*

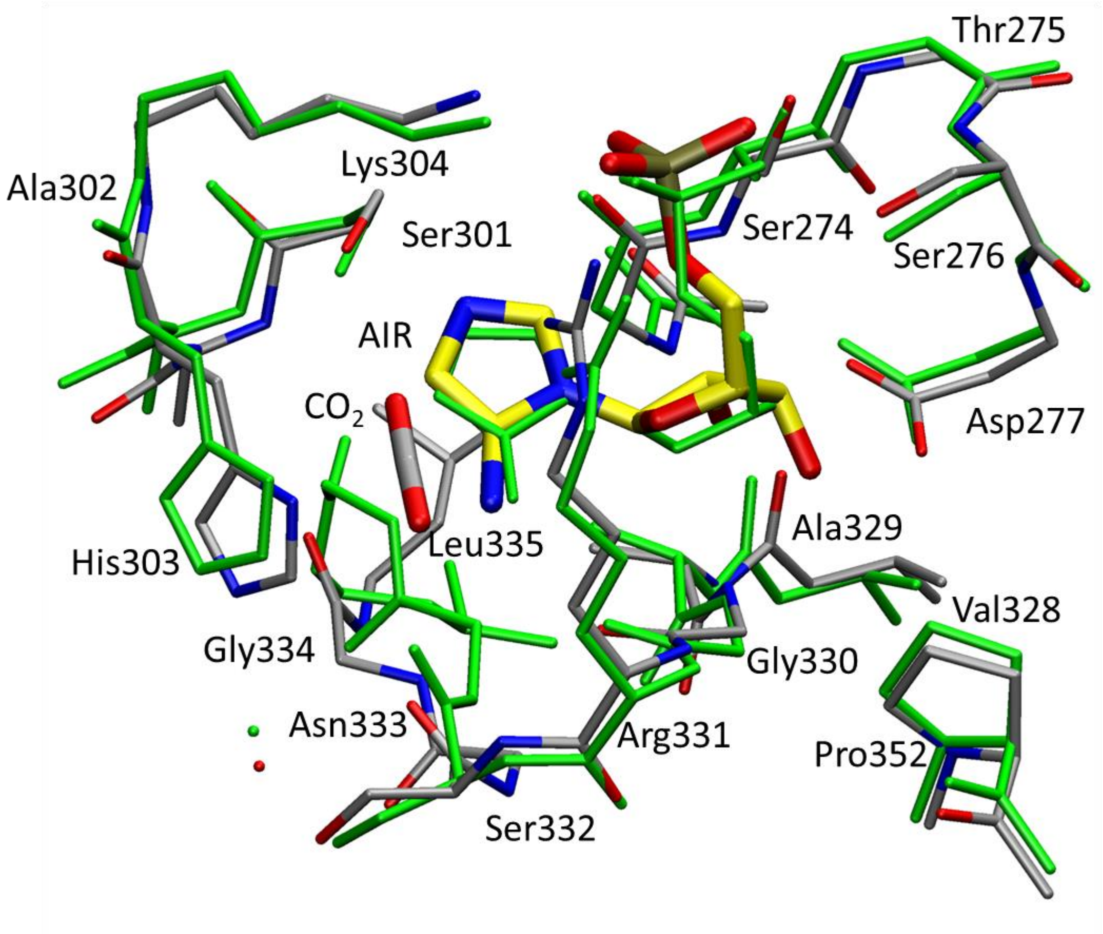

Figure S4. *Superposition of the **E:AIR** (green) and **E:AIR:CO_2** complexes.*

*2.4. Structures of **INT1** and **E:CAIR**.*

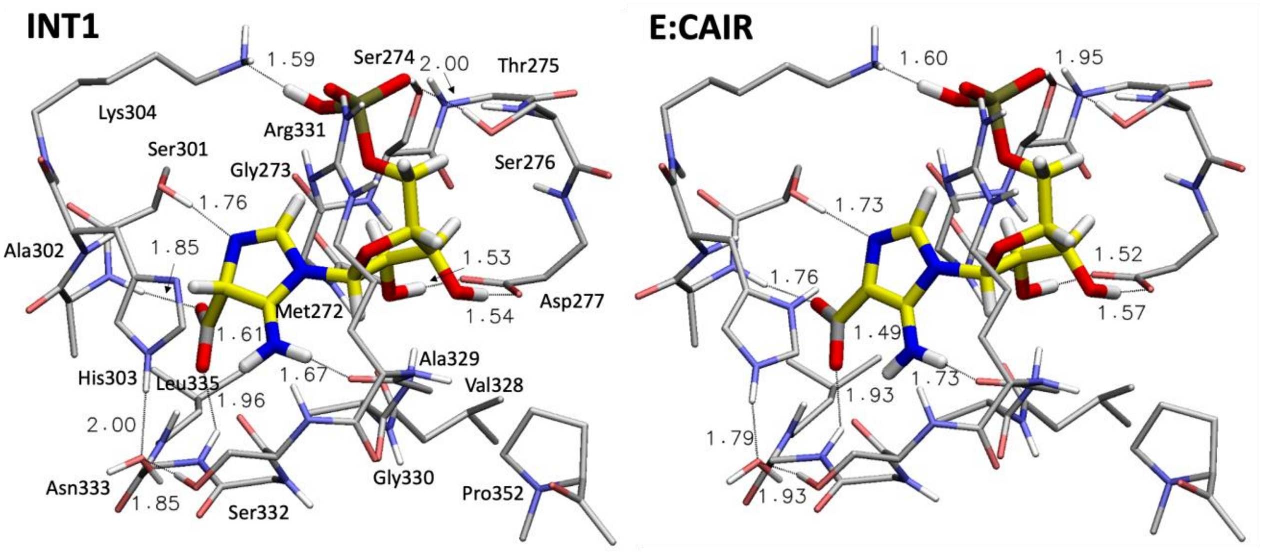

***Figure S5.** Optimized structures of **INT1** and **E:CAIR**. Selected distances are given in Å. For clarity, most of the hydrogens are omitted.*

*2.5. Superposition of **E:CAIR** with the crystal structure.*

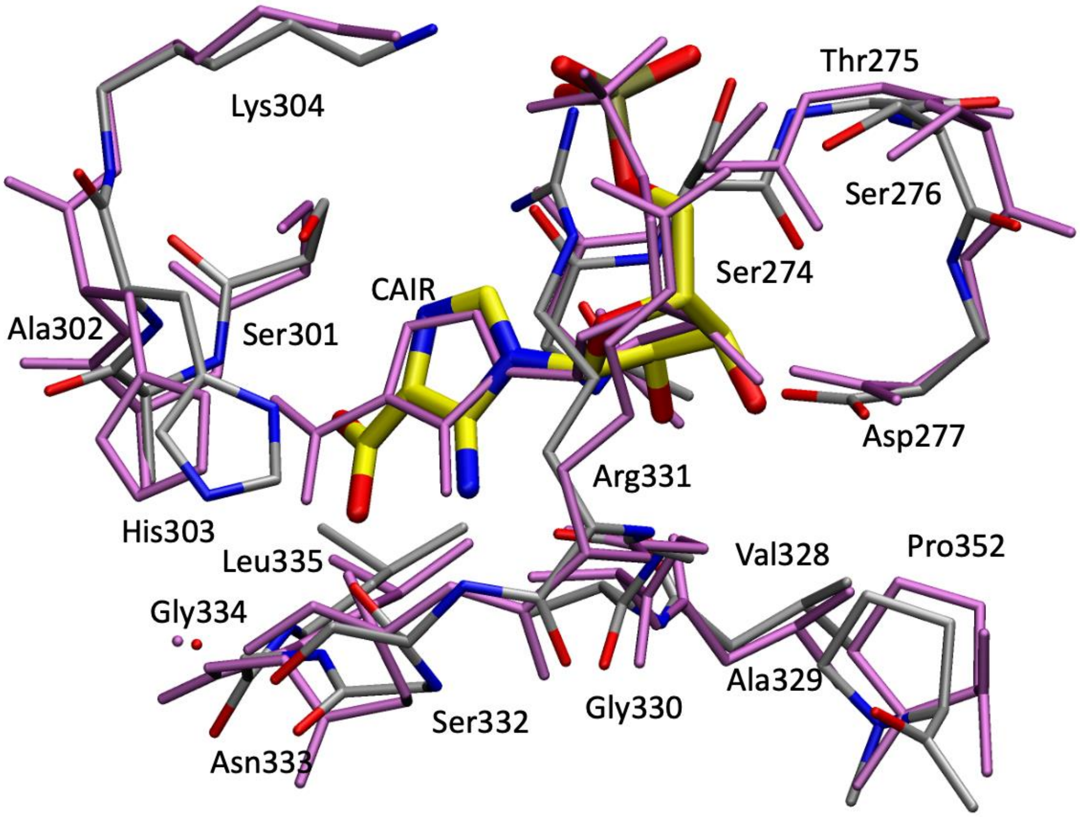

Figure S6. *Superposition of the optimized structure of complex **E:CAIR** with the crystal structure (PDB 6YB8, in pink).*

2.6. Results on the alternative ylide mechanism.

As discussed in the main text, it has been suggested that the carboxylation reaction can proceed starting with the **AIR** substrate being in the ylide form. As shown in Figure S7, in solution the ylide form of a model of **AIR**, in which the phosphoribosyl group is replaced by a methyl, is as much as 44.2 kcal/mol higher in energy than the **AIR** substrate. In the enzyme model, the ylide is 41.4 kcal/mol higher in energy. The ylide mechanism can thus be ruled out.

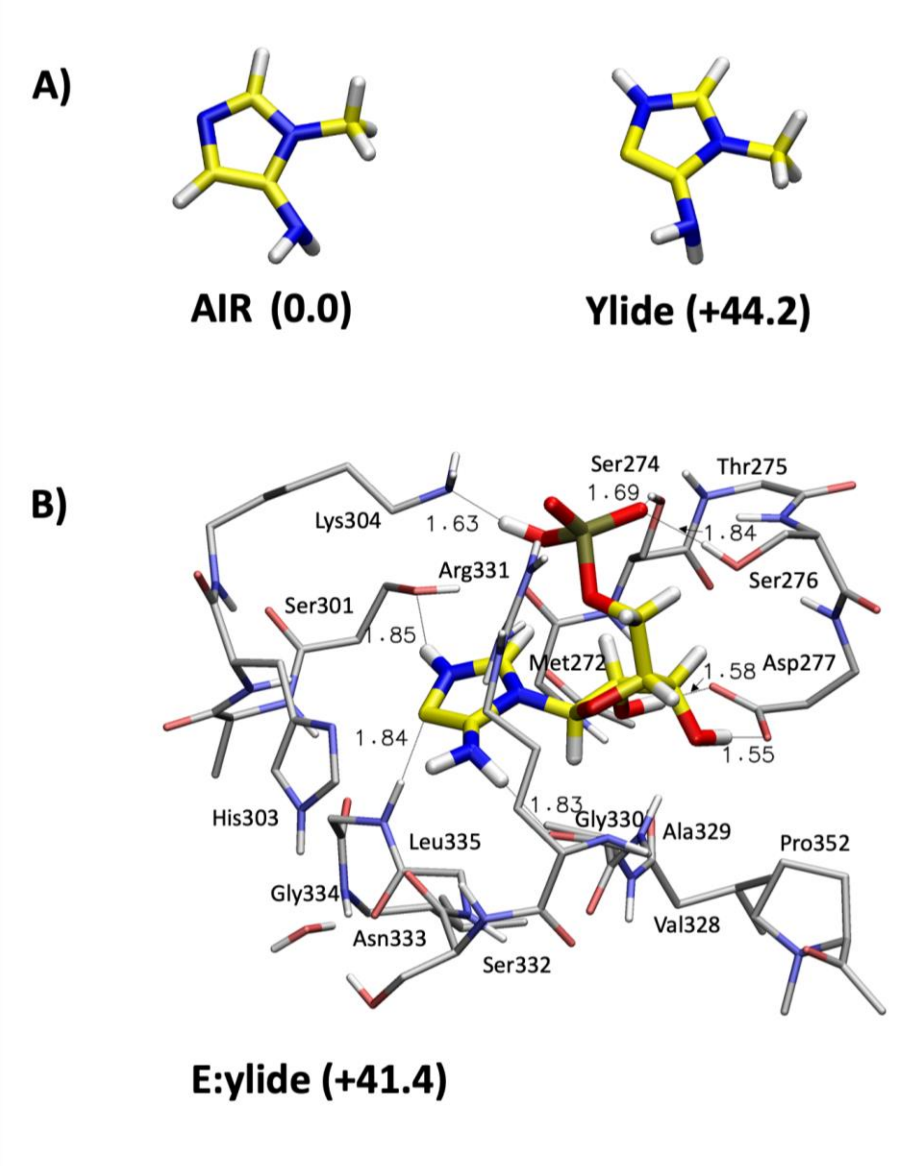

Figure S7. *A) Structures of* ***AIR*** *and the ylide form of a model substrate in solution, with relative energies indicated in kcal/mol. B) Optimized structure of the* ***E:ylide*** *enzyme model. Energy is given in kcal/mol relative to* ***E:AIR****.*

2.7. Results on the alternative mechanism involving bicarbonate.

To examine the energetic feasibility of **AIR** carboxylation conducted by **HCO_3^-**, the **E:AIR:HCO_3^-** complex was optimized by replacing the **CO_2** molecule in the productive binding mode (**E:AIR:CO_2**) with an **HCO_3^-** molecule (Figure S8A). Neither a transition state nor an intermediate for the C_{AIR}-C_{HCO3^-} bond formation could be located. However, constrained optimizations with a fixed C_{AIR}-C_{HCO3^-} bond distance showed that the energy increases to prohibitively high values (Figure S8B), which rules out this mechanistic scenario.

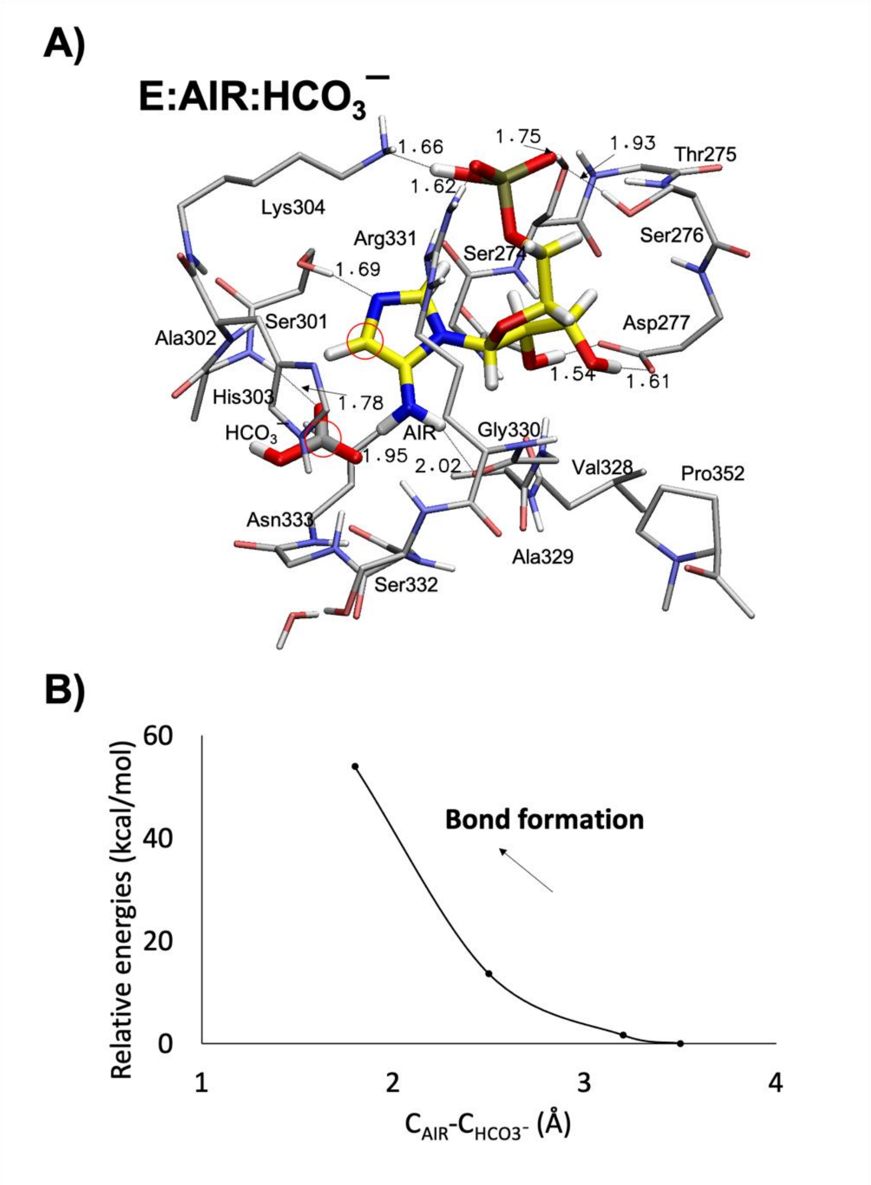

Figure S8. *A) Optimized structure of the **E:AIR:HCO_3^-** complex. B) Energy profile for the C-C bond formation. Note that the relative energies here are calculated only at the level of the geometry optimization.*

3. Results on the phosphorylation-condensation mechanism.

3.1. Alternative binding mode of ***ATP****.*

We have optimized an alternative starting complex in which the Mg_B ion is coordinated by two phosphate groups of **ATP** (Figure S9). The energy of this structure is calculated to be 5.6 kcal/mol higher than the structure in which only one phosphate is coordinated to MgB.

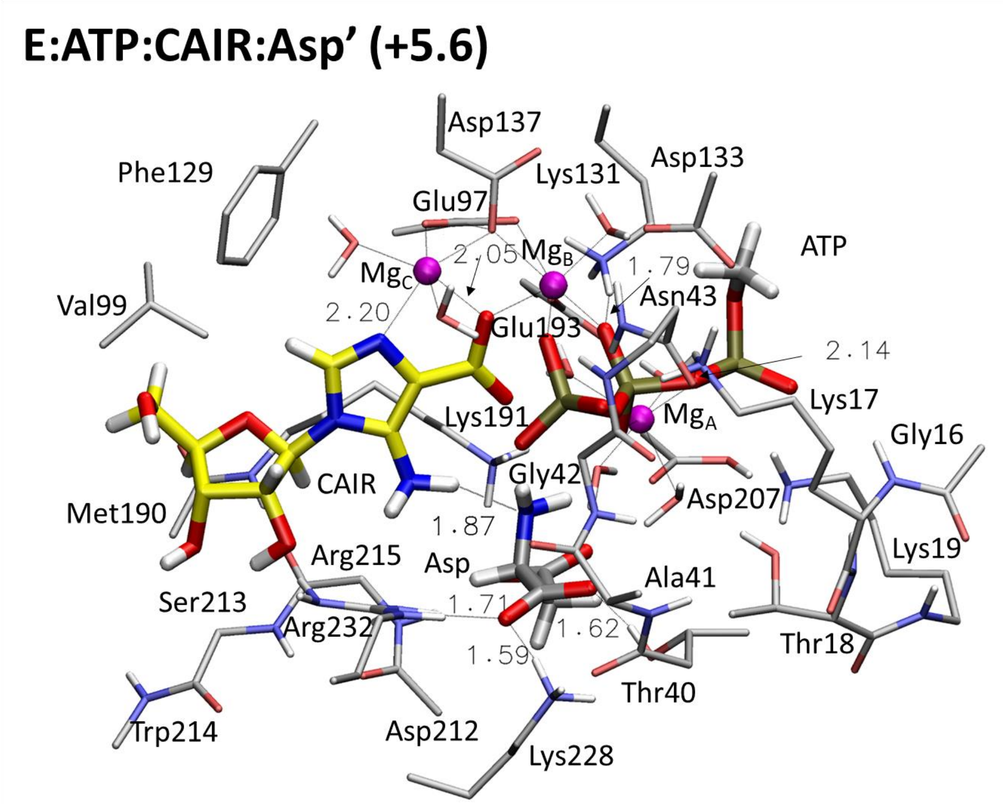

Figure S9. *Optimized structures of* ***E:ATP:CAIR:Asp'*** *complex with the alternative binding mode of* ***ATP****. Selected distances are given in Å. Energy (in kcal/mol) is given relative to* ***E:ATP:CAIR:Asp****. For clarity, most of the hydrogens are omitted.*

*3.2. Structures of **INT2**, **INT3** and the **E:ADP:SAICAR:P**$_i$ complex.*

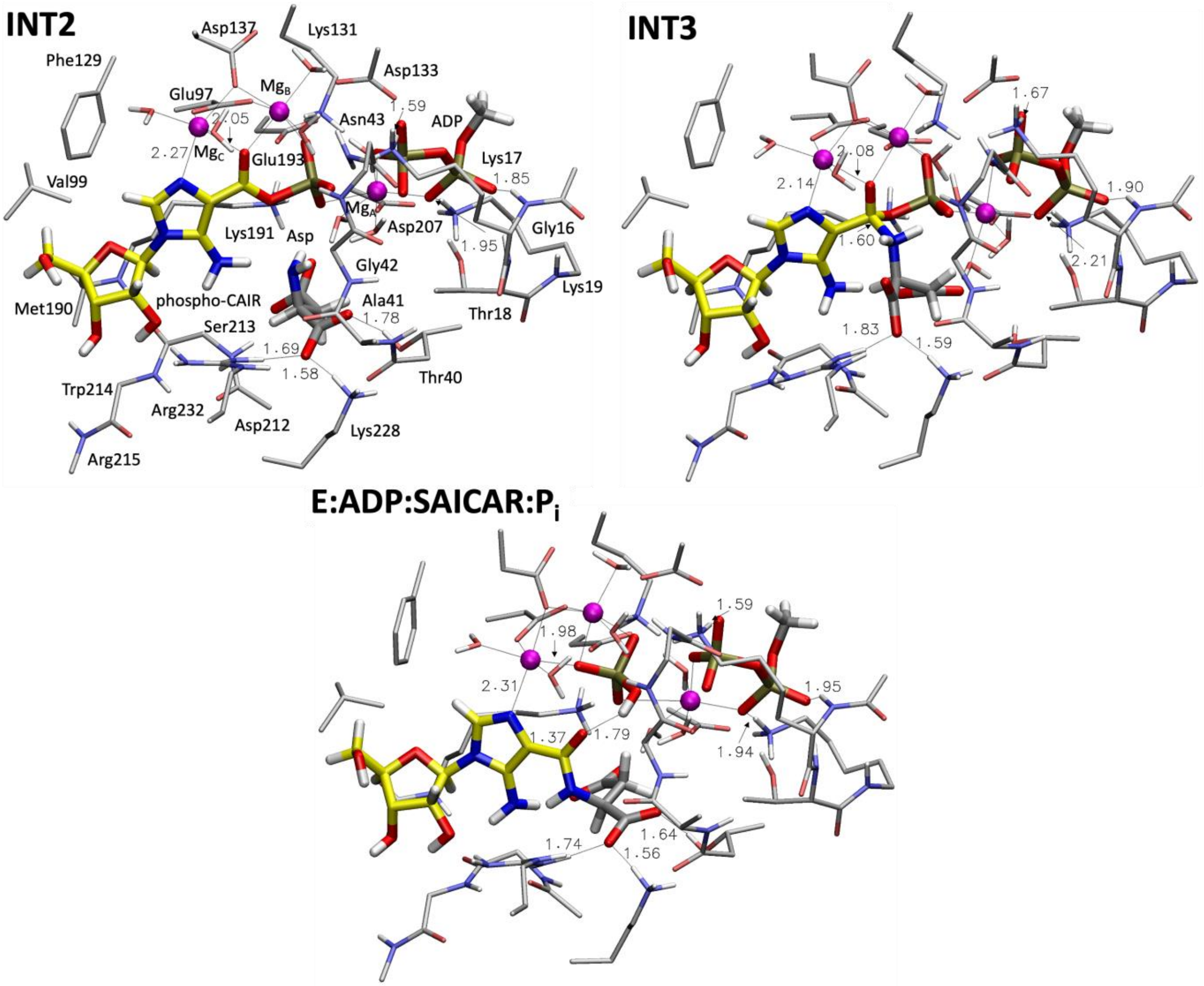

Figure S10. *Optimized structures of **INT2**, **INT3** and the **E:ADP:SAICAR:P**$_i$ complex. Selected distances are given in Å. For clarity, most of the hydrogens are omitted.*

*3.3 Superposition of **E:ADP:SAICAR:P$_i$** with the crystal structure.*

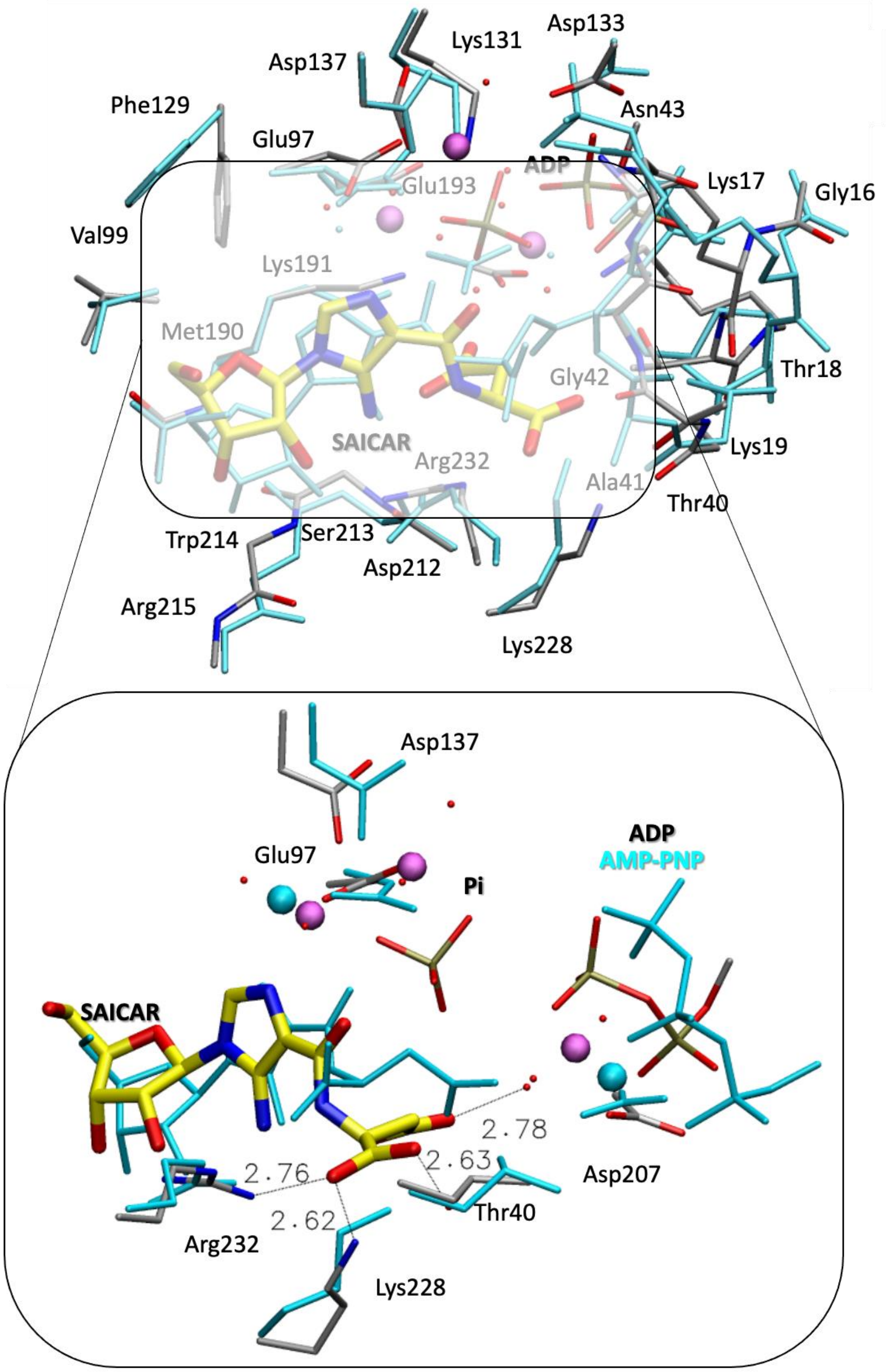

Figure S11. *Superposition of **E:ADP:SAICAR:P$_i$** and the crystal structure (PDB 6YB9, cyan).*

3.4 The alternative stepwise phosphorylation mechanism.

A stepwise γ-phosphate transfer from the **ATP** to the **CAIR** substrate has also been examined. According to this mechanistic proposal, Glu193 is first phosphorylated and then the γ-phosphate is transferred to Glu97, before the final transfer to **CAIR** to obtain **phospho-CAIR**. The calculations show that the first intermediate in this mechanism, *i.e.* the phosphorylated Glu193 (called **INT-phospho-Glu193**, Figure S12) has an energy of +16.3 kcal/mol relative to **E:ATP:CAIR:Asp**. This energy is 6.0 and 22.6 kcal/mol higher than the energies of **TS3** and **INT2**, respectively, that correspond to the S_N2 attack of the **CAIR** on the γ-phosphate of **ATP**.

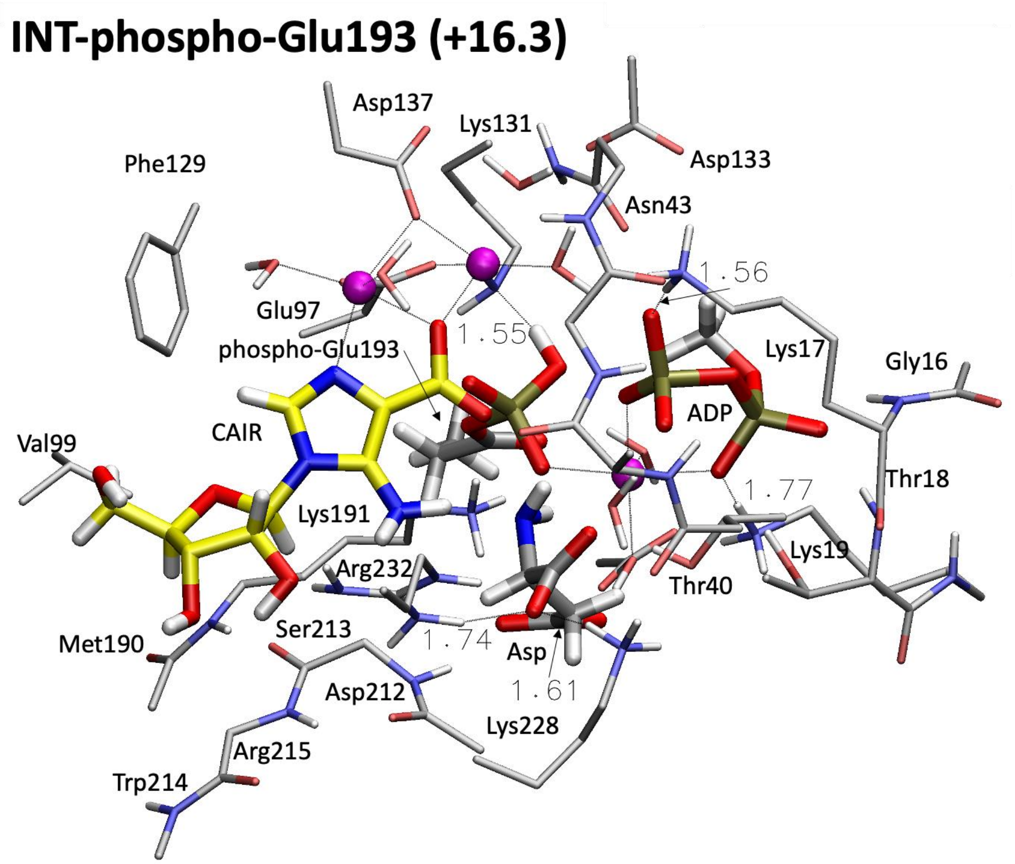

Figure S12. *Optimized structure of* ***INT-phospho-Glu193*** *in the alternative stepwise phosphorylation mechanism. Selected distances are given in Å. Energy (in kcal/mol) is given relative to* ***E:ATP:CAIR:Asp****. For clarity, most of the hydrogens are omitted.*

3.5. Results on the condensation-first mechanism.

We also investigated a mechanism in which the condensation reaction takes place before the phosphorylation (Scheme S1). In such a mechanism, the reaction to obtain **SAICAR** starts with the formation of the CAIR-Asp complex, corresponding to the geminal diolate on C^4 of **CAIR**. Next, the phosphorylation-dephosphorylation takes place by the transfer of γ-phosphate from the **ATP** to the CAIR-Asp complex and the dissociation of $\mathbf{P_i}$ and **SAICAR**.

Geometry optimization of the geminal diolate intermediate was not possible and resulted always in the return to the **E:ATP:CAIR:Asp** complex. Constrained optimization of a structure with the C^4_{CAIR}-N_{Asp} distance fixed to 1.50 Å (**INT-cond**, Figure S13) yields an energy of 31.5 kcal/mol relative to **E:ATP:CAIR:Asp**. This is more than 15 kcal/mol higher than the highest transition state (**TS5**), which rules out this mechanism possibility.

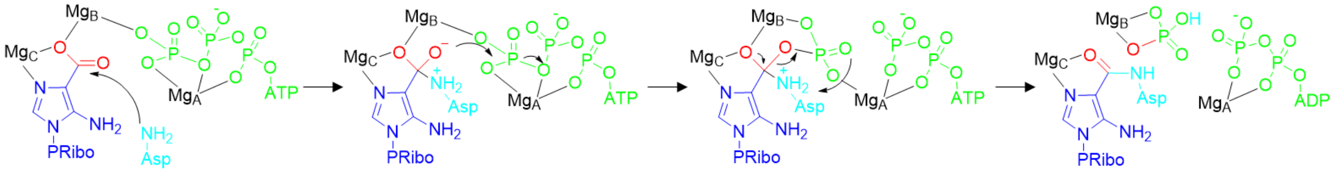

Scheme S1. *The alternative condensation-first mechanism.*

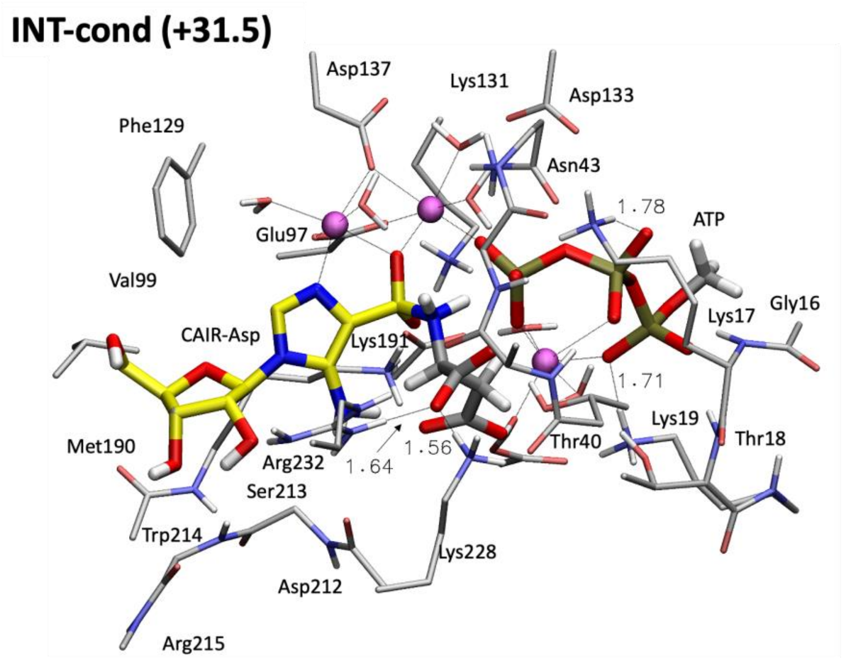

Figure S13. *Optimized structure of the constrained CAIR-Asp intermediate in the alternative condensation-first mechanism. Selected distances are given in Å. Energy (in kcal/mol) is given relative to* ***E:ATP:CAIR:Asp****. The* C_{CAIR}*-*N_{Asp} *distance was fixed to 1.50 Å. For clarity, most of the hydrogens are omitted.*

3.6. Metal-ligand bond distances.

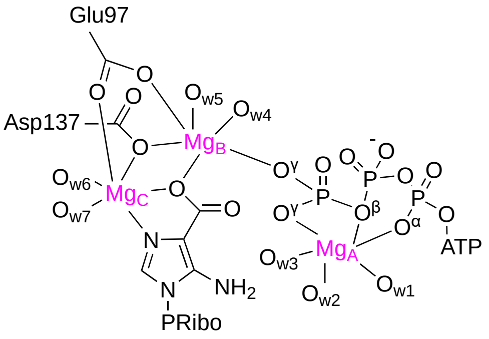

Table S1. *Metal-ligand bond distances (in Å) of Mg ions.*

Bond	E:ATP: CAIR:Asp	TS3	INT2	TS4	INT3	TS5	E:ADP: SAICAR:Pi	6YB9	2GQS
**Mg_A-O^{α}**	2.12	2.13	2.03	2.16	2.16	2.16	2.04	2.65	2.21
**Mg_A-O^{β}**	2.27	2.05	1.98	2.06	2.06	2.07	1.98	2.28	2.09
**Mg_A-O^{γ}**	2.09	2.15	3.45	2.18	2.14	2.10	3.95	3.25	-
**Mg_A-O_{w1}**	2.11	2.11	2.05	2.14	2.15	2.15	2.04	2.23	2.22
**Mg_A-O_{w2}**	2.04	2.06	2.07	2.12	2.12	2.13	2.09	3.03	2.10
**Mg_A-O_{w3}**	2.07	2.13	2.04	2.08	2.09	2.07	2.04	3.01	2.25
**Mg_B-O^{γ}**	2.01	2.00	1.99	1.98	1.99	1.98	2.05	-	-
**Mg_B-O_{CAIR}**	2.09	2.10	2.12	2.23	2.18	2.23	4.79	-	2.12
**Mg_B-O_{Glu97}**	2.17	2.18	2.11	2.11	2.10	2.09	2.10	-	2.17
**Mg_B-O_{Asp137}**	2.23	2.18	2.17	2.17	2.18	2.17	2.07	-	2.16
**Mg_B-O_{w4}**	2.11	2.08	2.06	2.08	2.09	2.09	2.06	-	2.01
**Mg_B-O_{w5}**	2.13	2.17	2.13	2.15	2.17	2.17	2.26	-	2.19
**Mg_C-O_{CAIR}**	2.04	2.06	2.05	2.13	2.07	2.08	3.54	3.00	2.07
**Mg_C-N_{CAIR}**	2.17	2.18	2.27	2.12	2.14	2.12	2.31	2.39	2.16
**Mg_C-O_{Glu97}**	2.02	2.02	2.00	2.05	2.04	2.04	2.08	2.15	2.06
**Mg_C-O_{Asp137}**	2.09	2.12	2.16	2.09	2.11	2.12	2.20	2.97	2.12
**Mg_C-O_{w6}**	2.20	2.19	2.20	2.20	2.20	2.19	2.09	3.52	2.13
**Mg_C-O_{w7}**	2.09	2.07	2.05	2.06	2.06	2.07	2.09	3.68	2.01

4. Absolute and relative energies.

***Table S2**. Calculated absolute (in a.u.) and relative (in kcal/mol) energies.*

BS1= LANL2DZ for Mg and 6-31G(d,p) for other atoms.

BS2= LANL2DZ for Mg and 6-311+G(2d,2p) for other atoms.

$E_{tot} = E_{BS2} + ZPE_{BS1} + (E_{solv,BS1} - E_{BS1})$.

The entropy contribution of the binding of CO_2 is included in the appropriate $\mathbf{E_{rel}}$.

Structure	E_{BS1}	ZPE_{BS1}	$E_{solv,BS1}$	E_{BS2}	E_{tot}	E_{rel}
**CO_2**	-	0.011646	-	-188.6521486	-188.6362332	**0.0**
**E:AIR**	-6907.652600	2.253598	-6907.779661	-6909.644709	-6907.518172	
**E:AIR:CO_2**	-7096.233302	2.265965	-7096.362985	-7098.288909	-7096.152626	**+14.9**
**TS1**	-7096.223491	2.265597	-7096.359502	-7098.277352	-7096.147766	**+17.9**
**INT1**	-7096.230680	2.267326	-7096.371651	-7098.286619	-7096.160265	**+10.1**
**TS2**	-7096.217736	2.263137	-7096.356416	-7098.272094	-7096.147637	**+18.0**
**E:CAIR**	-7096.233423	2.268484	-7096.373427	-7098.288826	-7096.160346	**+10.1**
**E:AIR:CO_2’**	-7096.256037	2.266840	-7096.378893	-7098.311169	-7096.167184	**+5.8**
**TS1’**	-7096.187442	2.266021	-7096.326738	-7098.242889	-7096.116164	**+37.8**
**INT1’**	-7096.214881	2.268731	-7096.352200	-7098.268940	-7096.137528	**+24.4**
**E:ylide**	-6907.586343	2.253331	-6907.71207	-6909.579861	-6907.452257	**+41.4**
**E:ATP:CAIR:Asp**	-9712.280180	3.108985	-9712.422822	-9715.254279	-9712.287936	**0.0**
**TS3**	-9712.265948	3.108599	-9712.409937	-9715.236149	-9712.271539	**+10.3**
**INT2**	-9712.296034	3.112990	-9712.439941	-9715.267081	-9712.297998	**-6.3**
**TS4**	-9712.251367	3.110260	-9712.406710	-9715.226867	-9712.271950	**+10.0**
**INT3**	-9712.255511	3.111894	-9712.412063	-9715.229396	-9712.274054	**+8.7**
**TS5**	-9712.235092	3.108642	-9712.392580	-9715.214481	-9712.263327	**+15.4**
**E:ADP:SAICAR:P_i**	-9712.293022	3.114152	-9712.437476	-9715.269023	-9712.299325	**-7.1**
**E:ATP:CAIR:Asp’**	-9712.289109	3.113025	-9712.422905	-9715.258171	-9712.278941	**+5.6**
**INT-phospho-Glu193**	-9712.245238	3.109897	-9712.397452	-9715.219659	-9712.261976	**+16.3**
**INT-cond**	-9712.234588	3.111900	-9712.379458	-9715.204845	-9712.237815	**+31.5**

5. Cartesian coordinates.

CO_2

C	-0.000011	0.000176	-0.000064
O	1.013995	-0.213803	-0.534818
O	-1.013987	0.213672	0.534866

E:AIR

C	2.438389	-1.965075	5.460668
C	1.121361	-2.216668	4.776139
O	0.277994	-3.000684	5.199449
N	0.969846	-1.554939	3.584995
C	-0.020171	-2.037331	2.657998
C	0.363688	-3.332023	1.943806
O	-0.474176	-3.923342	1.249871
N	1.635198	-3.763602	2.092447
C	2.119905	-4.910330	1.362924
C	3.424535	-5.419297	1.976408
O	4.069847	-4.788440	2.807751
C	2.361522	-4.515393	-0.104140
O	2.738910	-5.666936	-0.860437
N	3.826264	-6.618158	1.447083
C	5.207199	-7.041765	1.541464
C	6.170913	-6.493242	0.473947
O	7.353198	-6.821545	0.507609
N	5.655607	-5.605293	-0.419280
C	6.515130	-4.930123	-1.375607
C	7.288451	-3.724131	-0.790348
O	8.079430	-3.109360	-1.502490
C	5.728997	-4.513528	-2.623425
O	4.626368	-3.680620	-2.292545
N	7.058014	-3.455059	0.512946
C	7.630978	-2.321478	1.226187
C	6.527506	-1.717567	2.123126
C	5.314134	-1.211506	1.316745
O	5.550209	-0.249691	0.523079
O	4.196646	-1.776257	1.484556
C	-4.380401	-3.145300	3.053412
C	-5.838204	-2.757342	3.191774
O	-6.755824	-3.552327	2.945412
C	-4.089597	-3.394377	1.567907
O	-4.544615	-2.285987	0.790545
N	-6.057488	-1.455831	3.498027
C	-7.391711	-0.887928	3.535327
C	-7.985848	-0.579856	2.146662
O	-9.055432	0.027080	2.063439
C	-7.453494	0.345995	4.434201
N	-7.314992	-1.066067	1.074691
C	-7.934373	-1.137303	-0.233541
C	-8.383099	-2.559914	-0.624402
O	-8.898188	-2.739534	-1.729360
C	-6.996111	-0.612098	-1.326985
C	-6.672574	0.843351	-1.211013
N	-5.616013	1.373944	-1.932855
C	-7.289366	1.839119	-0.484515
C	-5.598526	2.664929	-1.655110
N	-6.591368	2.997935	-0.786323
N	-8.143343	-3.553337	0.265694
C	-8.212543	-4.948114	-0.150389
C	-6.840144	-5.463530	-0.647394
C	-6.239148	-4.577235	-1.751542
C	-4.977789	-5.111822	-2.461315
C	-3.640057	-4.706963	-1.823980
N	-2.461900	-5.051307	-2.651361
C	3.643731	2.684464	4.948747
C	2.947943	1.937832	3.826653
O	2.949530	0.706544	3.770712
C	5.074528	2.147263	5.156915
C	5.914031	2.237755	3.875340
C	5.749809	2.876043	6.321988
N	2.363241	2.701110	2.872708
C	1.693860	2.099953	1.725671
C	1.775287	3.136614	0.602808
O	1.258768	4.255857	0.737148
C	0.255787	1.703572	2.073688
N	2.482056	2.747585	-0.475181
C	2.693425	3.616094	-1.600607
C	1.546007	3.594722	-2.595818
O	0.391999	3.340602	-2.255422
N	1.845296	3.965583	-3.874801
C	0.782611	4.541506	-4.706102
C	0.121914	5.675723	-3.860259
O	0.818282	6.379869	-3.144624
C	-0.225198	3.513395	-5.246525
C	0.342733	2.189092	-5.761085
C	-0.805002	1.386068	-6.394322
N	-0.462865	0.023054	-6.787622
C	-0.415634	-1.032399	-5.937433
N	-0.807662	-0.892863	-4.675537
N	-0.002566	-2.221564	-6.377194
N	-1.241529	5.806770	-3.938190
C	-1.961341	6.241220	-2.735556
C	-2.257931	4.949810	-1.964160
O	-2.911370	4.053663	-2.516325
C	-3.264074	6.970416	-3.088177
O	-3.905550	7.454666	-1.933237
N	-1.753663	4.831663	-0.723623
C	-1.754746	3.553653	-0.045187
C	-3.065467	3.220759	0.650158
O	-3.828719	4.108679	1.083446
N	-3.277769	1.915467	0.865658
C	-4.437002	1.385616	1.589059
C	-4.271674	1.564189	3.106471
O	-4.056065	0.607066	3.856222
N	-4.381029	2.841160	3.550908
C	-3.875692	3.214974	4.873522
C	-2.341287	3.294620	4.828938
C	-1.630338	3.637915	6.145082
C	-1.909825	2.596397	7.236203
C	-0.120846	3.760851	5.890034
N	6.481889	4.868088	1.239441
C	7.588277	4.767061	0.278448
C	7.397184	5.705641	-0.925953
O	6.479614	5.548161	-1.715269
C	7.524861	3.284678	-0.130540
C	6.022779	2.915892	-0.051162
C	5.360868	4.086171	0.701388
P	0.896229	-4.041985	-3.450457
O	0.076871	-4.248090	-4.716704
O	0.019264	-4.288651	-2.150901
O	2.259937	-4.699380	-3.315952
O	1.119739	-2.397208	-3.294301
C	1.794174	-1.015774	-0.763127
O	1.915191	-0.794309	0.614386
C	3.082068	-0.741982	-1.569995
O	3.742434	0.421332	-1.111159
C	2.264686	-1.720806	-3.814749
C	2.521233	-0.479835	-2.964470
O	1.276702	0.233095	-2.738085
C	0.809050	-0.023975	-1.415205
N	-0.590686	-0.474273	-1.447789
C	-1.645310	0.290442	-1.943725
C	-2.801461	-0.299510	-1.497156
N	-2.474396	-1.408919	-0.728744
C	-1.159529	-1.494741	-0.713279
N	-1.466707	1.319699	-2.879248
O	-5.431642	5.679954	-0.391644
H	2.820074	-0.960481	5.272302
H	-0.199622	-1.285236	1.891018
H	-0.953129	-2.238376	3.188247
H	1.707505	-0.937141	3.261807
H	3.145992	-3.749591	-0.140717
H	1.422688	-4.115642	-0.481763
H	2.488416	-5.483929	-1.798037
H	1.353470	-5.691218	1.379120
H	2.338386	-3.185693	2.538021
H	5.597680	-6.704341	2.503857
H	3.313283	-6.903387	0.619161
H	5.383771	-5.416215	-3.142793
H	6.428725	-3.977389	-3.274469
H	3.808053	-4.069075	-2.666492
H	7.302716	-5.626120	-1.686274
H	4.646425	-5.516420	-0.544380
H	6.943557	-0.868920	2.678120
H	6.180289	-2.463546	2.844908
H	7.969659	-1.596243	0.483798
H	6.304079	-3.956329	0.962972
H	-4.625778	-4.298652	1.254654
H	-3.015362	-3.556436	1.421101
H	-3.784365	-2.001408	0.215488
H	-3.724333	-2.356986	3.434845
H	-6.798972	1.141329	4.066671
H	-8.475796	0.725234	4.441260
H	-7.145729	0.093177	5.451902
H	-8.057162	-1.663824	3.932328
H	-5.260921	-0.835790	3.642357
H	-7.464896	-0.826569	-2.291949
H	-6.061785	-1.185815	-1.300239
H	-8.111261	1.812501	0.214892
H	-6.708958	3.934451	-0.420243
H	-4.891865	3.385020	-2.038467
H	-8.838459	-0.524983	-0.190524
H	-6.386613	-1.473513	1.173018
H	-6.976274	-6.488102	-1.021065
H	-6.153463	-5.528427	0.205796
H	-6.002836	-3.599727	-1.318940
H	-7.016811	-4.394479	-2.498996
H	-4.974216	-4.721116	-3.488470
H	-5.024838	-6.206485	-2.557137
H	-3.503438	-5.176760	-0.845345
H	-3.613642	-3.627356	-1.653123
H	-2.553602	-4.653794	-3.586193
H	-0.960824	-4.558105	-2.292829
H	-2.409778	-6.059616	-2.787107
H	-8.950357	-5.007481	-0.953494
H	-7.685870	-3.353107	1.152112
H	4.971658	1.086824	5.414496
H	6.919168	1.840056	4.051553
H	5.472336	1.652545	3.063749
H	6.030030	3.271787	3.529122
H	5.873289	3.942742	6.097238
H	5.163575	2.793285	7.244063
H	6.744664	2.462630	6.516912
H	3.670211	3.762098	4.742643
H	-0.243556	1.243555	1.216934
H	0.275629	0.970530	2.882242
H	-0.318722	2.571996	2.405660
H	2.239356	1.196772	1.451936
H	2.293939	3.703258	2.977308
H	3.638953	3.354069	-2.081253
H	2.753368	4.656537	-1.257684
H	2.901566	1.800267	-0.548891
H	-0.784299	4.005529	-6.055048
H	-0.942205	3.271645	-4.460478
H	0.773194	1.626622	-4.927635
H	1.138934	2.349537	-6.497987
H	-1.167405	1.892935	-7.293743
H	-1.650881	1.354269	-5.699592

H	-0.285722	-0.163635	-7.761002
H	-1.049262	0.022464	-4.284346
H	-0.400454	-1.548710	-4.005496
H	0.433454	-2.285255	-7.282933
H	0.028700	-3.069876	-5.723126
H	1.291372	5.017506	-5.550662
H	2.764743	4.363838	-4.008213
H	-3.902947	6.289137	-3.669649
H	-3.011767	7.823969	-3.726294
H	-4.440531	6.740382	-1.531380
H	-1.307268	6.911335	-2.177889
H	-1.730395	5.050258	-4.398497
H	-1.471873	2.760137	-0.736035
H	-1.045311	5.490666	-0.432184
H	-4.498847	0.318335	1.378875
H	-5.346037	1.874618	1.232286
H	-2.721557	1.242244	0.346722
H	-2.062513	4.031898	4.063854
H	-1.974159	2.322721	4.477000
H	-1.998973	4.614176	6.495448
H	-1.354531	2.829660	8.150727
H	-1.604222	1.598172	6.902964
H	-2.971169	2.548376	7.497400
H	0.417689	4.028617	6.805186
H	0.095546	4.523071	5.133402
H	0.286164	2.809920	5.527076
H	-4.209622	2.453934	5.580240
H	-4.384733	3.553755	2.823532
H	8.098255	2.699088	0.592173
H	7.952665	3.107093	-1.120233
H	5.890592	1.960755	0.457034
H	5.595261	2.808807	-1.049227
H	4.746650	4.693470	0.019152
H	4.719432	3.756370	1.525119
H	8.526561	5.025427	0.785779
H	1.461800	-2.032623	-0.987323
H	2.809792	-1.153643	0.919639
H	3.761473	-1.599943	-1.554770
H	4.462566	0.139733	-0.427377
H	2.067654	-1.426944	-4.852796
H	3.144165	-2.369686	-3.795193
H	3.192384	0.194922	-3.504500
H	0.817399	0.924141	-0.874382
H	-0.598950	-2.267688	-0.212670
H	-0.636749	1.886416	-2.700828
H	-4.748716	5.176426	0.113374
H	-5.721527	6.372225	0.216804
H	-3.817744	0.000862	-1.720139
C	6.133415	6.202085	1.681088
H	5.392795	6.133169	2.484922
H	7.018237	6.705837	2.086107
H	5.699740	6.838615	0.887246
C	8.359991	6.874807	-1.055854
H	8.121265	7.462327	-1.943305
H	8.295694	7.507458	-0.162706
H	9.392783	6.511150	-1.111367
H	-4.322579	4.172730	5.156880
H	3.058164	2.540242	5.864674
H	8.489440	-2.635423	1.833284
H	2.333361	-2.153117	6.530682
H	3.165144	-2.680742	5.056112
H	-0.976140	3.609721	0.716175
H	-4.195023	-4.062281	3.620474
H	-8.559179	-5.551382	0.693802
H	5.272949	-8.133004	1.521150
H	-2.268995	1.948068	-2.865938

E:AIR:CO_2

C	2.828035	-1.753310	5.266913
C	1.454364	-1.881100	4.657709
O	0.602460	-2.661078	5.073515
N	1.254629	-1.134784	3.520021
C	0.339863	-1.667445	2.534925
C	0.773350	-3.045281	2.021599
O	-0.044810	-3.845296	1.561693
N	2.104871	-3.303274	2.079932
C	2.639486	-4.554213	1.602604
C	3.998504	-4.836149	2.242738
O	4.632566	-3.997460	2.878753
C	2.796793	-4.485652	0.075441
O	3.230437	-5.749331	-0.42832
N	4.469441	-6.091696	1.967313
C	5.884944	-6.380080	2.069429
C	6.763087	-5.923924	0.889827
O	7.978458	-6.084987	0.947381
N	6.127826	-5.302064	-0.140422
C	6.880382	-4.732706	-1.243469
C	7.495423	-3.342644	-0.94866
O	8.188087	-2.799260	-1.806722
C	6.038806	-4.704968	-2.524143
O	4.818668	-4.000173	-2.332992
N	7.262590	-2.837664	0.281997
C	7.790615	-1.563258	0.754976
C	6.718604	-0.887847	1.634132
C	5.433746	-0.542830	0.85672
O	5.561831	0.275991	-0.097175
O	4.351696	-1.098080	1.222984
C	-4.026037	-3.258179	3.159771
C	-5.474827	-2.841399	3.287272
O	-6.402432	-3.649590	3.175554
C	-3.796530	-3.872438	1.773304
O	-4.328933	-3.026735	0.757044
N	-5.689769	-1.508796	3.477419
C	-7.028207	-0.945502	3.460101
C	-7.545826	-0.638488	2.042653
O	-8.569592	0.030074	1.893403
C	-7.150935	0.260855	4.386003
N	-6.881506	-1.227803	1.01582
C	-7.483841	-1.352697	-0.296898
C	-7.940137	-2.794681	-0.616012
O	-8.424002	-3.040555	-1.721532
C	-6.542039	-0.875319	-1.409536
C	-6.203922	0.586146	-1.364595
N	-5.222557	1.111209	-2.20005
C	-6.785715	1.601823	-0.637546
C	-5.228526	2.413971	-1.985001
N	-6.155508	2.763305	-1.052369
N	-7.766489	-3.726558	0.353823
C	-7.909173	-5.149702	0.070439
C	-6.570954	-5.786378	-0.36698
C	-5.983530	-5.148397	-1.634604
C	-4.711438	-5.810600	-2.197703
C	-3.405390	-5.414869	-1.495172
N	-2.183629	-5.901611	-2.173669
C	3.709563	2.984440	4.935735
C	2.996711	2.223362	3.83323
O	3.183745	1.015528	3.674716
C	5.189654	2.570164	5.040327
C	5.946435	2.810201	3.727581
C	5.861105	3.291707	6.212341
N	2.148114	2.940918	3.054358
C	1.496599	2.353910	1.893643
C	1.772665	3.262204	0.695403
O	1.551842	4.477785	0.760411
C	-0.011255	2.180238	2.100366
N	2.264064	2.624548	-0.383341
C	2.503877	3.289478	-1.633585
C	1.275041	3.303892	-2.538901
O	0.119477	3.249358	-2.113102
N	1.552051	3.428062	-3.863614
C	0.520670	3.775730	-4.835589
C	-0.234277	5.023778	-4.30973
O	0.380094	5.974677	-3.838908
C	-0.400692	2.602709	-5.197122
C	0.294553	1.318344	-5.646633
C	-0.786843	0.274695	-5.944695
N	-0.265863	-1.024220	-6.36743
C	-0.404951	-2.183015	-5.694867
N	-0.869667	-2.190042	-4.439329
N	-0.110777	-3.349449	-6.274391
N	-1.593408	5.005940	-4.432341
C	-2.397661	5.952816	-3.662172
C	-2.357826	5.502744	-2.201631
O	-3.122425	4.623386	-1.791623
C	-3.844848	5.958189	-4.172779
O	-4.665106	6.796372	-3.39829
N	-1.447706	6.094162	-1.403268
C	-1.299735	5.604277	-0.044216
C	-2.681897	5.556853	0.610831
O	-3.476823	6.498608	0.530632
N	-2.950705	4.423568	1.312958
C	-4.331772	4.113183	1.666006
C	-4.422782	3.250834	2.916669
O	-4.909360	2.119920	2.874368
N	-3.961139	3.819198	4.05898
C	-3.820937	3.025419	5.279887
C	-2.677463	2.005839	5.141305
C	-2.480990	1.035611	6.316295
C	-3.730950	0.183441	6.577268
C	-1.268073	0.135354	6.041893
N	6.175008	5.526131	1.18879
C	7.231345	5.538593	0.169957
C	6.926869	6.531599	-0.967927
O	5.975860	6.369990	-1.714597
C	7.217235	4.078036	-0.30962
C	5.724452	3.680528	-0.269663
C	5.064825	4.702885	0.681351
P	1.086584	-4.778782	-2.982134
O	0.148877	-4.919514	-4.185505
O	0.289308	-5.098098	-1.647527
O	2.443558	-5.464033	-2.994891
O	1.363062	-3.161026	-2.758466
C	1.884763	-0.928603	-0.869935
O	2.120074	-0.234657	0.326965
C	3.063405	-0.832873	-1.867523
O	3.641873	0.452384	-1.836036
C	2.251997	-2.489734	-3.649852
C	2.369284	-1.040106	-3.209546
O	1.050381	-0.446448	-3.057204
C	0.736627	-0.279511	-1.663377
N	-0.594114	-0.805225	-1.412316
C	-1.770744	-0.167831	-1.813628
C	-2.789879	-1.023103	-1.491195
N	-2.275554	-2.162637	-0.893978
C	-0.968669	-2.021545	-0.862468
N	-1.812999	1.042380	-2.504296
C	-3.258108	0.668575	1.060793
O	-3.027350	1.629959	0.433986
O	-3.444644	-0.283277	1.707771
O	-5.726873	5.756342	-0.94126
H	3.246935	-0.753727	5.137592
H	0.300374	-0.983628	1.685335
H	-0.662088	-1.792891	2.948477
H	2.004014	-0.518661	3.218451
H	3.518499	-3.698870	-0.176194
H	1.819083	-4.233490	-0.326895
H	2.895429	-5.825367	-1.355038
H	1.925967	-5.351042	1.832516
H	2.781236	-2.591680	2.335093
H	6.275955	-5.868707	2.950968
H	3.948815	-6.585765	1.249389
H	5.840191	-5.737256	-2.837792
H	6.644909	-4.212664	-3.29275
H	4.065748	-4.570819	-2.587404
H	7.754752	-5.367447	-1.428503

H	5.115033	-5.371443	-0.247097
H	7.127799	0.046839	2.035069
H	6.456595	-1.535130	2.476743
H	8.016215	-0.950233	-0.11949
H	6.614598	-3.330972	0.881034
H	-4.309674	-4.840784	1.733541
H	-2.723124	-4.033508	1.622563
H	-3.570354	-2.745971	0.189891
H	-3.353450	-2.409606	3.311599
H	-6.434462	1.035155	4.103306
H	-8.158023	0.671288	4.299205
H	-6.965974	-0.040290	5.420873
H	-7.687442	-1.749521	3.806246
H	-4.911770	-0.872511	3.36788
H	-7.014609	-1.123458	-2.364613
H	-5.627588	-1.476995	-1.360619
H	-7.553770	1.588108	0.121043
H	-6.324530	3.724204	-0.771982
H	-4.569103	3.147857	-2.421497
H	-8.386091	-0.735801	-0.28494
H	-5.987708	-1.691735	1.160693
H	-6.741985	-6.859492	-0.534756
H	-5.858428	-5.704737	0.46188
H	-5.760224	-4.098400	-1.424987
H	-6.756996	-5.144913	-2.409652
H	-4.615285	-5.518595	-3.25317
H	-4.818339	-6.905923	-2.195937
H	-3.380019	-5.787627	-0.46688
H	-3.324431	-4.328288	-1.430872
H	-2.168326	-5.603043	-3.149148
H	-0.690962	-5.373335	-1.771801
H	-2.170070	-6.919907	-2.196022
H	-8.654435	-5.249126	-0.722416
H	-7.306153	-3.476136	1.222584
H	5.196576	1.492150	5.236889
H	6.987236	2.483060	3.822912
H	5.501421	2.244026	2.904688
H	5.963129	3.869655	3.445822
H	5.873793	4.376562	6.049243
H	5.339154	3.100739	7.156735
H	6.899036	2.963693	6.329147
H	3.627978	4.067822	4.7767
H	-0.461665	1.754197	1.198838
H	-0.193866	1.501840	2.937808
H	-0.485475	3.144219	2.310776
H	1.945300	1.378489	1.730949
H	2.125430	3.949686	3.116199
H	3.328598	2.781173	-2.134521
H	2.768827	4.335982	-1.44572
H	2.469539	1.622708	-0.328582
H	-1.074394	2.944100	-5.994581
H	-1.006036	2.342219	-4.324449
H	0.934643	0.947696	-4.844074
H	0.914823	1.494072	-6.536953
H	-1.458646	0.640863	-6.731885
H	-1.395181	0.124852	-5.050912
H	0.196254	-1.072064	-7.262746
H	-0.535317	-1.440514	-3.834673
H	-0.820095	-3.112872	-4.006082
H	0.141795	-3.388972	-7.248038
H	0.074577	-4.148232	-5.613447
H	1.052872	4.105186	-5.734999
H	2.515048	3.577644	-4.123877
H	-4.216254	4.922556	-4.193608
H	-3.844558	6.331892	-5.202718
H	-4.974206	6.299830	-2.614362
H	-1.956259	6.944296	-3.771039
H	-2.021801	4.098332	-4.548351
H	-0.811135	4.626727	-0.035785
H	-0.681748	6.586393	-1.8453
H	-4.814986	3.560733	0.860266
H	-4.850790	5.061893	1.816762
H	-2.396744	3.615475	1.058246
H	-1.743254	2.555202	4.96595
H	-2.868937	1.417035	4.237048
H	-2.275046	1.628099	7.22142
H	-3.547473	-0.533842	7.383446
H	-4.007093	-0.389489	5.684769
H	-4.598170	0.787146	6.862801
H	-1.058422	-0.527712	6.885981
H	-0.364470	0.720344	5.840587
H	-1.438255	-0.506885	5.17147
H	-4.772415	2.518491	5.449652
H	-3.401972	4.654894	3.957816
H	7.793316	3.477761	0.399433
H	7.662782	3.951753	-1.299651
H	5.598775	2.644615	0.051417
H	5.292275	3.777875	-1.265793
H	4.319803	5.312061	0.148352
H	4.552293	4.231876	1.526708
H	8.181550	5.818589	0.642931
H	1.665857	-1.983057	-0.690567
H	3.030155	-0.556528	0.68704
H	3.811119	-1.610698	-1.679198
H	4.391058	0.417974	-1.157107
H	1.867753	-2.541329	-4.674811
H	3.246622	-2.944527	-3.634692
H	2.900858	-0.472184	-3.979696
H	0.700433	0.789465	-1.447947
H	-0.280591	-2.738353	-0.449389
H	-1.233724	1.782825	-2.109575
H	-4.925167	5.999417	-0.42534
H	-6.363219	6.456260	-0.745447
H	-3.837054	-0.868692	-1.677182
C	5.789497	6.812031	1.730494
H	5.102809	6.658657	2.569877
H	6.672335	7.335841	2.114069
H	5.278343	7.470317	1.003514
C	7.814742	7.761087	-1.079963
H	7.494225	8.379133	-1.919526
H	7.762135	8.340331	-0.150454
H	8.862461	7.464565	-1.208178
H	-3.649178	3.711228	6.114783
H	3.196609	2.757187	5.878733
H	8.714478	-1.716932	1.327251
H	2.789604	-2.030097	6.32202
H	3.483613	-2.460236	4.743019
H	-0.659279	6.293398	0.50986
H	-3.805695	-4.005718	3.929182
H	-8.283550	-5.644879	0.971336
H	6.040457	-7.452961	2.211003
H	-2.784780	1.345078	-2.532042

TS1

C	2.892164	-1.743706	5.251298
C	1.507898	-1.909778	4.676850
O	0.693356	-2.721541	5.106217
N	1.258096	-1.160436	3.552573
C	0.302259	-1.685522	2.604656
C	0.720803	-3.047361	2.041870
O	-0.106164	-3.818039	1.547113
N	2.047400	-3.328022	2.099519
C	2.571259	-4.576426	1.607999
C	3.931918	-4.871501	2.239502
O	4.569071	-4.043496	2.885194
C	2.730637	-4.489158	0.083127
O	3.132804	-5.755340	-0.441516
N	4.402417	-6.121870	1.938685
C	5.819599	-6.409383	2.018376
C	6.678483	-5.953895	0.824173
O	7.893502	-6.122237	0.857334
N	6.027894	-5.322808	-0.191864
C	6.762600	-4.764742	-1.312579
C	7.402886	-3.382554	-1.035965
O	8.076211	-2.847466	-1.914412
C	5.897259	-4.729946	-2.577177
O	4.687941	-4.007728	-2.371260
N	7.215407	-2.876008	0.201643
C	7.781595	-1.611843	0.658210
C	6.757001	-0.920402	1.576974
C	5.447676	-0.589598	0.841106
O	5.539000	0.240356	-0.109724
O	4.388464	-1.172128	1.223253
C	-3.999158	-3.249220	3.269884
C	-5.363615	-2.590283	3.295801
O	-6.418258	-3.230462	3.315100
C	-3.849915	-3.983235	1.926130
O	-4.329699	-3.189529	0.843718
N	-5.329445	-1.233155	3.210628
C	-6.523983	-0.419070	3.091757
C	-7.204952	-0.461208	1.716052
O	-8.218108	0.217560	1.536018
C	-6.214381	1.030181	3.453614
N	-6.650556	-1.244163	0.759616
C	-7.342508	-1.458722	-0.498782
C	-7.890688	-2.890884	-0.650571
O	-8.415446	-3.229784	-1.711953
C	-6.469172	-1.123893	-1.715892
C	-6.047847	0.313753	-1.817291
N	-5.178174	0.702534	-2.826839
C	-6.380860	1.407645	-1.042788
C	-5.003611	2.001490	-2.662553
N	-5.706525	2.478361	-1.597414
N	-7.743774	-3.723242	0.410859
C	-7.936850	-5.159545	0.262271
C	-6.629668	-5.867924	-0.160941
C	-6.075065	-5.364666	-1.501178
C	-4.780612	-6.037388	-1.992427
C	-3.491623	-5.504199	-1.352319
N	-2.254597	-6.010629	-1.985530
C	3.781109	2.988036	4.859666
C	3.045352	2.253082	3.752411
O	3.170084	1.035430	3.609184
C	5.242253	2.512922	4.974787
C	6.016848	2.719030	3.666602
C	5.935072	3.208601	6.149973
N	2.254076	3.004203	2.944052
C	1.567073	2.427389	1.796505
C	1.851168	3.316605	0.584132
O	1.560256	4.518007	0.587275
C	0.061253	2.277704	2.033744
N	2.454206	2.678947	-0.441773
C	2.709799	3.311130	-1.707300
C	1.498514	3.256936	-2.626562
O	0.350914	3.132443	-2.196761
N	1.739489	3.398020	-3.956846
C	0.647008	3.763150	-4.855958
C	-0.058678	5.016842	-4.263989
O	0.580307	5.871470	-3.665147
C	-0.334068	2.613114	-5.140092
C	0.274697	1.283059	-5.576566
C	-0.873571	0.310289	-5.870540
N	-0.441180	-1.021576	-6.284798
C	-0.612522	-2.156048	-5.578383
N	-1.014800	-2.104707	-4.299736
N	-0.408653	-3.347290	-6.140207
N	-1.408464	5.097461	-4.459148
C	-2.213032	5.933040	-3.566503
C	-2.332406	5.181998	-2.238174
O	-3.187686	4.298949	-2.091202
C	-3.605195	6.157851	-4.172342
O	-4.437731	6.897700	-3.317215
N	-1.460549	5.494834	-1.266855
C	-1.608058	4.862758	0.023661
C	-2.955103	5.274358	0.628864

O	-3.459665	6.376540	0.386190
N	-3.536454	4.368502	1.451816
C	-4.838325	4.636377	2.046282
C	-4.978337	4.169820	3.496030
O	-6.087724	4.079376	4.008702
N	-3.812169	3.879494	4.126831
C	-3.742716	3.054031	5.324959
C	-2.855900	1.836315	5.026078
C	-2.640333	0.836238	6.171247
C	-3.952313	0.176931	6.616387
C	-1.623513	-0.227735	5.735688
N	6.261343	5.488980	1.095378
C	7.319667	5.506303	0.078649
C	6.972723	6.431164	-1.103654
O	6.034075	6.191181	-1.845400
C	7.372958	4.027249	-0.334845
C	5.896192	3.575453	-0.297624
C	5.189356	4.601519	0.614660
P	0.891974	-4.744084	-2.895285
O	-0.098671	-4.889794	-4.055590
O	0.139670	-5.024314	-1.526934
O	2.239924	-5.440332	-2.965113
O	1.187700	-3.126160	-2.691254
C	1.846782	-0.928640	-0.766515
O	2.140691	-0.268696	0.430293
C	2.985404	-0.855439	-1.810517
O	3.600117	0.412163	-1.792103
C	2.076495	-2.466341	-3.589416
C	2.233446	-1.027300	-3.127635
O	0.920769	-0.423387	-2.910600
C	0.705682	-0.211745	-1.505621
N	-0.642895	-0.624681	-1.155661
C	-1.763047	0.182059	-1.262997
C	-2.837450	-0.574401	-0.753600
N	-2.370165	-1.871550	-0.493204
C	-1.087962	-1.873734	-0.699802
N	-1.769303	1.421471	-1.775653
C	-2.979800	0.494166	1.106980
O	-3.331073	1.596667	0.777400
O	-2.718859	-0.284349	1.976405
O	-5.644839	5.519013	-1.106742
H	3.289464	-0.739482	5.092772
H	0.204930	-0.983320	1.776366
H	-0.676078	-1.824582	3.063087
H	1.975987	-0.518356	3.230183
H	3.471085	-3.716529	-0.157198
H	1.759436	-4.206466	-0.312144
H	2.759288	-5.825286	-1.353077
H	1.853628	-5.372948	1.827156
H	2.727772	-2.641163	2.402703
H	6.223163	-5.896927	2.893648
H	3.872439	-6.609019	1.223247
H	5.679789	-5.760089	-2.884564
H	6.494993	-4.246724	-3.357542
H	3.924779	-4.577374	-2.588760
H	7.625133	-5.410588	-1.515195
H	5.014022	-5.393594	-0.282603
H	7.187134	0.017487	1.946170
H	6.527344	-1.556072	2.437593
H	7.984713	-1.000576	-0.222993
H	6.575558	-3.358130	0.818233
H	-4.451934	-4.897623	1.959015
H	-2.798353	-4.251116	1.773412
H	-3.549969	-2.782365	0.388274
H	-3.200645	-2.511974	3.383549
H	-5.426090	1.424362	2.802367
H	-7.098755	1.649775	3.319275
H	-5.880319	1.108116	4.488353
H	-7.270487	-0.827540	3.780406
H	-4.432147	-0.796725	3.041543
H	-7.031921	-1.417123	-2.606605
H	-5.575190	-1.763099	-1.703667
H	-7.024736	1.505108	-0.181664
H	-5.703193	3.449150	-1.290308
H	-4.378033	2.648296	-3.256726
H	-8.207934	-0.790526	-0.482351
H	-5.805208	-1.785661	0.944348
H	-6.825629	-6.948059	-0.221092
H	-5.884364	-5.724359	0.629341
H	-5.888321	-4.290557	-1.422998
H	-6.852027	-5.478777	-2.264048
H	-4.700137	-5.873642	-3.076351
H	-4.836407	-7.127543	-1.854203
H	-3.443715	-5.754641	-0.289559
H	-3.467190	-4.413106	-1.407779
H	-2.242133	-5.798414	-2.983715
H	-0.818696	-5.391157	-1.622606
H	-2.209175	-7.025477	-1.911688
H	-8.712071	-5.308013	-0.493159
H	-7.271994	-3.397971	1.248023
H	5.203609	1.435963	5.173440
H	7.043020	2.350694	3.769943
H	5.554924	2.169274	2.841779
H	6.076893	3.776839	3.383897
H	5.993561	4.291628	5.985195
H	5.399234	3.041025	7.090892
H	6.957684	2.838060	6.274413
H	3.745890	4.073435	4.697851
H	-0.423066	1.873634	1.140118
H	-0.116539	1.586486	2.861207
H	-0.395973	3.244356	2.265732
H	1.991778	1.440738	1.634840
H	2.240420	4.010801	3.029778
H	3.559240	2.815813	-2.177748
H	2.938341	4.371701	-1.552836
H	2.675170	1.683786	-0.375090
H	-1.015576	2.955515	-5.930788
H	-0.928662	2.430116	-4.240567
H	0.896927	0.881800	-4.775596
H	0.900164	1.410870	-6.470363
H	-1.516192	0.716304	-6.661761
H	-1.504757	0.195055	-4.986795
H	-0.020910	-1.113386	-7.197098
H	-0.581492	-1.381642	-3.721331
H	-1.013092	-3.023588	-3.856474
H	-0.227113	-3.424519	-7.127607
H	-0.227139	-4.141445	-5.468164
H	1.116238	4.070916	-5.796846
H	2.671993	3.678704	-4.223949
H	-4.039243	5.178066	-4.423492
H	-3.484420	6.719263	-5.105406
H	-4.834785	6.293538	-2.659158
H	-1.698870	6.884722	-3.431522
H	-1.876067	4.245164	-4.731584
H	-1.553794	3.775034	-0.069209
H	-0.667866	6.087120	-1.466121
H	-5.640045	4.149042	1.482711
H	-5.008334	5.713740	1.998185
H	-3.283333	3.389347	1.333932
H	-1.871629	2.205607	4.702968
H	-3.272154	1.306187	4.161308
H	-2.223089	1.386079	7.028874
H	-3.773092	-0.526544	7.435839
H	-4.399657	-0.386272	5.789542
H	-4.689337	0.908793	6.963118
H	-1.413297	-0.945840	6.532686
H	-0.671390	0.215894	5.428313
H	-2.002607	-0.795274	4.879201
H	-4.767961	2.780226	5.577528
H	-2.970580	3.961637	3.573227
H	7.961734	3.484747	0.409745
H	7.838482	3.874596	-1.312009
H	5.798475	2.544938	0.048644
H	5.475566	3.631028	-1.301525
H	4.422638	5.161330	0.058763
H	4.693651	4.138459	1.474085
H	8.254071	5.851368	0.539653
H	1.587993	-1.978115	-0.604910
H	3.054605	-0.614128	0.755058
H	3.717456	-1.656539	-1.662867
H	4.372103	0.359048	-1.131920
H	1.674507	-2.496085	-4.608882
H	3.060714	-2.943158	-3.598612
H	2.740078	-0.447660	-3.905323
H	0.769194	0.856783	-1.297659
H	-0.427343	-2.710837	-0.533863
H	-0.918622	1.974485	-1.860574
H	-4.922737	5.895483	-0.555754
H	-6.435014	6.017601	-0.862379
H	-3.875306	-0.388519	-0.983862
C	5.819384	6.778203	1.584032
H	5.135227	6.629707	2.426230
H	6.677412	7.353402	1.949643
H	5.284690	7.384622	0.829652
C	7.801401	7.694243	-1.267931
H	7.457035	8.258623	-2.135365
H	7.715964	8.311300	-0.365595
H	8.862780	7.442577	-1.378588
H	-3.336415	3.628342	6.167943
H	3.252738	2.783300	5.799025
H	8.724360	-1.782834	1.193523
H	2.882956	-1.998845	6.312635
H	3.548345	-2.451623	4.729641
H	-0.779043	5.174987	0.662203
H	-3.926552	-3.965237	4.093924
H	-8.291115	-5.562656	1.215468
H	5.979541	-7.481831	2.158317
H	-2.627475	1.951684	-1.676659

INT1

C	2.790838	-1.934391	5.217731
C	1.433516	-2.236156	4.630010
O	0.781445	-3.237557	4.913675
N	1.042587	-1.371463	3.646005
C	0.050791	-1.801024	2.694522
C	0.467454	-3.086981	1.975163
O	-0.362219	-3.826221	1.440839
N	1.803161	-3.330802	1.940175
C	2.318637	-4.591648	1.477566
C	3.658773	-4.908095	2.137801
O	4.328504	-4.070668	2.737821
C	2.527317	-4.522515	-0.038288
O	2.891136	-5.807649	-0.542733
N	4.080705	-6.188238	1.901804
C	5.481472	-6.534989	2.022086
C	6.388648	-6.139368	0.842882
O	7.594348	-6.356834	0.910358
N	5.790773	-5.497781	-0.198356
C	6.577114	-4.977904	-1.301551
C	7.278704	-3.628348	-1.012323
O	7.993044	-3.130854	-1.880253
C	5.746172	-4.904892	-2.587448
O	4.561652	-4.132388	-2.413696
N	7.097617	-3.109647	0.221468
C	7.736659	-1.882328	0.683727
C	6.747356	-1.106054	1.564660
C	5.472144	-0.667089	0.821030
O	5.642448	-0.048491	-0.270301
O	4.361764	-0.935485	1.368539
C	-4.106727	-3.281905	3.146297
C	-5.474867	-2.629740	3.189879
O	-6.523877	-3.284104	3.134090
C	-3.975019	-4.033812	1.816151
O	-4.365307	-3.212253	0.714457
N	-5.447019	-1.274458	3.199856

C	-6.643544	-0.471655	3.046211
C	-7.173321	-0.403445	1.601685
O	-8.110987	0.351512	1.333616
C	-6.416480	0.937939	3.588207
N	-6.588004	-1.219049	0.691603
C	-7.164714	-1.418871	-0.622847
C	-7.787130	-2.819344	-0.812622
O	-8.281656	-3.111954	-1.902703
C	-6.139928	-1.170594	-1.741647
C	-5.650059	0.245800	-1.835563
N	-4.639366	0.578840	-2.731766
C	-6.060710	1.376733	-1.157256
C	-4.467903	1.883094	-2.598526
N	-5.294657	2.413557	-1.655329
N	-7.733140	-3.674438	0.238480
C	-8.047820	-5.091739	0.081555
C	-6.800824	-5.906448	-0.322121
C	-6.256894	-5.526747	-1.705662
C	-4.948770	-6.213531	-2.126973
C	-3.690716	-5.664900	-1.439920
N	-2.428153	-6.096306	-2.077727
C	3.784844	2.779332	4.863223
C	2.781984	2.252916	3.863031
O	2.351514	1.102569	3.954339
C	5.079480	1.940387	4.851390
C	5.742547	1.945861	3.469685
C	6.039340	2.438541	5.935492
N	2.416758	3.083785	2.856783
C	1.481274	2.655658	1.820099
C	1.824128	3.447168	0.554286
O	1.622756	4.661758	0.480546
C	0.027828	2.865250	2.248703
N	2.382270	2.714605	-0.438832
C	2.729748	3.290219	-1.710276
C	1.561475	3.300344	-2.686446
O	0.382658	3.195857	-2.335704
N	1.864910	3.465026	-3.997337
C	0.803040	3.812968	-4.941705
C	0.070044	5.072255	-4.394292
O	0.702733	5.988896	-3.890746
C	-0.154040	2.650319	-5.247778
C	0.475161	1.319945	-5.653853
C	-0.655221	0.379721	-6.089455
N	-0.224694	-0.971397	-6.431648
C	-0.512627	-2.082770	-5.724925
N	-1.030839	-1.992593	-4.490786
N	-0.309446	-3.291391	-6.246396
N	-1.289232	5.083504	-4.530247
C	-2.095758	5.960105	-3.683956
C	-2.111963	5.355468	-2.277630
O	-2.782078	4.340806	-2.039734
C	-3.524402	6.036112	-4.245296
O	-4.356494	6.845316	-3.458962
N	-1.360276	5.949477	-1.340075
C	-1.444927	5.493542	0.030698
C	-2.894861	5.615033	0.515330
O	-3.598186	6.572378	0.153513
N	-3.320712	4.648445	1.338588
C	-4.656644	4.654189	1.924232
C	-4.613220	4.471436	3.448811
O	-5.154723	5.267089	4.210416
N	-3.926097	3.377552	3.843286
C	-3.740932	3.003326	5.239573
C	-2.563124	2.019898	5.282946
C	-2.332697	1.253181	6.591482
C	-3.517327	0.336471	6.928331
C	-1.038903	0.433590	6.470794
N	6.348714	5.245395	1.131575
C	7.413915	5.243170	0.121535
C	7.105076	6.195944	-1.048821
O	6.163453	5.997603	-1.799359
C	7.422309	3.769827	-0.314904
C	5.931001	3.367603	-0.296890
C	5.252660	4.400109	0.629529
P	0.645682	-4.681580	-2.923548
O	-0.346065	-4.757615	-4.093631
O	-0.122863	-4.971652	-1.569164
O	1.969246	-5.416784	-3.034354
O	0.989933	-3.079856	-2.660071
C	1.767256	-0.836175	-0.663164
O	2.077218	-0.162677	0.520526
C	2.898827	-0.844494	-1.713542
O	3.580056	0.386360	-1.720601
C	1.951869	-2.444419	-3.498025
C	2.128354	-1.010494	-3.026261
O	0.816667	-0.393630	-2.809135
C	0.671459	-0.071579	-1.424283
N	-0.687423	-0.338054	-0.971881
C	-1.632375	0.608165	-0.729706
C	-2.753351	-0.060725	-0.020756
N	-2.386103	-1.466401	-0.010939
C	-1.229242	-1.582094	-0.537200
N	-1.560834	1.874365	-1.026144
C	-2.977906	0.612891	1.418998
O	-3.178143	1.851511	1.340789
O	-2.959909	-0.137247	2.402136
O	-5.508128	5.398964	-1.320973
H	3.014728	-0.865950	5.200064
H	-0.086083	-1.006727	1.959196
H	-0.917302	-2.001329	3.156684
H	1.575311	-0.526600	3.473357
H	3.306866	-3.784174	-0.263859
H	1.582829	-4.195668	-0.459478
H	2.511875	-5.870433	-1.452195
H	1.579892	-5.372989	1.681500
H	2.464651	-2.689975	2.360479
H	5.886583	-6.024011	2.897607
H	3.537106	-6.690366	1.208157
H	5.494392	-5.924456	-2.903392
H	6.382577	-4.444572	-3.350448
H	3.781995	-4.675968	-2.629189
H	7.412484	-5.664376	-1.484184
H	4.777980	-5.531333	-0.316081
H	7.244176	-0.200525	1.931376
H	6.451832	-1.700549	2.434519
H	8.002961	-1.300480	-0.200107
H	6.439190	-3.565150	0.838709
H	-4.651703	-4.892884	1.824257
H	-2.943212	-4.380726	1.690603
H	-3.595626	-2.635961	0.492911
H	-3.312885	-2.536277	3.231043
H	-5.591338	1.423669	3.060569
H	-7.316505	1.535066	3.439800
H	-6.170258	0.902838	4.652172
H	-7.444083	-0.968346	3.605396
H	-4.545763	-0.810983	3.084758
H	-6.607323	-1.469274	-2.684622
H	-5.284645	-1.843908	-1.594927
H	-6.804173	1.515921	-0.386359
H	-5.344858	3.403494	-1.410782
H	-3.747671	2.496136	-3.116423
H	-7.984665	-0.701552	-0.713822
H	-5.784944	-1.797365	0.936649
H	-7.056926	-6.975514	-0.304628
H	-6.030390	-5.753412	0.441713
H	-6.096211	-4.447243	-1.744354
H	-7.030150	-5.735348	-2.453435
H	-4.823645	-6.068122	-3.209397
H	-5.011146	-7.300836	-1.971063
H	-3.647855	-5.964139	-0.389457
H	-3.709218	-4.571852	-1.435249
H	-2.416199	-5.843100	-3.066555
H	-1.051118	-5.413675	-1.679891
H	-2.341803	-7.110497	-2.042503
H	-8.824242	-5.175586	-0.682880
H	-7.293498	-3.387331	1.108081
H	4.790491	0.908736	5.083290
H	6.670156	1.366450	3.494267
H	5.094738	1.489033	2.716524
H	5.997448	2.963144	3.148155
H	6.356670	3.469055	5.733659
H	5.573091	2.418136	6.926635
H	6.939329	1.816473	5.973341
H	4.025867	3.832579	4.668148
H	-0.662367	2.508597	1.480524
H	-0.166803	2.293293	3.158065
H	-0.171462	3.923308	2.438358
H	1.656812	1.594039	1.651242
H	2.708856	4.050200	2.858105
H	3.553438	2.716969	-2.135766
H	3.033112	4.334609	-1.574263
H	2.591240	1.724067	-0.299193
H	-0.816842	2.986583	-6.056300
H	-0.769204	2.467543	-4.361991
H	1.007309	0.888879	-4.804058
H	1.191698	1.453453	-6.474887
H	-1.172838	0.799395	-6.960772
H	-1.403309	0.293768	-5.297716
H	0.275449	-1.099856	-7.298026
H	-0.648987	-1.270499	-3.878829
H	-1.106844	-2.908619	-4.040282
H	-0.050117	-3.398825	-7.213270
H	-0.253726	-4.082406	-5.548643
H	1.304893	4.115483	-5.866755
H	2.809096	3.739414	-4.227270
H	-3.913638	5.010507	-4.336565
H	-3.466413	6.463664	-5.253235
H	-4.763234	6.291091	-2.761683
H	-1.640596	6.950956	-3.680638
H	-1.730614	4.192981	-4.704904
H	-1.075549	4.471315	0.120731
H	-0.759746	6.718479	-1.591521
H	-5.257080	3.857423	1.469096
H	-5.114828	5.618714	1.720702
H	-2.806884	3.775892	1.405326
H	-1.652622	2.575433	5.024441
H	-2.702073	1.277753	4.484723
H	-2.210940	1.977718	7.411302
H	-3.310138	-0.258837	7.823858
H	-3.710624	-0.358714	6.102566
H	-4.437559	0.900048	7.111555
H	-0.816713	-0.100949	7.400272
H	-0.178579	1.067006	6.228631
H	-1.122720	-0.311784	5.672171
H	-4.658909	2.549960	5.632896
H	-3.612799	2.716114	3.132739
H	7.985901	3.195898	0.425440
H	7.890502	3.616559	-1.290585
H	5.798578	2.334905	0.030762
H	5.520494	3.454013	-1.302738
H	4.506447	4.993777	0.081031
H	4.740615	3.932985	1.476931
H	8.356034	5.550212	0.593359
H	1.446581	-1.867236	-0.479858
H	3.004261	-0.467283	0.845837
H	3.592089	-1.677892	-1.556076
H	4.408647	0.260777	-1.136164
H	1.614589	-2.460174	-4.540337
H	2.920008	-2.950430	-3.454721
H	2.643576	-0.434492	-3.801057
H	0.828734	0.996536	-1.281001
H	-0.679943	-2.507162	-0.667331
H	-0.760846	2.319703	-1.492657
H	-4.879222	5.869023	-0.715293
H	-6.378477	5.766103	-1.119992
H	-3.705296	0.089190	-0.540319

C	5.942312	6.542746	1.633255
H	5.249423	6.405712	2.470430
H	6.815081	7.087161	2.010694
H	5.430486	7.172998	0.883038
C	7.977458	7.431363	-1.189785
H	7.658609	8.020240	-2.050628
H	7.907936	8.037650	-0.278847
H	9.030149	7.144496	-1.297869
H	-3.550902	3.907284	5.825463
H	3.320616	2.722977	5.853895
H	8.651132	-2.113354	1.244842
H	2.845787	-2.324439	6.235878
H	3.535397	-2.458071	4.606395
H	-0.795420	6.116450	0.647512
H	-4.012264	-3.987829	3.978093
H	-8.445997	-5.458960	1.031711
H	5.589001	-7.610297	2.186550
H	-2.358696	2.461852	-0.803002

TS2

C	2.701290	-1.925997	5.255524
C	1.422581	-2.304358	4.545089
O	0.839860	-3.369541	4.730629
N	1.036647	-1.424824	3.574448
C	0.126532	-1.863180	2.545659
C	0.637446	-3.124006	1.842303
O	-0.125104	-3.919784	1.294802
N	1.990574	-3.275851	1.835284
C	2.585441	-4.509574	1.394967
C	3.917971	-4.762795	2.093117
O	4.539139	-3.894395	2.702553
C	2.826778	-4.431911	-0.115485
O	3.278683	-5.694298	-0.609231
N	4.395510	-6.028120	1.883529
C	5.803274	-6.319755	2.054285
C	6.738155	-5.864949	0.919363
O	7.950583	-6.010470	1.041679
N	6.151334	-5.256141	-0.146772
C	6.956788	-4.661842	-1.196628
C	7.552170	-3.277263	-0.841220
O	8.294814	-2.723580	-1.649615
C	6.187917	-4.610717	-2.520833
O	4.954846	-3.911953	-2.387925
N	7.249491	-2.788331	0.380460
C	7.802742	-1.548255	0.913462
C	6.745838	-0.868728	1.793097
C	5.495518	-0.417671	1.016943
O	5.700342	0.223376	-0.053854
O	4.367502	-0.694470	1.525615
C	-4.033732	-3.600983	2.906700
C	-5.462657	-3.111608	3.028550
O	-6.435028	-3.868853	2.903968
C	-3.823327	-4.178514	1.501636
O	-4.220706	-3.252525	0.488088
N	-5.586309	-1.772947	3.205011
C	-6.873269	-1.110396	3.134510
C	-7.488592	-1.087034	1.723970
O	-8.609261	-0.611391	1.552395
C	-6.764318	0.314117	3.680384
N	-6.735717	-1.612466	0.722004
C	-7.274609	-1.770421	-0.605774
C	-7.733796	-3.201695	-0.954367
O	-8.187603	-3.419362	-2.080641
C	-6.249586	-1.310602	-1.662904
C	-5.935485	0.152694	-1.591914
N	-4.652857	0.631008	-1.360414
C	-6.756274	1.241830	-1.766948
C	-4.702104	1.960169	-1.390944
N	-5.958859	2.365925	-1.638172
N	-7.584696	-4.154364	-0.004349
C	-7.757093	-5.568417	-0.328434
C	-6.433128	-6.211686	-0.793464
C	-5.843278	-5.528265	-2.033975
C	-4.521171	-6.108807	-2.559569
C	-3.273686	-5.669626	-1.781725
N	-2.005072	-6.027744	-2.452211
C	3.457747	2.837959	4.999806
C	2.474878	2.314291	3.979790
O	1.983974	1.190464	4.096528
C	4.764659	2.018043	5.004732
C	5.478129	2.093438	3.651030
C	5.674460	2.486327	6.144047
N	2.187347	3.126347	2.931714
C	1.165509	2.759764	1.949312
C	1.486126	3.541426	0.667509
O	1.168834	4.725872	0.534886
C	-0.230233	3.061618	2.490423
N	2.181138	2.843771	-0.261775
C	2.595662	3.439555	-1.504392
C	1.487448	3.478707	-2.547331
O	0.294893	3.279556	-2.297570
N	1.869469	3.752523	-3.819695
C	0.848766	4.058638	-4.820059
C	-0.011302	5.240830	-4.287586
O	0.522363	6.200804	-3.750620
C	0.014309	2.833941	-5.229442
C	0.787179	1.573164	-5.616479
C	-0.221600	0.548658	-6.147417
N	0.342315	-0.754786	-6.486570
C	0.051970	-1.914047	-5.861132
N	-0.570453	-1.909804	-4.670424
N	0.355798	-3.084958	-6.418594
N	-1.359452	5.145534	-4.490281
C	-2.272833	5.986612	-3.719975
C	-2.412136	5.367379	-2.327124
O	-3.071960	4.333348	-2.178132
C	-3.645133	6.017569	-4.410707
O	-4.581081	6.776197	-3.688412
N	-1.785899	5.982244	-1.310395
C	-1.941822	5.492723	0.042230
C	-3.429650	5.406211	0.405121
O	-4.236080	6.248914	-0.022233
N	-3.766061	4.403648	1.225901
C	-5.109043	4.230246	1.769424
C	-5.067212	4.046808	3.297328
O	-5.663281	4.808116	4.053981
N	-4.310735	3.001326	3.693205
C	-4.078151	2.662945	5.093238
C	-2.874866	1.712477	5.130632
C	-2.586265	0.995719	6.455655
C	-3.730788	0.052600	6.852618
C	-1.268758	0.215700	6.326643
N	6.030900	5.490427	1.405024
C	7.128803	5.561221	0.433172
C	6.807686	6.513565	-0.733354
O	5.898914	6.280903	-1.514084
C	7.234265	4.097749	-0.023115
C	5.768729	3.611590	-0.059431
C	5.003300	4.591155	0.855940
P	1.139407	-4.596843	-3.104407
O	0.218887	-4.646324	-4.335132
O	0.309329	-4.985650	-1.813087
O	2.491780	-5.282533	-3.168723
O	1.406599	-2.999542	-2.750724
C	1.936118	-0.698452	-0.681931
O	2.138147	0.014829	0.507375
C	3.115567	-0.645125	-1.675105
O	3.732522	0.619534	-1.656107
C	2.375153	-2.289835	-3.519018
C	2.421641	-0.853015	-3.025732
O	1.059945	-0.337585	-2.872537
C	0.828526	-0.017274	-1.497666
N	-0.523757	-0.359390	-1.088346
C	-1.411021	0.550957	-0.584126
C	-2.492465	-0.186772	0.023943
N	-2.134481	-1.568589	-0.093949
C	-1.029676	-1.640379	-0.749237
N	-1.319937	1.865270	-0.654325
C	-3.041879	0.373761	1.344371
O	-3.175247	1.645396	1.346861
O	-3.320532	-0.415517	2.259483
O	-6.022847	5.254852	-1.727065
H	2.825587	-0.842502	5.319887
H	0.034519	-1.060539	1.811467
H	-0.871285	-2.095077	2.924609
H	1.485674	-0.519570	3.499846
H	3.566256	-3.649465	-0.326221
H	1.873323	-4.162597	-0.556763
H	2.961171	-5.759993	-1.540007
H	1.880978	-5.326156	1.582037
H	2.599762	-2.600377	2.280739
H	6.154084	-5.808720	2.952851
H	3.894874	-6.556514	1.177295
H	6.011917	-5.636526	-2.867023
H	6.833006	-4.099254	-3.243397
H	4.216868	-4.484584	-2.669413
H	7.843434	-5.288296	-1.349249
H	5.146527	-5.335530	-0.306196
H	7.191827	0.025619	2.243382
H	6.430305	-1.528761	2.606703
H	8.062423	-0.911814	0.066191
H	6.589684	-3.298145	0.952407
H	-4.449117	-5.068416	1.386514
H	-2.769867	-4.453514	1.375031
H	-3.443711	-2.660999	0.332312
H	-3.323395	-2.790705	3.082168
H	-6.008987	0.882282	3.129151
H	-7.725674	0.818923	3.576074
H	-6.474071	0.297533	4.734114
H	-7.585853	-1.688608	3.730965
H	-4.754349	-1.197773	3.062462
H	-6.653616	-1.574009	-2.644022
H	-5.324401	-1.879396	-1.525250
H	-7.814956	1.304811	-1.959000
H	-6.207080	3.358059	-1.717096
H	-3.879931	2.643990	-1.280423
H	-8.169141	-1.144013	-0.657982
H	-5.816207	-2.012836	0.900746
H	-6.619100	-7.274408	-1.004645
H	-5.715513	-6.176169	0.034664
H	-5.678741	-4.472165	-1.805636
H	-6.591998	-5.550407	-2.832310
H	-4.392128	-5.776059	-3.599502
H	-4.566226	-7.207370	-2.596701
H	-3.252176	-6.108177	-0.780211
H	-3.285745	-4.586156	-1.631044
H	-1.990745	-5.681422	-3.412196
H	-0.620137	-5.398179	-1.983804
H	-1.914360	-7.039975	-2.518374
H	-8.507883	-5.631403	-1.119773
H	-7.179943	-3.915377	0.898149
H	4.485677	0.973057	5.184102
H	6.418806	1.536899	3.689159
H	4.872076	1.651985	2.855780
H	5.718342	3.129233	3.380980
H	5.978164	3.530339	5.998011
H	5.173108	2.414322	7.115423
H	6.584698	1.879854	6.188304
H	3.690987	3.896155	4.822737
H	-1.005091	2.638177	1.851043
H	-0.335942	2.593338	3.470159
H	-0.378333	4.140763	2.582970
H	1.275962	1.690484	1.765155
H	2.494038	4.088154	2.951113
H	3.426617	2.852140	-1.896206

H	2.914572	4.475349	-1.340556
H	2.431203	1.865531	-0.083276
H	-0.615654	3.141221	-6.074743
H	-0.642635	2.571817	-4.395019
H	1.294911	1.165695	-4.740135
H	1.544332	1.790226	-6.381276
H	-0.718411	0.945100	-7.041996
H	-1.004225	0.378242	-5.405345
H	0.910155	-0.816663	-7.318055
H	-0.242196	-1.213649	-3.994709
H	-0.619594	-2.853739	-4.273211
H	0.719970	-3.130379	-7.356350
H	0.398454	-3.903930	-5.750756
H	1.384257	4.435890	-5.697600
H	2.802682	4.108865	-3.966518
H	-3.979992	4.979168	-4.550740
H	-3.516508	6.468974	-5.401223
H	-5.028012	6.190021	-3.046657
H	-1.851134	6.990710	-3.668225
H	-1.723215	4.217137	-4.650399
H	-1.434190	4.535743	0.176742
H	-1.148097	6.738106	-1.506171
H	-5.584343	3.361802	1.297909
H	-5.684106	5.127054	1.551736
H	-3.117922	3.645406	1.405439
H	-1.988241	2.284471	4.832838
H	-3.013799	0.945445	4.357063
H	-2.467888	1.749877	7.249067
H	-3.482987	-0.503882	7.762814
H	-3.919590	-0.676867	6.055902
H	-4.665055	0.591805	7.038790
H	-1.005057	-0.279890	7.266976
H	-0.435649	0.865908	6.038874
H	-1.350091	-0.558845	5.555889
H	-4.974727	2.194736	5.517179
H	-3.919406	2.375617	2.982987
H	7.805663	3.544315	0.726724
H	7.741204	3.986597	-0.985023
H	5.685800	2.569740	0.254206
H	5.385339	3.684699	-1.077012
H	4.244156	5.150951	0.288850
H	4.491491	4.082054	1.678396
H	8.035730	5.912243	0.941812
H	1.671029	-1.744453	-0.492044
H	3.045518	-0.260459	0.909592
H	3.845069	-1.439598	-1.482772
H	4.510560	0.535567	-1.007765
H	2.108153	-2.316360	-4.581117
H	3.370741	-2.729527	-3.416804
H	2.933800	-0.233278	-3.768527
H	0.914543	1.058567	-1.368540
H	-0.504310	-2.543893	-1.032292
H	-0.614103	2.394136	-1.175020
H	-5.390286	5.584876	-1.032642
H	-6.824226	5.776631	-1.589060
H	-3.577046	0.083751	-0.755129
C	5.532362	6.756544	1.902823
H	4.821422	6.571272	2.715364
H	6.358147	7.347726	2.313831
H	5.009351	7.362867	1.140517
C	7.621421	7.792131	-0.829285
H	7.301827	8.377862	-1.692061
H	7.492344	8.380443	0.086964
H	8.689994	7.559156	-0.906262
H	-3.898480	3.582610	5.659161
H	2.973253	2.763088	5.979157
H	8.711884	-1.753648	1.493695
H	2.716040	-2.374573	6.250393
H	3.533483	-2.340147	4.674010
H	-1.454959	6.196962	0.720055
H	-3.860332	-4.387251	3.649424
H	-8.135336	-6.081585	0.560192
H	5.946828	-7.392795	2.205729
H	-2.037185	2.323549	-0.099851

E:CAIR

C	-2.727663	-1.847349	-5.245998
C	-1.460569	-2.259830	-4.537686
O	-0.903938	-3.338228	-4.728130
N	-1.057463	-1.395776	-3.561416
C	-0.170688	-1.866438	-2.526052
C	-0.715612	-3.134218	-1.860983
O	0.018447	-3.981668	-1.356177
N	-2.075827	-3.232087	-1.837926
C	-2.708737	-4.453753	-1.418566
C	-4.046095	-4.662729	-2.120492
O	-4.649310	-3.770301	-2.713184
C	-2.949239	-4.390673	0.092853
O	-3.433573	-5.648360	0.569709
N	-4.551725	-5.921306	-1.934085
C	-5.965539	-6.177852	-2.109827
C	-6.890937	-5.717489	-0.969555
O	-8.106669	-5.828111	-1.097422
N	-6.291141	-5.144304	0.108697
C	-7.084478	-4.541442	1.162657
C	-7.639207	-3.136838	0.821230
O	-8.381509	-2.578421	1.626937
C	-6.320150	-4.526931	2.490150
O	-5.072750	-3.853157	2.370547
N	-7.302116	-2.633583	-0.385342
C	-7.825260	-1.376506	-0.908509
C	-6.755323	-0.715092	-1.785292
C	-5.498458	-0.283711	-1.007747
O	-5.692982	0.358713	0.063639
O	-4.375719	-0.576358	-1.519602
C	3.971212	-3.655747	-2.892732
C	5.417337	-3.216180	-3.006183
O	6.364848	-4.000163	-2.843927
C	3.727302	-4.185852	-1.474231
O	4.113366	-3.227971	-0.483262
N	5.584093	-1.888812	-3.215551
C	6.885249	-1.258672	-3.145529
C	7.485687	-1.219997	-1.730862
O	8.614766	-0.767270	-1.554173
C	6.814081	0.157124	-3.722172
N	6.714496	-1.709341	-0.718725
C	7.278053	-1.884820	0.595045
C	7.687799	-3.330671	0.952684
O	8.126596	-3.549881	2.085156
C	6.306737	-1.385856	1.685625
C	6.000772	0.074993	1.593347
N	4.744878	0.548179	1.240738
C	6.757477	1.195440	1.821579
C	4.728461	1.882523	1.251771
N	5.943266	2.298718	1.607246
N	7.516841	-4.281871	0.007961
C	7.652416	-5.699308	0.343454
C	6.308033	-6.307481	0.797695
C	5.703061	-5.577420	2.004508
C	4.378621	-6.141582	2.543442
C	3.130243	-5.711353	1.761363
N	1.862595	-6.063376	2.434658
C	-3.391857	2.930037	-4.984620
C	-2.418752	2.389084	-3.962032
O	-1.936938	1.261929	-4.084252
C	-4.711626	2.131388	-4.995298
C	-5.426351	2.214097	-3.642714
C	-5.611086	2.618310	-6.135104
N	-2.128334	3.192923	-2.907217
C	-1.087555	2.827484	-1.942417
C	-1.391108	3.601819	-0.650108
O	-1.049535	4.778396	-0.503813
C	0.293774	3.142675	-2.512571
N	-2.105142	2.909667	0.268135
C	-2.515802	3.501186	1.514388
C	-1.406535	3.531069	2.556541
O	-0.218951	3.307548	2.309868
N	-1.779422	3.816560	3.830506
C	-0.742908	4.064080	4.827545
C	0.194868	5.185552	4.292558
O	-0.264546	6.163520	3.718977
C	0.021206	2.791431	5.233903
C	-0.812030	1.564078	5.603650
C	0.153225	0.491304	6.120555
N	-0.458978	-0.795107	6.440647
C	-0.165841	-1.965655	5.836165
N	0.487832	-1.982548	4.662498
N	-0.499335	-3.126839	6.398322
N	1.529434	5.023662	4.539970
C	2.512147	5.819318	3.807676
C	2.660701	5.225018	2.403402
O	3.396621	4.248160	2.218434
C	3.862372	5.769644	4.536786
O	4.856134	6.499219	3.862493
N	1.960085	5.810688	1.418584
C	2.093483	5.353636	0.051567
C	3.572748	5.301228	-0.346793
O	4.375686	6.154657	0.070162
N	3.909410	4.312520	-1.184292
C	5.238804	4.179895	-1.769785
C	5.163364	3.988513	-3.296057
O	5.746923	4.749545	-4.063565
N	4.405109	2.939573	-3.674386
C	4.139322	2.609036	-5.070790
C	2.918606	1.681216	-5.091255
C	2.589651	0.984762	-6.417762
C	3.707169	0.023407	-6.846812
C	1.259882	0.229086	-6.270437
N	-5.914818	5.627069	-1.387084
C	-7.010695	5.717340	-0.414714
C	-6.672502	6.664086	0.751333
O	-5.767342	6.416940	1.531829
C	-7.142293	4.256028	0.041854
C	-5.685967	3.742938	0.076657
C	-4.903593	4.708427	-0.839246
P	-1.287730	-4.622441	3.087638
O	-0.368314	-4.695900	4.319384
O	-0.468546	-5.040013	1.797798
O	-2.655775	-5.276236	3.149892
O	-1.514282	-3.019940	2.735576
C	-1.970380	-0.638707	0.701810
O	-2.151612	0.108520	-0.471452
C	-3.147111	-0.579684	1.694431
O	-3.737424	0.698751	1.709523
C	-2.466567	-2.287984	3.504060
C	-2.457770	-0.840091	3.038753
O	-1.080026	-0.370479	2.896597
C	-0.836569	-0.016245	1.528643
N	0.497087	-0.391641	1.094571
C	1.351676	0.509095	0.477628
C	2.260309	-0.242239	-0.274595
N	1.963000	-1.599871	-0.083895
C	0.933741	-1.671456	0.711582
N	1.321962	1.845701	0.612511
C	3.043718	0.327952	-1.403960
O	3.229753	1.605733	-1.353810
O	3.441867	-0.422883	-2.316983
O	6.182812	5.094804	1.698444
H	-2.824484	-0.760722	-5.307144
H	-0.082440	-1.079448	-1.775931
H	0.831265	-2.103226	-2.890609
H	-1.483323	-0.479634	-3.484726
H	-3.668533	-3.592745	0.315171
H	-1.989306	-4.151300	0.537155
H	-3.126582	-5.730800	1.501915

H	-2.026151	-5.286125	-1.616300
H	-2.664466	-2.523239	-2.258491
H	-6.303691	-5.645825	-3.001033
H	-4.063419	-6.473148	-1.237306
H	-6.167803	-5.561597	2.821334
H	-6.957388	-4.013035	3.218278
H	-4.347865	-4.440918	2.656150
H	-7.988752	-5.144786	1.303896
H	-5.288451	-5.249637	0.268425
H	-7.185945	0.186826	-2.235547
H	-6.449664	-1.379651	-2.598932
H	-8.068541	-0.739260	-0.056911
H	-6.651151	-3.152242	-0.959602
H	4.339198	-5.078936	-1.317399
H	2.667786	-4.441463	-1.358687
H	3.324025	-2.633636	-0.381209
H	3.290816	-2.827192	-3.098139
H	6.060801	0.749142	-3.193979
H	7.784170	0.645048	-3.617583
H	6.536288	0.122346	-4.778720
H	7.592274	-1.865370	-3.720355
H	4.769890	-1.283327	-3.081006
H	6.750669	-1.626445	2.654202
H	5.371306	-1.949902	1.602363
H	7.791803	1.290490	2.106246
H	6.169627	3.306333	1.695432
H	3.885298	2.521283	1.063992
H	8.196718	-1.292143	0.620112
H	5.788673	-2.101676	-0.893105
H	6.474943	-7.365318	1.045047
H	5.608488	-6.288331	-0.046327
H	5.535082	-4.532540	1.728878
H	6.445486	-5.563296	2.808667
H	4.253352	-5.789699	3.577486
H	4.419329	-7.239279	2.601093
H	3.109660	-6.159942	0.764202
H	3.141976	-4.629136	1.599520
H	1.845795	-5.709371	3.391727
H	0.462622	-5.440351	1.966655
H	1.770479	-7.074858	2.508467
H	8.392363	-5.773646	1.143665
H	7.122712	-4.040810	-0.900527
H	-4.449572	1.082517	-5.177658
H	-6.379403	1.679402	-3.686143
H	-4.831991	1.753792	-2.849407
H	-5.642828	3.253365	-3.365952
H	-5.899031	3.666307	-5.985416
H	-5.108331	2.542712	-7.105539
H	-6.530476	2.026148	-6.184266
H	-3.609023	3.991424	-4.805982
H	1.086156	2.693918	-1.913992
H	0.367716	2.708368	-3.510652
H	0.443754	4.223867	-2.571979
H	-1.188837	1.756791	-1.760992
H	-2.423161	4.158480	-2.929362
H	-3.345811	2.910731	1.904790
H	-2.836479	4.537464	1.357015
H	-2.357678	1.931441	0.083049
H	0.660075	3.056672	6.087035
H	0.668323	2.504363	4.399821
H	-1.334663	1.189301	4.721748
H	-1.559341	1.805549	6.370739
H	0.666052	0.856479	7.019983
H	0.926921	0.301884	5.374265
H	-1.039178	-0.842990	7.264581
H	0.179355	-1.287235	3.973748
H	0.520693	-2.930576	4.272748
H	-0.910636	-3.152998	7.317285
H	-0.551266	-3.946867	5.731113
H	-1.250761	4.474701	5.706663
H	-2.703871	4.189727	3.988550
H	4.144650	4.713835	4.666171
H	3.728838	6.208840	5.531576
H	5.245539	5.934382	3.167977
H	2.148310	6.845972	3.757080
H	1.840891	4.084750	4.740418
H	1.600636	4.392010	-0.097785
H	1.232779	6.467859	1.662748
H	5.758523	3.327412	-1.314807
H	5.794099	5.093561	-1.571323
H	3.275620	3.536942	-1.354813
H	2.049426	2.266142	-4.769342
H	3.060805	0.904299	-4.328828
H	2.470767	1.749096	-7.201467
H	3.431665	-0.517083	-7.758759
H	3.894602	-0.719094	-6.061913
H	4.649038	0.545585	-7.043653
H	0.969638	-0.254095	-7.209504
H	0.444430	0.892105	-5.962428
H	1.340446	-0.552830	-5.507160
H	5.019377	2.125432	-5.512186
H	4.012877	2.319432	-2.956849
H	-7.724517	3.713079	-0.707325
H	-7.650356	4.154299	1.004248
H	-5.622696	2.699902	-0.237663
H	-5.299908	3.808077	1.093795
H	-4.133603	5.254027	-0.272867
H	-4.402031	4.189673	-1.661795
H	-7.911522	6.084605	-0.922852
H	-1.734037	-1.686141	0.483799
H	-3.047529	-0.163325	-0.898211
H	-3.895961	-1.351105	1.483269
H	-4.502081	0.647920	1.046309
H	-2.215129	-2.343835	4.568642
H	-3.475625	-2.690168	3.381995
H	-2.949757	-0.219770	3.795097
H	-0.889671	1.065644	1.437982
H	0.443273	-2.573015	1.050422
H	0.620841	2.373346	1.127430
H	5.525290	5.479300	1.051921
H	7.006019	5.569488	1.525089
H	3.940001	0.001765	0.900744
C	-5.393254	6.884007	-1.884431
H	-4.686316	6.686032	-2.697479
H	-6.208176	7.490700	-2.294643
H	-4.858510	7.480202	-1.122180
C	-7.464627	7.956286	0.846990
H	-7.135329	8.536817	1.709665
H	-7.325418	8.542111	-0.069381
H	-8.537006	7.741451	0.923807
H	3.967973	3.534234	-5.630335
H	-2.904673	2.849416	-5.962137
H	-8.740503	-1.556099	-1.487974
H	-2.755577	-2.291838	-6.242513
H	-3.570125	-2.241126	-4.664994
H	1.578495	6.064503	-0.597983
H	3.781925	-4.457275	-3.614896
H	8.028608	-6.226033	-0.537794
H	-6.133621	-7.245042	-2.276996
H	1.948001	2.287294	-0.049564

E:AIR:CO_2'

C	2.853964	-2.028087	5.393377
C	1.477346	-2.227296	4.817839
O	0.644119	-2.985766	5.303025
N	1.253394	-1.552896	3.644768
C	0.189142	-2.011163	2.790490
C	0.487731	-3.323317	2.066953
O	-0.410258	-3.903380	1.442833
N	1.756939	-3.780201	2.123832
C	2.162976	-4.947089	1.378979
C	3.501793	-5.465227	1.906307
O	4.204467	-4.835398	2.690771
C	2.295393	-4.577585	-0.108753
O	2.619101	-5.738915	-0.873227
N	3.856669	-6.671410	1.362241
C	5.235299	-7.111698	1.377262
C	6.137136	-6.586188	0.246591
O	7.312256	-6.937729	0.202570
N	5.581278	-5.690445	-0.614169
C	6.385541	-5.045195	-1.636636
C	7.230202	-3.854010	-1.123517
O	7.978666	-3.265413	-1.900821
C	5.520374	-4.625612	-2.830042
O	4.454494	-3.775368	-2.429304
N	7.111319	-3.571176	0.192002
C	7.771508	-2.449421	0.847936
C	6.757752	-1.801701	1.816734
C	5.496914	-1.285283	1.094554
O	5.685395	-0.319492	0.292815
O	4.391917	-1.850181	1.328905
C	-4.117512	-3.088044	3.403861
C	-5.550232	-2.635505	3.595138
O	-6.508727	-3.400272	3.419796
C	-3.927494	-3.418608	1.917038
O	-4.412545	-2.342145	1.111974
N	-5.706193	-1.315163	3.857941
C	-7.015279	-0.695524	3.932809
C	-7.632447	-0.352117	2.561979
O	-8.642593	0.352024	2.508142
C	-7.017247	0.523006	4.853949
N	-7.062627	-0.924639	1.471707
C	-7.787883	-1.029739	0.218401
C	-8.385115	-2.430380	-0.035603
O	-9.074850	-2.607503	-1.040195
C	-6.896790	-0.692436	-0.983715
C	-6.489498	0.741190	-1.079376
N	-5.622576	1.140724	-2.084710
C	-6.844262	1.829986	-0.315429
C	-5.459626	2.443633	-1.928388
N	-6.179002	2.910215	-0.874324
N	-8.044333	-3.417600	0.828092
C	-8.167319	-4.815055	0.434538
C	-6.874598	-5.316641	-0.257587
C	-6.518990	-4.498324	-1.506929
C	-5.250791	-4.885462	-2.285929
C	-3.914502	-4.522517	-1.609900
N	-2.802717	-4.324555	-2.571164
C	4.101429	2.603668	4.824344
C	3.323296	1.879346	3.743530
O	3.289707	0.648666	3.685228
C	5.533238	2.039673	4.934976
C	6.288714	2.124635	3.601838
C	6.296204	2.747767	6.057873
N	2.706695	2.660987	2.825672
C	1.966663	2.082885	1.711654
C	2.010915	3.126017	0.593182
O	1.551115	4.263471	0.770490
C	0.539963	1.714625	2.132419
N	2.621305	2.718722	-0.536081
C	2.771609	3.585293	-1.672721
C	1.540395	3.614072	-2.561791
O	0.414608	3.381073	-2.124653
N	1.731028	3.996979	-3.856519
C	0.604154	4.582726	-4.587530
C	0.016221	5.712500	-3.684613
O	0.758668	6.388979	-2.989194
C	-0.456636	3.564513	-5.040190
C	0.040586	2.232220	-5.603189
C	-1.182046	1.456333	-6.119693
N	-0.913622	0.087458	-6.549454
C	-0.784634	-0.972867	-5.717319
N	-1.063156	-0.845985	-4.417740
N	-0.408738	-2.155406	-6.198124
N	-1.345822	5.875612	-3.699897

C	-2.003121	6.342861	-2.474964
C	-2.222337	5.081601	-1.631104
O	-2.950359	4.175227	-2.057399
C	-3.339152	7.026985	-2.787328
O	-3.942316	7.527612	-1.619638
N	-1.564468	4.989317	-0.461942
C	-1.525111	3.730662	0.250156
C	-2.829267	3.384981	0.950818
O	-3.586515	4.263561	1.414902
N	-3.047644	2.076638	1.140438
C	-4.175591	1.547640	1.908809
C	-3.909695	1.673760	3.416128
O	-3.635616	0.693334	4.113594
N	-3.988687	2.934653	3.909593
C	-3.398875	3.255288	5.211140
C	-1.868668	3.314831	5.077810
C	-1.074598	3.581081	6.364109
C	-1.309272	2.491475	7.418275
C	0.419272	3.689686	6.024402
N	6.737656	4.758115	0.951695
C	7.778482	4.642960	-0.078103
C	7.530525	5.595987	-1.260301
O	6.559399	5.467344	-1.988473
C	7.657946	3.166033	-0.492470
C	6.154464	2.833085	-0.332024
C	5.568634	4.002791	0.482233
P	0.653710	-4.044100	-3.367839
O	-0.202026	-4.203161	-4.616865
O	-0.201680	-4.283379	-2.053556
O	1.994483	-4.749768	-3.273474
O	0.942324	-2.405445	-3.207263
C	1.858763	-1.046475	-0.741997
O	2.079314	-0.830161	0.623326
C	3.085891	-0.795605	-1.643759
O	3.793032	0.361877	-1.242220
C	2.068903	-1.767762	-3.813161
C	2.423968	-0.530030	-2.993248
O	1.217431	0.213347	-2.675135
C	0.850015	-0.033139	-1.319439
N	-0.555700	-0.452163	-1.236441
C	-1.630447	0.350036	-1.614155
C	-2.753839	-0.229864	-1.075293
N	-2.385973	-1.371326	-0.378447
C	-1.079742	-1.484603	-0.485034
N	-1.505178	1.391331	-2.539330
C	-4.321814	-0.477007	-3.938096
O	-3.973330	0.481866	-4.511912
O	-4.630208	-1.491557	-3.445921
O	-5.318115	5.593981	-0.130162
H	3.254293	-1.035818	5.179782
H	-0.016926	-1.259896	2.029096
H	-0.713861	-2.177899	3.380896
H	1.983861	-0.958592	3.265843
H	3.075063	-3.812784	-0.213456
H	1.331414	-4.181053	-0.424446
H	2.306394	-5.560702	-1.792930
H	1.388596	-5.715162	1.468746
H	2.501921	-3.221198	2.522337
H	5.687638	-6.769756	2.310574
H	3.289390	-6.958073	0.570874
H	5.127289	-5.526873	-3.316667
H	6.179834	-4.103938	-3.532696
H	3.606012	-4.153684	-2.741414
H	7.132328	-5.763173	-1.994722
H	4.568470	-5.583771	-0.672684
H	7.235669	-0.953169	2.319223
H	6.452027	-2.526197	2.578120
H	8.076841	-1.743146	0.073433
H	6.374311	-4.041133	0.700552
H	-4.503303	-4.321775	1.683648
H	-2.869289	-3.611768	1.708605
H	-3.644274	-2.011427	0.572625
H	-3.412642	-2.308420	3.708826
H	-6.351687	1.308096	4.484873
H	-8.027110	0.931925	4.895829
H	-6.690298	0.241280	5.857928
H	-7.696789	-1.456344	4.332723
H	-4.881856	-0.723976	3.954275
H	-7.437377	-0.985508	-1.887806
H	-5.996871	-1.318063	-0.944495
H	-7.494944	1.917927	0.541801
H	-6.140130	3.872678	-0.542672
H	-4.824781	3.084739	-2.518461
H	-8.626246	-0.332264	0.272092
H	-6.169557	-1.412393	1.538348
H	-7.009241	-6.374676	-0.522892
H	-6.052935	-5.275960	0.468018
H	-6.404595	-3.452972	-1.213356
H	-7.374109	-4.522342	-2.191239
H	-5.288808	-4.339085	-3.237417
H	-5.260554	-5.954146	-2.544750
H	-3.607223	-5.292512	-0.895292
H	-4.017273	-3.594175	-1.044391
H	-2.989464	-3.482432	-3.114366
H	-1.223178	-4.270140	-2.176011
H	-2.777570	-5.084869	-3.250361
H	-9.017985	-4.890257	-0.246452
H	-7.479210	-3.216115	1.649206
H	5.428106	0.979374	5.192232
H	7.295760	1.707778	3.709707
H	5.784919	1.553419	2.816854
H	6.400996	3.158962	3.255299
H	6.424380	3.813655	5.831932
H	5.769840	2.668877	7.015710
H	7.293859	2.315134	6.184936
H	4.134289	3.682540	4.625883
H	-0.016784	1.279411	1.298308
H	0.586821	0.969959	2.928956
H	0.005069	2.590518	2.507517
H	2.476236	1.169087	1.404613
H	2.673174	3.664768	2.932273
H	3.658677	3.284072	-2.234904
H	2.907614	4.619972	-1.334396
H	3.012955	1.762372	-0.638990
H	-1.076992	4.063175	-5.798456
H	-1.106274	3.331362	-4.194898
H	0.537575	1.662134	-4.812746
H	0.766758	2.379824	-6.411856
H	-1.620276	1.973782	-6.978525
H	-1.958209	1.436379	-5.351104
H	-0.759139	-0.081298	-7.530197
H	-1.231407	0.077527	-4.010570
H	-0.569459	-1.493905	-3.797134
H	-0.110404	-2.222420	-7.158028
H	-0.326291	-3.020792	-5.559870
H	1.037897	5.060821	-5.471932
H	2.641832	4.378009	-4.072917
H	-3.984922	6.311031	-3.317681
H	-3.140379	7.866412	-3.462133
H	-4.435926	6.804516	-1.182593
H	-1.334588	7.049011	-1.983046
H	-1.873200	5.135524	-4.142890
H	-1.218303	2.926078	-0.418363
H	-0.807592	5.633991	-0.281695
H	-4.282358	0.490851	1.666750
H	-5.088841	2.072015	1.620688
H	-2.488828	1.401789	0.626408
H	-1.622352	4.084756	4.333970
H	-1.540130	2.355788	4.659179
H	-1.401878	4.545105	6.782649
H	-0.693849	2.668516	8.306477
H	-1.046381	1.505654	7.018450
H	-2.353186	2.450202	7.743096
H	1.016352	3.903312	6.917139
H	0.606345	4.483709	5.293076
H	0.784395	2.750154	5.593528
H	-3.702059	2.474310	5.909793
H	-4.041794	3.674218	3.212490
H	8.255844	2.561826	0.194224
H	8.026190	2.985401	-1.505372
H	6.027629	1.870827	0.163550
H	5.667483	2.761723	-1.305681
H	4.918148	4.630960	-0.145206
H	4.981346	3.671182	1.344682
H	8.752285	4.877727	0.370352
H	1.492530	-2.056670	-0.943186
H	2.986530	-1.207875	0.867164
H	3.752079	-1.663282	-1.675208
H	4.546837	0.075553	-0.598846
H	1.807842	-1.474352	-4.837189
H	2.926455	-2.444598	-3.849582
H	3.066153	0.125823	-3.588830
H	0.922796	0.914197	-0.782677
H	-0.496063	-2.282790	-0.057677
H	-0.653854	1.942311	-2.425384
H	-4.597653	5.189742	0.413841
H	-5.822465	6.135565	0.490991
H	-3.781175	0.075513	-1.203689
C	6.443759	6.095063	1.422365
H	5.754349	6.034556	2.271169
H	7.361914	6.579433	1.773340
H	5.972732	6.744969	0.661317
C	8.509560	6.743833	-1.443412
H	8.227878	7.344812	-2.309023
H	8.517668	7.369606	-0.543160
H	9.527972	6.357552	-1.568195
H	-3.813492	4.208112	5.553367
H	3.574058	2.460521	5.775053
H	8.662540	-2.785608	1.392906
H	2.830721	-2.221221	6.467390
H	3.518508	-2.765854	4.926192
H	-0.747286	3.825879	1.008895
H	-3.934359	-3.982339	4.006818
H	-8.373553	-5.413668	1.326345
H	5.286799	-8.203755	1.363971
H	-2.297405	2.027254	-2.474852

TS1’

C	3.034114	-1.315534	5.238967
C	1.756617	-1.742991	4.579063
O	1.107654	-2.725592	4.928162
N	1.438589	-1.025164	3.463159
C	0.614241	-1.659926	2.471526
C	1.224347	-2.970977	1.968796
O	0.526278	-3.870654	1.504626
N	2.580745	-3.048300	2.052313
C	3.273340	-4.239408	1.635645
C	4.652891	-4.332415	2.279364
O	5.205602	-3.383854	2.834138
C	3.442337	-4.202687	0.112324
O	4.001769	-5.433153	-0.348746
N	5.247573	-5.549499	2.085186
C	6.686480	-5.688787	2.178604
C	7.504630	-5.183563	0.975245
O	8.730377	-5.198888	1.035078
N	6.800212	-4.675673	-0.071768
C	7.478026	-4.020494	-1.175118
C	7.945760	-2.576228	-0.866991
O	8.584360	-1.962529	-1.718972
C	6.638897	-4.076592	-2.458025
O	5.341144	-3.521388	-2.276641
N	7.662160	-2.104961	0.366859
C	8.085961	-0.797538	0.853581
C	6.967769	-0.208255	1.725617
C	5.659470	0.087636	0.964338

O	5.765455	0.629047	-0.168822
O	4.573388	-0.202988	1.561235
C	-3.530497	-3.719780	3.046158
C	-5.031891	-3.501636	3.039256
O	-5.827714	-4.439423	2.928444
C	-3.127652	-4.345465	1.703873
O	-3.659033	-3.592697	0.615687
N	-5.432457	-2.201205	3.087902
C	-6.813088	-1.802087	2.858472
C	-7.173435	-1.663419	1.364948
O	-8.242312	-1.151494	1.030167
C	-7.165444	-0.542541	3.638318
N	-6.303235	-2.221402	0.491775
C	-6.660668	-2.493470	-0.880271
C	-7.019603	-3.981673	-1.091715
O	-7.347132	-4.384212	-2.208702
C	-5.545667	-2.055213	-1.849228
C	-5.294831	-0.572305	-1.838089
N	-4.071271	-0.033149	-2.218542
C	-6.150781	0.441755	-1.468874
C	-4.195910	1.275187	-2.079218
N	-5.437929	1.617740	-1.625624
N	-6.952471	-4.766653	0.012855
C	-7.069059	-6.216182	-0.027436
C	-5.702442	-6.885955	-0.280898
C	-5.152722	-6.596949	-1.684336
C	-3.731599	-7.099582	-1.974287
C	-2.616402	-6.299011	-1.285925
N	-1.274002	-6.548529	-1.849420
C	3.377375	3.467251	4.655808
C	2.365015	3.039683	3.612344
O	1.862436	1.916567	3.625036
C	4.700932	2.684609	4.506411
C	5.353676	2.940665	3.144187
C	5.649945	3.028263	5.657673
N	2.070668	3.968311	2.657077
C	1.016242	3.753685	1.676782
C	1.464684	4.143381	0.256854
O	1.224661	5.255502	-0.212928
C	-0.244705	4.538279	2.052053
N	2.091596	3.172057	-0.456007
C	2.315784	3.365045	-1.867366
C	0.999529	3.325906	-2.648644
O	-0.055849	2.911415	-2.143978
N	1.030115	3.700366	-3.942907
C	-0.133888	3.693937	-4.845421
C	-1.166865	4.810289	-4.562133
O	-1.452926	5.638368	-5.422572
C	-0.916031	2.366625	-4.914581
C	-0.136258	1.111747	-5.296327
C	-1.132380	-0.039002	-5.490064
N	-0.510634	-1.269314	-5.975693
C	-0.413504	-2.432918	-5.307614
N	-0.755462	-2.515761	-4.014940
N	0.001763	-3.540087	-5.927195
N	-1.788750	4.716790	-3.362243
C	-2.863926	5.600554	-2.990066
C	-3.018365	5.564877	-1.476423
O	-2.572081	4.649933	-0.777940
C	-4.194418	5.174010	-3.669701
O	-5.265388	6.026290	-3.298310
N	-3.710415	6.620418	-0.975917
C	-4.065543	6.650242	0.431588
C	-5.264416	5.749479	0.716221
O	-6.420015	6.162450	0.530390
N	-4.975302	4.497135	1.124969
C	-5.996458	3.591709	1.622462
C	-5.797685	3.152891	3.078127
O	-6.721691	2.614056	3.674482
N	-4.566408	3.369421	3.616001
C	-4.115452	2.651638	4.801921
C	-3.195668	1.488185	4.410177
C	-2.650871	0.646519	5.573700
C	-3.769465	-0.140746	6.269380
C	-1.555862	-0.295444	5.060741
N	5.669451	6.073427	0.830713
C	6.760183	6.156786	-0.149293
C	6.381198	7.024542	-1.362853
O	5.501036	6.687342	-2.137678
C	6.946423	4.679336	-0.532229
C	5.512676	4.100416	-0.517339
C	4.687154	5.108165	0.310517
P	1.631477	-4.815944	-2.735076
O	0.634374	-5.054734	-3.877111
O	1.001856	-5.315279	-1.371048
O	3.074464	-5.262636	-2.885419
O	1.652348	-3.178953	-2.445476
C	2.077115	-0.504279	-0.552182
O	2.337156	0.384390	0.500553
C	3.146414	-0.574153	-1.663063
O	3.777983	0.667026	-1.866923
C	2.381861	-2.370562	-3.364950
C	2.314165	-0.924728	-2.911435
O	0.921917	-0.578248	-2.620368
C	0.851992	-0.006157	-1.322963
N	-0.418895	-0.324579	-0.685957
C	-1.057621	0.530487	0.187427
C	-1.976906	-0.249250	0.924853
N	-1.872351	-1.574186	0.456604
C	-0.952048	-1.595220	-0.459299
N	-0.834362	1.848191	0.262944
C	-3.853955	0.880766	1.060290
O	-3.502629	2.023655	0.946250
O	-4.677599	0.075396	1.349143
O	-6.753124	4.406460	-1.554523
H	3.176862	-0.232467	5.179217
H	0.510731	-0.965782	1.642430
H	-0.375673	-1.911074	2.852309
H	1.923567	-0.165668	3.235087
H	4.090457	-3.360836	-0.159846
H	2.450252	-4.046867	-0.297745
H	3.660800	-5.577890	-1.263444
H	2.658827	-5.109419	1.885861
H	3.145280	-2.258818	2.345200
H	7.033370	-5.110272	3.036851
H	4.771740	-6.143746	1.414215
H	6.566073	-5.121629	-2.783662
H	7.187384	-3.508310	-3.217180
H	4.664472	-4.182910	-2.515502
H	8.415961	-4.553235	-1.371447
H	5.804314	-4.875828	-0.177450
H	7.319699	0.739695	2.150719
H	6.734125	-0.874188	2.562024
H	8.266437	-0.166339	-0.017872
H	7.081534	-2.668303	0.972899
H	-3.553992	-5.350230	1.643327
H	-2.035001	-4.408400	1.637531
H	-3.043979	-2.819128	0.515038
H	-2.988360	-2.781174	3.199837
H	-6.525974	0.294346	3.356206
H	-8.192449	-0.253220	3.414108
H	-7.060627	-0.715392	4.712690
H	-7.423840	-2.643057	3.204547
H	-4.732915	-1.483955	2.959027
H	-5.826044	-2.402258	-2.849596
H	-4.616407	-2.570963	-1.583984
H	-7.142734	0.420659	-1.044072
H	-5.784742	2.559139	-1.462316
H	-3.420466	2.011502	-2.239868
H	-7.577181	-1.935244	-1.089554
H	-5.386548	-2.547573	0.788772
H	-5.803273	-7.971208	-0.136963
H	-5.007842	-6.528864	0.488052
H	-5.172991	-5.520437	-1.866194
H	-5.841856	-7.027580	-2.420323
H	-3.565575	-7.032322	-3.058802
H	-3.633097	-8.164669	-1.716699
H	-2.571007	-6.524582	-0.217094
H	-2.820414	-5.226123	-1.355007
H	-1.246975	-6.320698	-2.844191
H	0.073076	-5.739556	-1.447025
H	-1.044538	-7.538764	-1.781477
H	-7.774975	-6.475431	-0.821892
H	-6.606009	-4.373594	0.880042
H	4.452928	1.617966	4.559886
H	6.318440	2.430275	3.084903
H	4.737154	2.554616	2.329164
H	5.535372	4.008696	2.972092
H	5.929981	4.088734	5.630956
H	5.191448	2.825671	6.631862
H	6.570717	2.440438	5.589541
H	3.580956	4.544480	4.599883
H	-1.014113	4.439686	1.282288
H	-0.628659	4.177360	3.011273
H	-0.014715	5.604193	2.132649
H	0.817288	2.682738	1.700796
H	2.463928	4.894011	2.741021
H	2.950930	2.539370	-2.210075
H	2.817170	4.319109	-2.059017
H	2.263212	2.239331	-0.060817
H	-1.707469	2.541411	-5.655133
H	-1.413688	2.189819	-3.961201
H	0.573165	0.842943	-4.509892
H	0.428143	1.272270	-6.225372
H	-1.900427	0.254810	-6.216055
H	-1.660483	-0.261036	-4.559307
H	-0.123889	-1.250024	-6.907237
H	-0.501629	-1.725349	-3.423652
H	-0.517498	-3.425501	-3.613279
H	0.159107	-3.545597	-6.921320
H	0.348579	-4.308371	-5.298810
H	0.261060	3.928272	-5.835688
H	1.877952	4.126569	-4.288579
H	-4.400687	4.128187	-3.409644
H	-4.031360	5.236704	-4.749772
H	-5.861446	5.507862	-2.721417
H	-2.637678	6.620035	-3.318554
H	-1.438279	4.068639	-2.657406
H	-3.191266	6.346213	1.009616
H	-4.314507	7.095674	-1.639766
H	-6.041623	2.679943	1.020955
H	-6.960969	4.093129	1.557734
H	-4.054702	4.129760	0.902627
H	-2.351288	1.890491	3.832853
H	-3.755518	0.831968	3.732526
H	-2.198223	1.330712	6.307410
H	-3.368533	-0.741558	7.091761
H	-4.254453	-0.825991	5.563592
H	-4.544845	0.513354	6.680632
H	-1.137358	-0.918503	5.855760
H	-0.728179	0.250436	4.597901
H	-1.964341	-0.982087	4.308856
H	-5.015066	2.298480	5.307435
H	-3.847638	3.708782	2.994985
H	7.562137	4.200292	0.233402
H	7.445234	4.551393	-1.496158
H	5.498980	3.090005	-0.104417
H	5.121242	4.046287	-1.533503
H	3.927071	5.602013	-0.311411
H	4.172442	4.635351	1.150332
H	7.647189	6.584831	0.334989
H	1.882113	-1.524821	-0.198126
H	3.261203	0.168334	0.908223
H	3.888173	-1.354240	-1.459882
H	4.531658	0.719849	-1.198454
H	1.957844	-2.470346	-4.371344

H	3.432852	-2.671106	-3.412818
H	2.662458	-0.282198	-3.727651
H	0.890368	1.075142	-1.395217
H	-0.576286	-2.467998	-0.973570
H	-0.422637	2.313914	-0.548632
H	-6.857133	5.002453	-0.776133
H	-7.647444	4.210836	-1.861885
H	-2.110264	-0.107414	1.989826
C	5.108002	7.329346	1.288346
H	4.410311	7.133768	2.110463
H	5.903529	7.975664	1.675623
H	4.550133	7.882732	0.510706
C	7.101239	8.351919	-1.519197
H	6.741023	8.872866	-2.407200
H	6.932552	8.971919	-0.630665
H	8.183519	8.191195	-1.590311
H	-3.594806	3.343883	5.474258
H	2.934569	3.259870	5.635505
H	9.016523	-0.885304	1.429462
H	3.048580	-1.640349	6.280647
H	3.863481	-1.797681	4.707518
H	-4.347468	7.668196	0.704050
H	-3.267823	-4.395187	3.866840
H	-7.479391	-6.547117	0.930551
H	6.948966	-6.734997	2.356041
H	-1.611158	2.347405	0.676069

INT1'

C	2.832250	-1.451478	5.306983
C	1.571154	-1.836645	4.591291
O	0.903310	-2.829276	4.865406
N	1.301181	-1.059815	3.503807
C	0.459175	-1.601908	2.477765
C	0.980157	-2.943347	1.952029
O	0.223891	-3.777381	1.456707
N	2.324953	-3.110107	2.038987
C	2.938033	-4.352280	1.646697
C	4.306118	-4.514351	2.300920
O	4.906400	-3.589845	2.847264
C	3.104858	-4.354719	0.123113
O	3.611197	-5.613903	-0.318655
N	4.830438	-5.765736	2.133250
C	6.255831	-5.987557	2.263355
C	7.129830	-5.510564	1.089314
O	8.351388	-5.580067	1.181048
N	6.477413	-4.967218	0.026166
C	7.213430	-4.346201	-1.058835
C	7.728931	-2.917389	-0.750990
O	8.396185	-2.331744	-1.600819
C	6.408775	-4.382911	-2.363000
O	5.123048	-3.789863	-2.210816
N	7.455551	-2.430534	0.479210
C	7.941987	-1.144637	0.969058
C	6.850565	-0.499712	1.834469
C	5.574313	-0.133828	1.054434
O	5.734310	0.414435	-0.069798
O	4.461701	-0.377818	1.621605
C	-3.802423	-3.595085	3.055517
C	-5.278287	-3.233437	3.044043
O	-6.160848	-4.095004	2.957233
C	-3.421203	-4.233595	1.714232
O	-3.845640	-3.428412	0.617186
N	-5.529248	-1.900173	3.072800
C	-6.846073	-1.334552	2.824228
C	-7.193199	-1.243206	1.322975
O	-8.180327	-0.608818	0.946175
C	-6.977640	0.021366	3.509589
N	-6.411640	-1.980964	0.503349
C	-6.741299	-2.247283	-0.874114
C	-7.175959	-3.714228	-1.095549
O	-7.493421	-4.093274	-2.224915
C	-5.563515	-1.878554	-1.800426
C	-5.219352	-0.415189	-1.756099
N	-3.942190	0.058462	-2.032786
C	-6.055119	0.646055	-1.486136
C	-4.023473	1.377920	-1.940301
N	-5.280908	1.782450	-1.609785
N	-7.180148	-4.511892	0.002290
C	-7.408982	-5.953644	-0.047732
C	-6.095245	-6.722402	-0.300754
C	-5.566801	-6.539212	-1.731271
C	-4.143319	-7.046522	-2.001802
C	-3.028306	-6.186928	-1.386174
N	-1.695681	-6.437381	-1.978383
C	3.372357	3.312599	4.719746
C	2.402808	2.870233	3.637642
O	1.874827	1.757715	3.669824
C	4.678618	2.490520	4.676445
C	5.411457	2.671453	3.342985
C	5.571908	2.860360	5.863761
N	2.170688	3.755259	2.628147
C	1.127421	3.534237	1.627851
C	1.594581	4.000605	0.235668
O	1.377042	5.141528	-0.168553
C	-0.152588	4.274390	2.026901
N	2.222098	3.066074	-0.525295
C	2.550736	3.361677	-1.899090
C	1.307359	3.367999	-2.789164
O	0.242936	2.830806	-2.440161
N	1.413023	3.906118	-4.017297
C	0.319346	3.957943	-5.005429
C	-0.760135	5.029265	-4.690237
O	-0.992522	5.943044	-5.475271
C	-0.421800	2.626946	-5.255635
C	0.407040	1.366436	-5.501366
C	-0.566718	0.222372	-5.811814
N	0.083007	-1.041779	-6.157645
C	-0.103589	-2.222682	-5.535513
N	-0.678562	-2.280732	-4.326274
N	0.269263	-3.366276	-6.114488
N	-1.454247	4.796660	-3.552138
C	-2.550444	5.617978	-3.098142
C	-2.730052	5.347696	-1.605936
O	-2.188443	4.386515	-1.049908
C	-3.859645	5.285672	-3.862832
O	-4.922504	6.125464	-3.447045
N	-3.531239	6.228116	-0.966165
C	-3.895789	6.010295	0.422090
C	-5.217179	5.257001	0.563305
O	-6.286451	5.846331	0.311052
N	-5.134013	3.974092	0.934557
C	-6.211547	3.256636	1.609843
C	-5.955454	3.228495	3.136697
O	-6.857980	3.395789	3.948503
N	-4.655496	3.002896	3.449505
C	-4.148173	2.798005	4.795853
C	-3.002853	1.779189	4.717590
C	-2.228780	1.530544	6.018035
C	-3.118371	0.901222	7.097285
C	-0.998765	0.655597	5.737719
N	5.850458	5.823759	0.951712
C	6.955398	5.877695	-0.014054
C	6.600366	6.723621	-1.249507
O	5.718154	6.386339	-2.022705
C	7.135322	4.391960	-0.364281
C	5.695418	3.830263	-0.369028
C	4.869479	4.852925	0.439708
P	1.324769	-4.779066	-2.783913
O	0.392163	-4.923797	-3.994527
O	0.543974	-5.160848	-1.459128
O	2.714472	-5.390039	-2.842355
O	1.513573	-3.156276	-2.492179
C	2.054293	-0.584602	-0.584513
O	2.287536	0.279005	0.495080
C	3.165984	-0.656345	-1.652553
O	3.814181	0.582767	-1.804764
C	2.382567	-2.433730	-3.359114
C	2.378003	-0.976744	-2.935958
O	0.994287	-0.548399	-2.711433
C	0.895447	-0.017726	-1.403191
N	-0.412764	-0.265459	-0.803887
C	-1.091815	0.695186	-0.127541
C	-2.019443	-0.004405	0.807906
N	-1.981506	-1.397981	0.370905
C	-1.016192	-1.505769	-0.461801
N	-0.964229	1.979731	-0.219718
C	-3.339380	0.738346	1.161476
O	-3.164762	2.003887	1.209552
O	-4.324995	0.084118	1.495266
O	-6.577085	4.469243	-1.959550
H	2.993525	-0.369919	5.287301
H	0.458938	-0.884426	1.658752
H	-0.563025	-1.787003	2.818460
H	1.792235	-0.188050	3.345549
H	3.786104	-3.544346	-0.163942
H	2.118269	-4.165673	-0.287770
H	3.263214	-5.745560	-1.234375
H	2.272434	-5.177922	1.917259
H	2.935414	-2.375023	2.378820
H	6.613299	-5.444350	3.140060
H	4.332819	-6.341871	1.462103
H	6.313040	-5.425572	-2.689223
H	6.988635	-3.830119	-3.109516
H	4.428258	-4.434244	-2.441544
H	8.135416	-4.916790	-1.221329
H	5.476200	-5.114627	-0.102637
H	7.247275	0.427972	2.264333
H	6.572480	-1.154186	2.666106
H	8.158679	-0.523279	0.099068
H	6.851562	-2.968944	1.085540
H	-3.939087	-5.191985	1.620599
H	-2.338144	-4.400855	1.676861
H	-3.213771	-2.675332	0.552199
H	-3.179641	-2.713428	3.240451
H	-6.145949	0.659630	3.208053
H	-7.910020	0.501926	3.213236
H	-6.955255	-0.088045	4.596690
H	-7.574407	-2.045547	3.229334
H	-4.762475	-1.272872	2.850313
H	-5.830486	-2.191741	-2.815699
H	-4.684910	-2.461713	-1.507467
H	-7.081660	0.681199	-1.155430
H	-5.612116	2.740752	-1.525501
H	-3.210287	2.079329	-2.064690
H	-7.614808	-1.637901	-1.121858
H	-5.509957	-2.329576	0.815703
H	-6.255007	-7.791050	-0.098224
H	-5.360319	-6.370988	0.432521
H	-5.610574	-5.483353	-2.004127
H	-6.259108	-7.040571	-2.418796
H	-3.991702	-7.054248	-3.090514
H	-4.028073	-8.089401	-1.670888
H	-2.945500	-6.356586	-0.309412
H	-3.254888	-5.123015	-1.503756
H	-1.706401	-6.244764	-2.980374
H	-0.368446	-5.614008	-1.590261
H	-1.454535	-7.423114	-1.885940
H	-8.131312	-6.154673	-0.844381
H	-6.866937	-4.131536	0.889744
H	4.397536	1.434220	4.762321
H	6.362237	2.131761	3.358074
H	4.827138	2.271085	2.511124
H	5.634207	3.726309	3.140502
H	5.881612	3.911272	5.808024
H	5.055038	2.711973	6.818157

H	6.478766	2.247550	5.872368
H	3.608266	4.380954	4.631756
H	-0.913242	4.204742	1.248554
H	-0.544619	3.857601	2.958296
H	0.063921	5.336732	2.165966
H	0.968457	2.455147	1.611744
H	2.547049	4.688885	2.706964
H	3.206101	2.557367	-2.249792
H	3.066490	4.323284	-1.983380
H	2.405628	2.122288	-0.166721
H	-1.060674	2.820215	-6.127232
H	-1.092543	2.430690	-4.419458
H	0.987893	1.106666	-4.612930
H	1.104725	1.507171	-6.337704
H	-1.219054	0.507980	-6.647007
H	-1.219438	0.042106	-4.955822
H	0.598349	-1.067518	-7.024871
H	-0.423705	-1.539394	-3.674242
H	-0.624654	-3.221504	-3.923506
H	0.616424	-3.375539	-7.059459
H	0.419144	-4.173193	-5.456508
H	0.783500	4.293049	-5.934543
H	2.250391	4.428909	-4.231922
H	-4.095682	4.224587	-3.708111
H	-3.655421	5.443276	-4.927077
H	-5.583556	5.567516	-2.985655
H	-2.326281	6.676218	-3.267597
H	-1.138517	4.074789	-2.907226
H	-3.074481	5.481003	0.903995
H	-4.140105	6.786784	-1.556797
H	-6.265601	2.230830	1.232635
H	-7.155456	3.767622	1.432098
H	-4.232099	3.497246	0.934882
H	-2.296143	2.123740	3.949913
H	-3.411083	0.828114	4.348722
H	-1.875864	2.504851	6.390281
H	-2.551141	0.717831	8.015837
H	-3.513964	-0.061704	6.752892
H	-3.970287	1.539454	7.351922
H	-0.421237	0.480758	6.651774
H	-0.328048	1.113049	5.003721
H	-1.293275	-0.327657	5.351921
H	-4.969778	2.450070	5.428491
H	-4.046681	2.683404	2.697172
H	7.729752	3.922566	0.423971
H	7.653128	4.239590	-1.314634
H	5.660573	2.822028	0.047074
H	5.321500	3.776439	-1.391555
H	4.115261	5.342788	-0.192380
H	4.347676	4.393081	1.282555
H	7.839067	6.308645	0.473430
H	1.813307	-1.605182	-0.259770
H	3.189287	0.031387	0.943059
H	3.889638	-1.448943	-1.432921
H	4.555649	0.595499	-1.118101
H	2.045161	-2.526732	-4.396442
H	3.407712	-2.810459	-3.307429
H	2.795348	-0.365088	-3.742411
H	0.991864	1.061517	-1.444507
H	-0.638722	-2.428866	-0.887326
H	-0.404281	2.427627	-0.958093
H	-6.702415	4.982691	-1.121480
H	-7.458459	4.248193	-2.284381
H	-1.506929	0.014035	1.784674
C	5.288062	7.095799	1.362973
H	4.575245	6.928469	2.178128
H	6.080341	7.748868	1.745248
H	4.747209	7.628112	0.559414
C	7.347093	8.032357	-1.427056
H	7.008797	8.540480	-2.330968
H	7.178035	8.675049	-0.554891
H	8.426987	7.850079	-1.479266
H	-3.785921	3.744222	5.223523
H	2.867576	3.157508	5.678732
H	8.863620	-1.280301	1.549727
H	2.807388	-1.809954	6.337356
H	3.670270	-1.931884	4.787850
H	-4.036070	6.977935	0.910348
H	-3.616659	-4.308528	3.865214
H	-7.845363	-6.253338	0.908772
H	6.452283	-7.049997	2.428606
H	-1.714216	2.479551	0.275993

E:ylide

C	2.919275	-1.795253	5.302530
C	1.567927	-2.082340	4.714971
O	0.776754	-2.900099	5.176188
N	1.314373	-1.419012	3.544072
C	0.263995	-1.923475	2.704988
C	0.572028	-3.294697	2.112734
O	-0.336437	-4.000949	1.627310
N	1.855465	-3.678429	2.086175
C	2.260859	-4.878991	1.391714
C	3.626626	-5.352563	1.889593
O	4.345330	-4.683035	2.623715
C	2.327884	-4.568703	-0.112583
O	2.620303	-5.756453	-0.844384
N	3.977369	-6.572384	1.373986
C	5.363485	-6.989523	1.341990
C	6.208163	-6.467288	0.166193
O	7.389736	-6.788341	0.086440
N	5.595234	-5.605079	-0.689938
C	6.339952	-4.946527	-1.748051
C	7.155536	-3.715165	-1.280609
O	7.834259	-3.098589	-2.098233
C	5.421031	-4.590673	-2.923313
O	4.321968	-3.792388	-2.505483
N	7.104090	-3.440411	0.042738
C	7.738706	-2.280848	0.659477
C	6.704819	-1.603003	1.587194
C	5.424957	-1.165743	0.842996
O	5.569352	-0.254884	-0.023306
O	4.337099	-1.744580	1.140380
C	-4.092975	-2.881760	3.477325
C	-5.505160	-2.444748	3.156370
O	-6.379837	-3.270087	2.862382
C	-3.555274	-4.066132	2.680078
O	-2.981589	-3.654583	1.445080
N	-5.770112	-1.119865	3.286587
C	-7.147907	-0.639678	3.319198
C	-7.754891	-0.347008	1.936372
O	-8.882595	0.129887	1.848670
C	-7.334457	0.550514	4.258935
N	-7.011233	-0.720298	0.861426
C	-7.620738	-0.994804	-0.430847
C	-8.183295	-2.439170	-0.520330
O	-8.734720	-2.813652	-1.554360
C	-6.631557	-0.738823	-1.574651
C	-6.249859	0.701454	-1.740981
N	-5.451190	1.107651	-2.797382
C	-6.568626	1.787247	-0.954554
C	-5.293746	2.409982	-2.637189
N	-5.951106	2.874822	-1.540435
N	-8.006898	-3.207724	0.580051
C	-8.204891	-4.649402	0.619465
C	-6.916074	-5.388768	0.194957
C	-6.581456	-5.228233	-1.297179
C	-5.209866	-5.778064	-1.733461
C	-4.026621	-4.831258	-1.480004
N	-2.742248	-5.326979	-2.026461
C	4.153659	2.827886	4.642365
C	3.336144	2.070835	3.610065
O	3.264638	0.838444	3.631743
C	5.592148	2.272647	4.702724
C	6.288612	2.331351	3.336292
C	6.399823	3.009132	5.774982
N	2.736994	2.802687	2.643151
C	1.964393	2.163154	1.581655
C	1.938975	3.145026	0.407483
O	1.485290	4.288416	0.548146
C	0.555487	1.801564	2.064934
N	2.476062	2.666902	-0.732202
C	2.514115	3.428856	-1.950406
C	1.194088	3.398285	-2.706243
O	0.127430	3.198482	-2.133201
N	1.248434	3.694852	-4.040550
C	0.075351	4.320666	-4.659942
C	-0.299731	5.547046	-3.768338
O	0.574414	6.159083	-3.171756
C	-1.109007	3.364779	-4.901510
C	-0.767724	1.971402	-5.430896
C	-2.071518	1.210997	-5.724427
N	-1.869441	-0.167332	-6.166661
C	-1.540640	-1.192665	-5.345633
N	-1.600786	-1.042116	-4.030453
N	-1.180332	-2.367917	-5.873109
N	-1.629454	5.861452	-3.667790
C	-2.124926	6.392830	-2.389370
C	-2.403991	5.157533	-1.527948
O	-3.315928	4.388814	-1.859813
C	-3.424263	7.182071	-2.598776
O	-3.979048	7.599287	-1.375110
N	-1.619332	4.920569	-0.465699
C	-1.760830	3.672205	0.255273
C	-3.106345	3.543796	0.946045
O	-3.659953	4.559779	1.442480
N	-3.592837	2.311710	1.098491
C	-4.751607	2.085191	1.949622
C	-4.302600	2.084688	3.418055
O	-4.106585	1.040172	4.054370
N	-4.097809	3.315979	3.942740
C	-3.336737	3.486090	5.180007
C	-1.836960	3.276082	4.918613
C	-0.917449	3.419971	6.140366
C	-1.260358	2.408768	7.242339
C	0.548033	3.264191	5.710016
N	6.661958	4.928192	0.655588
C	7.676974	4.787150	-0.396522
C	7.429811	5.747902	-1.574157
O	6.445816	5.636557	-2.286612
C	7.509096	3.313071	-0.805770
C	5.999662	3.021602	-0.621660
C	5.462674	4.199223	0.217685
P	0.433089	-4.177662	-3.235707
O	-0.516435	-4.479671	-4.381341
O	-0.310660	-4.302126	-1.827668
O	1.802357	-4.838955	-3.193280
O	0.676468	-2.533837	-3.207216
C	1.742036	-1.049834	-0.896132
O	2.045544	-0.752448	0.437966
C	2.890376	-0.829234	-1.901047
O	3.629393	0.340492	-1.601043
C	1.717568	-1.894644	-3.945665
C	2.113631	-0.620082	-3.202915
O	0.914694	0.102005	-2.822027
C	0.660775	-0.093802	-1.438705
N	-0.727364	-0.538085	-1.181396
C	-1.853615	0.320446	-1.226926
C	-2.840327	-0.090938	-0.354286
N	-2.261724	-1.277806	0.154832
C	-1.048032	-1.534737	-0.325985
N	-1.955167	1.266806	-2.263797
O	-5.534169	5.522649	-0.227296
H	3.246918	-0.773038	5.111732
H	0.117260	-1.234100	1.875429

H	-0.668007	-2.021914	3.266238
H	2.001957	-0.769554	3.173608
H	3.095382	-3.803413	-0.281795
H	1.350573	-4.187149	-0.403019
H	2.258082	-5.606030	-1.753898
H	1.502774	-5.650763	1.550402
H	2.598371	-3.048299	2.372307
H	5.848253	-6.624046	2.249701
H	3.384785	-6.892663	0.614170
H	5.065477	-5.520330	-3.384777
H	6.032936	-4.046324	-3.651178
H	3.482431	-4.198368	-2.803028
H	7.103880	-5.641038	-2.116375
H	4.579036	-5.533530	-0.717646
H	7.158626	-0.712785	2.037719
H	6.420298	-2.288317	2.391856
H	8.041683	-1.607893	-0.144802
H	6.408508	-3.931896	0.587723
H	-4.374750	-4.766599	2.485587
H	-2.796721	-4.581723	3.278688
H	-2.004471	-3.810920	1.500876
H	-3.395324	-2.039643	3.411203
H	-6.798723	1.434912	3.909570
H	-8.396835	0.793987	4.295270
H	-6.975846	0.309477	5.262202
H	-7.758272	-1.476261	3.681724
H	-5.032832	-0.463303	3.567812
H	-7.086633	-1.120897	-2.492400
H	-5.717407	-1.327944	-1.407825
H	-7.169538	1.874909	-0.063783
H	-5.885657	3.821184	-1.165699
H	-4.712435	3.066084	-3.265015
H	-8.479368	-0.325732	-0.528257
H	-6.070089	-1.052337	1.021578
H	-7.020369	-6.457169	0.431731
H	-6.099314	-5.008057	0.818707
H	-6.642068	-4.174177	-1.580997
H	-7.367869	-5.723671	-1.878893
H	-5.244090	-5.991212	-2.810906
H	-5.013430	-6.741280	-1.239086
H	-3.876431	-4.641045	-0.414006
H	-4.222532	-3.858577	-1.945693
H	-2.785165	-5.405066	-3.042552
H	-1.287300	-4.609761	-1.871768
H	-2.564242	-6.267969	-1.677899
H	-9.034929	-4.900920	-0.047046
H	-7.457971	-2.860681	1.357864
H	5.503746	1.217954	4.988353
H	7.300469	1.918535	3.408463
H	5.752142	1.743922	2.585510
H	6.382448	3.357906	2.962521
H	6.512473	4.069978	5.519448
H	5.915832	2.949986	6.756285
H	7.404273	2.583592	5.868216
H	4.171932	3.901641	4.416382
H	-0.041607	1.358966	1.263380
H	0.629273	1.076691	2.876970
H	0.044016	2.688965	2.443626
H	2.477147	1.244750	1.296289
H	2.721807	3.812056	2.681467
H	3.331154	3.051519	-2.569589
H	2.695148	4.487913	-1.728351
H	2.853854	1.708543	-0.807064
H	-1.782235	3.861899	-5.614939
H	-1.664180	3.241065	-3.970019
H	-0.183000	1.428136	-4.683298
H	-0.162307	2.031693	-6.344047
H	-2.633783	1.712856	-6.517583
H	-2.717924	1.213695	-4.840571
H	-1.955713	-0.373465	-7.148242
H	-1.825944	-0.136532	-3.596515
H	-1.003066	-1.655320	-3.477404

H	-0.967654	-2.417337	-6.856012
H	-0.949393	-3.206245	-5.269584
H	0.419283	4.703681	-5.626715
H	2.136314	4.060935	-4.356732
H	-4.120134	6.551744	-3.171700
H	-3.197919	8.069013	-3.200567
H	-4.514407	6.863095	-1.014767
H	-1.350431	7.028469	-1.961334
H	-2.269410	5.174882	-4.044770
H	-1.574881	2.816099	-0.389682
H	-0.739297	5.408753	-0.365124
H	-5.162430	1.112549	1.687710
H	-5.508004	2.855315	1.774347
H	-3.283917	1.451367	0.547182
H	-1.523568	3.988502	4.144055
H	-1.711238	2.272639	4.494440
H	-1.047378	4.433571	6.549753
H	-0.561064	2.493213	8.080792
H	-1.198071	1.384769	6.856787
H	-2.269742	2.555175	7.638396
H	1.226505	3.428493	6.554052
H	0.812287	3.974177	4.919378
H	0.734734	2.255894	5.323847
H	-3.711600	2.756030	5.899368
H	-4.072156	4.071291	3.257891
H	8.100950	2.694560	-0.126517
H	7.855904	3.119677	-1.823802
H	5.852277	2.054540	-0.138827
H	5.494542	2.986472	-1.587683
H	4.800388	4.837967	-0.387122
H	4.898498	3.872466	1.097479
H	8.665844	4.995754	0.031859
H	1.398621	-2.080262	-1.018847
H	2.961233	-1.132105	0.664456
H	3.553935	-1.697822	-1.955704
H	4.391596	0.084461	-0.971123
H	1.341958	-1.642961	-4.945112
H	2.583077	-2.555729	-4.046313
H	2.689629	0.026665	-3.871653
H	0.743386	0.876204	-0.946701
H	-0.450307	-2.403001	-0.116428
H	-1.204025	1.956895	-2.281204
H	-4.835605	5.202535	0.397942
H	-6.218760	5.906516	0.335234
H	-2.732231	-1.956528	0.758875
C	6.422476	6.271025	1.142707
H	5.753757	6.226307	2.008949
H	7.365006	6.722228	1.472422
H	5.953405	6.941535	0.398793
C	8.426368	6.879349	-1.775331
H	8.137381	7.484983	-2.635153
H	8.463418	7.504650	-0.875425
H	9.435234	6.475463	-1.920928
H	-3.537210	4.488828	5.569854
H	3.665822	2.702315	5.615447
H	8.627159	-2.580471	1.228759
H	2.906139	-2.014503	6.371729
H	3.637887	-2.472441	4.823533
H	-0.994854	3.673624	1.029460
H	-4.124007	-3.165356	4.538889
H	-8.475213	-4.922310	1.642437
H	5.431293	-8.080604	1.345699
H	-2.857985	1.730198	-2.188822

E:ATP:CAIR:Asp

C	-10.828393	-1.512455	-0.353533
C	-10.302329	-0.089251	-0.414196
O	-11.065214	0.871798	-0.493917
N	-8.945803	0.030939	-0.326485
C	-8.325627	1.337264	-0.449728
C	-7.908331	1.688907	-1.892136

O	-7.639535	2.856246	-2.169859
C	-7.172584	1.512572	0.561198
C	-7.666266	1.538402	2.029350
C	-6.789493	0.785557	3.054554
C	-5.379003	1.349870	3.245648
N	-4.527160	0.442789	4.091234
N	-7.925061	0.681060	-2.833483
C	-7.523993	0.985503	-4.200266
C	-8.454886	0.451836	-5.318219
O	-8.095513	0.556467	-6.490200
C	-6.052884	0.572421	-4.489661
O	-5.847216	-0.794480	-4.118273
C	-5.058560	1.498514	-3.796383
N	-9.658709	-0.090797	-4.970566
C	-10.448359	-0.899991	-5.924293
C	-10.002854	-2.378566	-5.817084
C	-8.538295	-2.644173	-6.230048
C	-8.001234	-4.023275	-5.776978
C	-7.241348	-4.006643	-4.440640
N	-5.801178	-3.639976	-4.601721
C	-4.315221	7.344014	0.352144
C	-3.092918	7.834435	1.104848
O	-2.056309	8.224725	0.548666
C	-4.227059	5.866180	-0.116205
O	-3.276025	5.767342	-1.167758
C	-5.588510	5.367711	-0.597203
N	-3.192340	7.829723	2.463645
C	-2.192878	8.460252	3.321957
C	-1.397054	7.496565	4.215611
O	-0.537536	7.949073	4.964600
C	-2.846157	9.521141	4.215962
N	-1.700378	6.172521	4.133548
C	-1.341826	5.310161	5.238258
C	-2.376243	5.411312	6.369412
O	-3.497919	5.854970	6.168579
N	-1.940770	4.934571	7.575344
C	-2.881141	4.327728	8.519798
C	-2.572274	2.839502	8.692816
C	-2.421290	2.075824	7.367114
O	-2.954680	2.444586	6.323601
N	-1.624284	0.974674	7.453245
C	4.068875	-5.651936	1.277500
C	2.597122	-5.420328	1.583455
C	2.312311	-4.369923	2.651231
O	1.094880	-4.181103	2.955746
O	3.289639	-3.732797	3.153775
C	10.095341	-5.613846	-0.919939
C	9.966218	-4.165858	-0.431690
C	11.059581	-3.284436	-1.051437
C	8.584012	-3.608235	-0.784589
C	6.189429	-5.682321	4.881148
C	6.930595	-4.435553	4.445086
C	6.950284	-4.050725	3.092885
C	7.603755	-3.620480	5.368553
C	7.613336	-2.894929	2.680859
C	8.263365	-2.455417	4.959073
C	8.269285	-2.082605	3.613121
C	-0.916017	-7.309450	4.578877
C	-0.243589	-7.517068	3.215128
C	-1.189306	-7.500209	1.996682
C	-1.813314	-6.146322	1.655901
N	-0.805603	-5.161482	1.188726
C	-3.943448	-1.320531	8.738618
C	-3.879824	-1.011527	7.199040
O	-2.848295	-1.397041	6.542145
O	-4.830209	-0.328593	6.745407
C	2.964741	-3.670482	8.280171
C	2.992195	-3.230571	6.826688
C	1.616633	-3.165382	6.174939
O	0.693219	-3.862702	6.611606
O	1.521542	-2.364260	5.148779
C	9.009516	-1.290245	-5.542396

C	8.454709	-2.453810	-4.721644
O	9.193418	-3.311936	-4.236239
N	7.101437	-2.458546	-4.608391
C	6.368688	-3.364995	-3.736717
C	5.708734	-2.571630	-2.583889
C	4.694030	-3.372650	-1.742203
C	3.227027	-3.328160	-2.246833
C	2.362269	-2.366534	-1.423818
N	0.948175	-2.323038	-1.906773
C	0.663827	-8.367733	-3.314620
C	1.170430	-7.041324	-2.735107
C	0.122204	-6.189119	-2.004499
O	-1.092371	-6.494141	-2.137098
O	0.555173	-5.216824	-1.296208
C	-2.223560	-6.472947	-5.989286
C	-3.217693	-5.353466	-5.598625
O	-4.456456	-5.565490	-5.796642
C	1.998728	3.916008	-5.647496
C	3.106248	3.380909	-4.766585
O	3.985664	4.153724	-4.334130
N	3.114066	2.061793	-4.492876
C	4.179863	1.491796	-3.674555
C	5.513558	1.303651	-4.413694
O	5.906059	0.188698	-4.769004
N	6.246275	2.423297	-4.589988
C	7.603177	2.364785	-5.086825
C	8.131965	3.782429	-5.295552
O	7.673029	4.741914	-4.683893
N	9.164261	3.875611	-6.184220
C	9.849132	5.123767	-6.490699
C	2.416311	7.462080	-4.333597
C	1.025704	7.634955	-4.954829
C	-0.105359	7.078063	-4.077994
C	-0.368522	7.939419	-2.839824
N	-1.066739	7.155933	-1.789028
C	4.297074	9.773603	1.275762
C	3.915144	8.691561	0.249457
C	3.410003	7.381347	0.884990
N	3.013710	6.374032	-0.097619
C	3.837751	5.517989	-0.738089
N	3.309718	4.668838	-1.621099
N	5.157742	5.492606	-0.506993
P	-1.758172	-1.645235	0.899677
O	-2.006800	-0.303334	1.552036
O	-1.299902	-1.757111	-0.573056
O	-1.064390	-2.663654	1.836057
P	-4.674228	-1.677134	1.056579
O	-4.809786	-1.669308	2.553205
O	-4.827466	-0.431820	0.215580
P	-6.163569	-3.030181	-1.049811
O	-7.247645	-2.064099	-1.454069
O	-4.899188	-3.173611	-1.899804
O	-5.735480	-2.826040	0.513251
O	-6.744383	-4.550381	-0.981405
C	-7.942485	-4.770964	-0.235648
C	1.768436	-0.682597	1.587985
C	8.541399	0.469400	-0.484136
C	8.293920	1.970650	-0.532379
O	-1.353387	-0.143661	-3.440698
C	-0.348878	0.618467	-3.336635
O	0.857766	0.277858	-3.515495
C	-0.657851	2.079142	-2.946339
C	0.330331	2.646859	-1.908804
C	-0.160756	4.019574	-1.420918
O	-1.269692	4.068897	-0.856949
O	0.589021	5.056793	-1.631871
N	0.504049	1.805101	-0.724003
O	1.518325	-1.260879	2.745818
C	3.171488	-0.264888	1.438882
N	4.144264	-0.820154	2.273115
C	5.314989	-0.481219	1.782464
N	5.164748	0.296142	0.661435
C	3.808635	0.473610	0.446921
N	3.329634	1.242986	-0.572702
C	6.232107	0.652562	-0.282567
O	7.308728	-0.196171	-0.012040
O	8.375646	2.395523	-1.970100
C	6.788829	2.094206	-0.179390
O	6.114878	2.968091	-1.107258
C	9.648687	0.096975	0.513087
O	9.660025	0.983411	1.611704
H	-10.105059	-2.231614	-0.749107
H	-11.041953	-1.778491	0.687226
H	-6.653666	2.443407	0.312229
H	-6.451600	0.706357	0.411428
H	-8.654825	1.067138	2.071257
H	-7.815620	2.576997	2.355731
H	-6.693071	-0.257864	2.744366
H	-7.301730	0.783594	4.025458
H	-5.395637	2.336301	3.718652
H	-4.866388	1.417969	2.282175
H	-3.595832	0.844700	4.211499
H	-4.462609	-0.471130	3.552145
H	-4.841083	0.222176	5.079164
H	-9.115705	2.062858	-0.235786
H	-8.354027	-0.794274	-0.435359
H	-5.927873	0.643510	-5.574348
H	-5.210328	2.531627	-4.120753
H	-5.184443	1.467483	-2.710769
H	-4.034406	1.195961	-4.032537
H	-5.208654	-0.841950	-3.379541
H	-7.572555	2.075395	-4.287425
H	-7.761965	-0.273271	-2.509095
H	-10.671059	-3.006974	-6.422379
H	-10.148724	-2.694712	-4.774098
H	-7.890763	-1.865604	-5.822607
H	-8.459827	-2.547826	-7.318290
H	-7.336191	-4.446937	-6.538147
H	-8.834845	-4.730407	-5.683381
H	-7.245507	-4.995756	-3.977110
H	-7.683080	-3.301683	-3.730110
H	-5.227851	-4.391582	-5.182303
H	-5.361403	-3.587198	-3.665088
H	-5.704155	-2.684052	-4.964726
H	-10.256216	-0.493184	-6.918778
H	-9.837264	-0.193762	-3.979602
H	-3.892698	5.251244	0.731900
H	-6.329193	5.378899	0.209215
H	-5.957008	5.996853	-1.413823
H	-5.515967	4.346515	-0.976907
H	-2.625415	5.046668	-0.983016
H	-5.209905	7.463578	0.972014
H	-3.589603	9.058992	4.875162
H	-2.081878	9.981340	4.842951
H	-3.334514	10.287187	3.607680
H	-1.473542	8.927842	2.646017
H	-4.093445	7.624654	2.874487
H	-0.345018	5.589932	5.584770
H	-1.327546	4.266514	4.908523
H	-2.546242	5.930246	3.638690
H	-3.384174	2.360688	9.254917
H	-1.661366	2.707986	9.291389
H	-1.813383	0.214964	6.793289
H	-1.390936	0.640658	8.377640
H	-3.875476	4.472796	8.094232
H	-1.009297	4.539211	7.574255
H	4.557991	-4.716137	0.998418
H	4.596543	-6.059865	2.144273
H	2.119764	-6.355657	1.896890
H	2.063514	-5.119430	0.671904
H	10.083864	-4.155497	0.662280
H	10.927824	-3.233773	-2.138239
H	11.013366	-2.263127	-0.659172
H	12.060183	-3.680050	-0.840885
H	8.451763	-2.582694	-0.427839
H	8.474442	-3.592865	-1.871537
H	7.778121	-4.221195	-0.362261
H	9.975360	-5.654963	-2.008808
H	6.214429	-5.802321	5.967520
H	5.142401	-5.636211	4.562107
H	6.426985	-4.654673	2.360939
H	7.598404	-3.894293	6.420393
H	7.603574	-2.615478	1.633580
H	8.769255	-1.838953	5.696392
H	8.768444	-1.173113	3.286764
H	-1.361709	-6.312550	4.659827
H	-1.704054	-8.051867	4.751321
H	0.283073	-8.479834	3.214852
H	0.528821	-6.750150	3.099483
H	-2.009080	-8.208993	2.169207
H	-0.653449	-7.863714	1.110152
H	-2.303881	-5.702307	2.526469
H	-2.552497	-6.232224	0.858730
H	-1.170497	-4.177951	1.248576
H	-0.010064	-5.078687	1.845401
H	-0.438885	-5.299548	0.221146
H	-3.553478	-0.449715	9.278522
H	-4.980697	-1.469391	9.043582
H	3.580231	-3.935980	6.222314
H	3.489031	-2.264378	6.719057
H	2.444248	-4.625294	8.384258
H	9.702447	-0.724905	-4.911855
H	5.210320	-1.695104	-3.016746
H	6.502459	-2.178618	-1.941117
H	4.721969	-2.995644	-0.711837
H	5.024650	-4.416031	-1.676821
H	2.762155	-4.316261	-2.173924
H	3.202005	-3.036718	-3.305403
H	2.736210	-1.341030	-1.441687
H	2.330049	-2.691549	-0.382680
H	0.262853	-1.942999	-1.195538
H	0.855166	-1.714831	-2.736361
H	0.595676	-3.274468	-2.096499
H	7.067585	-4.114877	-3.364075
H	6.609664	-1.613397	-4.878516
H	0.242651	-9.000868	-2.528062
H	-0.125889	-8.194270	-4.048999
H	1.585187	-6.410336	-3.533058
H	1.997771	-7.205101	-2.034401
H	-1.429693	-6.057086	-6.616519
H	-1.759508	-6.842681	-5.068292
H	2.445370	4.570700	-6.398290
H	4.370354	2.122492	-2.806710
H	2.256513	1.503576	-4.544731
H	8.241315	1.871911	-4.339386
H	5.859039	3.330166	-4.341572
H	9.361540	5.908349	-5.911091
H	9.466732	3.032793	-6.649061
H	2.489113	7.932468	-3.346028
H	2.685912	6.408680	-4.217183
H	1.000714	7.121611	-5.922689
H	0.837355	8.695485	-5.171487
H	0.165326	6.072618	-3.739911
H	-1.032313	6.988410	-4.656518
H	-0.963896	8.824759	-3.080156
H	0.571433	8.281114	-2.398513
H	-1.303059	7.689067	-0.928513
H	-1.985187	6.769072	-2.058670
H	-0.453629	6.316625	-1.547220
H	3.442632	10.041700	1.906094
H	5.102697	9.431395	1.934401
H	4.774115	8.463994	-0.393565
H	3.130233	9.070722	-0.416399
H	2.536114	7.577672	1.512942
H	4.169441	6.956958	1.554055
H	2.035687	6.285488	-0.353745

H	2.292163	4.739223	-1.790779
H	3.884616	4.338796	-2.395266
H	5.543058	6.084356	0.210262
H	5.654870	4.605844	-0.727234
H	-1.672497	2.133818	-2.546551
H	-0.629666	2.692999	-3.858833
H	1.307802	2.759333	-2.388574
H	0.534481	0.817974	-0.967527
H	-8.775956	-4.201718	-0.660272
H	-7.804514	-4.485544	0.812520
H	8.778594	0.070418	-1.481501
H	6.285358	-0.766052	2.147542
H	2.330329	1.501473	-0.481089
H	5.818213	0.496891	-1.291668
H	6.599899	2.469439	0.835782
H	6.795618	2.989062	-1.836335
H	10.608927	0.115970	-0.033301
H	9.508565	-0.923110	0.888482
Mg	3.111024	-1.856809	3.875368
Mg	-0.031242	-2.418428	3.543557
Mg	-2.860174	-2.681541	-1.609061
O	-3.280142	-2.409164	0.605618
O	4.951205	-1.907429	4.872256
H	5.505366	-2.692842	4.720793
H	5.502427	-1.156477	4.616206
O	2.695011	0.050818	4.895937
H	2.198701	0.446033	4.161179
H	1.995765	-0.372595	5.426925
O	-1.179609	-0.700748	4.051194
H	-1.905024	-0.892359	4.675643
H	-1.570255	-0.449091	3.156721
O	-2.757741	-4.292742	-5.099430
O	-3.608643	-0.778386	-2.116424
H	-3.992339	-0.416224	-1.272711
H	-2.842012	-0.246987	-2.432391
O	-2.706713	-4.705963	-1.223641
H	-3.601527	-5.039866	-1.373938
H	-2.054511	-5.381342	-1.622463
H	3.924909	2.027613	-0.812083
H	-0.293687	1.896555	-0.097345
O	0.952094	-0.539519	0.679741
H	11.075789	-6.036197	-0.671190
H	9.327946	-6.256001	-0.472238
H	1.478793	-8.914900	-3.800379
H	-2.728950	-7.290201	-6.506385
H	-8.161809	-5.839059	-0.295323
H	-11.509963	-0.790769	-5.685897
H	-11.761465	-1.567196	-0.916469
H	-2.845180	4.836437	9.489230
H	3.979620	-3.776046	8.677011
H	6.628654	-6.579283	4.430874
H	-3.333981	-2.190489	8.993393
H	5.597146	-3.886612	-4.319968
O	-1.263302	-3.430586	4.919830
H	-0.632551	-3.708305	5.639780
H	-1.955846	-2.916353	5.378126
H	8.227180	-0.621698	-5.906360
H	9.771308	5.362988	-7.556502
H	7.648190	1.781108	-6.012841
H	4.179982	-6.357144	0.447993
H	8.985334	3.138509	-1.958998
H	10.907515	5.075917	-6.212224
H	9.453834	1.842362	1.153835
H	2.433194	-2.941330	8.899837
H	4.640488	10.679072	0.768684
H	3.183098	7.923403	-4.963609
H	3.841371	0.519501	-3.326024
H	1.425018	3.125388	-6.135414
H	1.320491	4.517910	-5.035383
H	-4.436325	7.970637	-0.536389
H	9.581159	-1.691954	-6.383689
H	-0.182634	-7.398276	5.386026
O	-1.684845	-2.801693	-3.270330
H	-1.981400	-3.388567	-4.020978
H	-1.495641	-1.887773	-3.594041

TS3

C	-10.776819	-2.120722	-0.519910
C	-10.360930	-0.662281	-0.575456
O	-11.190895	0.244014	-0.631622
N	-9.018323	-0.456192	-0.510474
C	-8.472924	0.883572	-0.584808
C	-8.105338	1.316641	-2.015506
O	-7.946816	2.508619	-2.267902
C	-7.310149	1.046030	0.414665
C	-7.816626	0.983745	1.873548
C	-6.831103	0.403964	2.905145
C	-5.564932	1.235276	3.105769
N	-4.563720	0.512509	3.957481
N	-8.034314	0.328543	-2.975884
C	-7.647569	0.687841	-4.331686
C	-8.510896	0.072084	-5.466255
O	-8.136599	0.195089	-6.631077
C	-6.135478	0.423883	-4.590925
O	-5.790563	-0.898837	-4.162624
C	-5.243535	1.459921	-3.914650
N	-9.679844	-0.558430	-5.136519
C	-10.352495	-1.478628	-6.084141
C	-9.748426	-2.898131	-5.908765
C	-8.229424	-2.993236	-6.180722
C	-7.575861	-4.323509	-5.724354
C	-6.851562	-4.257829	-4.369463
N	-5.473150	-3.683293	-4.461554
C	-4.701628	7.033418	0.286547
C	-3.591109	7.637583	1.127779
O	-2.498518	8.002692	0.672737
C	-4.475811	5.534005	-0.052693
O	-3.428907	5.431233	-1.009897
C	-5.747987	4.896316	-0.607529
N	-3.859010	7.764803	2.455670
C	-2.953341	8.457916	3.368111
C	-1.913710	7.565911	4.066167
O	-0.880597	8.063548	4.499152
C	-3.762217	9.153900	4.470894
N	-2.268678	6.263335	4.244603
C	-1.716268	5.498057	5.338864
C	-2.721989	5.439138	6.500249
O	-3.836855	5.936271	6.407662
N	-2.273104	4.777256	7.615824
C	-3.234275	4.070576	8.467895
C	-3.276618	2.540010	8.140547
C	-2.827551	2.195185	6.713897
O	-3.313952	2.732816	5.717674
N	-1.772766	1.330340	6.625993
C	4.277307	-5.551901	1.303273
C	2.804739	-5.397229	1.685221
C	2.463468	-4.275744	2.662135
O	1.237846	-4.119631	2.955190
O	3.405245	-3.554622	3.125287
C	10.353779	-5.212887	-0.828954
C	10.119553	-3.783743	-0.324703
C	11.133876	-2.815362	-0.948813
C	8.693026	-3.331844	-0.655417
C	6.348976	-5.489495	4.934882
C	7.041582	-4.209607	4.510973
C	6.992110	-3.775355	3.173955
C	7.731780	-3.408974	5.434840
C	7.598966	-2.582248	2.777862
C	8.337250	-2.210351	5.042108
C	8.270209	-1.785911	3.712282
C	-0.666536	-7.451458	4.535230
C	0.081958	-7.360186	3.201648
C	-0.803300	-7.450840	1.943260
C	-1.672313	-6.225073	1.653679
N	-0.854764	-5.067153	1.204658
C	-4.029894	-1.622220	8.663605
C	-3.771344	-1.127467	7.190920
O	-2.748606	-1.572589	6.568476
O	-4.583777	-0.267329	6.760121
C	2.987588	-3.640529	8.293731
C	3.072139	-3.272559	6.822288
C	1.712634	-3.239713	6.139630
O	0.831826	-4.034636	6.484231
O	1.581213	-2.352739	5.186487
C	9.095178	-0.945534	-5.443193
C	8.580687	-2.130079	-4.625831
O	9.349940	-2.953277	-4.126576
N	7.227492	-2.196822	-4.530111
C	6.532217	-3.147304	-3.676158
C	5.771869	-2.400340	-2.558365
C	4.905989	-3.305695	-1.660682
C	3.451617	-3.551925	-2.126878
C	2.473075	-2.483751	-1.622983
N	1.045413	-2.865059	-1.852425
C	1.066299	-8.416127	-3.338110
C	1.500223	-7.302321	-2.379422
C	0.391017	-6.366236	-1.881909
O	-0.772160	-6.490047	-2.310064
O	0.763513	-5.479048	-1.014325
C	-1.872140	-6.654709	-6.048128
C	-2.625547	-5.420991	-5.492343
O	-3.894281	-5.424606	-5.603377
C	1.847018	3.922245	-5.633662
C	2.991673	3.474751	-4.756572
O	3.846152	4.293476	-4.348165
N	3.042027	2.163276	-4.479264
C	4.128754	1.625257	-3.675355
C	5.462168	1.471376	-4.417766
O	5.907474	0.366416	-4.740943
N	6.137583	2.618693	-4.631473
C	7.498982	2.602716	-5.114903
C	8.025787	4.031676	-5.198177
O	7.634149	4.912824	-4.438984
N	8.978347	4.227155	-6.155197
C	9.641687	5.503187	-6.370113
C	2.078241	7.481230	-4.308729
C	0.646534	7.539221	-4.855209
C	-0.394838	6.913192	-3.914886
C	-0.688041	7.786189	-2.691677
N	-1.340398	6.991817	-1.619201
C	3.772560	9.867068	1.328862
C	3.461736	8.763125	0.301519
C	3.081230	7.410064	0.934470
N	2.747009	6.384202	-0.051985
C	3.621818	5.606089	-0.721983
N	3.145070	4.741254	-1.619994
N	4.945300	5.668940	-0.510721
P	-1.038084	-1.331684	0.864843
O	-1.584732	-0.097258	1.526794
O	-0.929037	-1.520458	-0.663064
O	-0.637308	-2.507311	1.758604
P	-4.241850	-1.597921	1.044893
O	-4.303785	-1.617205	2.564574
O	-4.479915	-0.283053	0.315373
P	-5.885908	-3.062841	-0.925253
O	-7.039370	-2.249441	-1.468071
O	-4.630419	-3.204904	-1.800050
O	-5.506379	-2.631534	0.581312
O	-6.349813	-4.613217	-0.699945
C	-7.456205	-4.848204	0.169389
C	1.749133	-0.648247	1.695498
C	8.442300	0.742086	-0.433115
C	8.140220	2.224765	-0.545137
O	-1.384891	0.080211	-3.602544
C	-0.459228	0.941973	-3.702663

O	0.670596	0.755196	-4.224380
C	-0.766347	2.319618	-3.075119
C	0.203863	2.626913	-1.911867
C	-0.259105	3.922838	-1.228821
O	-1.299409	3.891931	-0.548344
O	0.427945	5.001379	-1.453527
N	0.289954	1.556615	-0.914474
O	1.521526	-1.115977	2.883772
C	3.106158	-0.192909	1.461115
N	4.114649	-0.567683	2.352480
C	5.248870	-0.172176	1.822733
N	5.040481	0.460985	0.620788
C	3.675913	0.483511	0.383432
N	3.109827	1.068734	-0.702407
C	6.107963	0.844060	-0.319833
O	7.201389	0.021879	-0.033450
O	8.203087	2.584639	-2.002160
C	6.630740	2.302653	-0.206512
O	5.944504	3.161427	-1.132634
C	9.496408	0.428891	0.636724
O	9.423522	1.325328	1.728533
H	-10.017603	-2.778807	-0.953582
H	-10.929949	-2.418117	0.523188
H	-6.816710	2.002101	0.214647
H	-6.558497	0.272836	0.233214
H	-8.706045	0.344260	1.898079
H	-8.153495	1.980724	2.191650
H	-6.533681	-0.600673	2.593113
H	-7.344336	0.299478	3.870506
H	-5.781055	2.202424	3.570328
H	-5.068910	1.389617	2.143914
H	-3.741189	1.104123	4.090042
H	-4.326072	-0.406102	3.405515
H	-4.852856	0.255158	4.925196
H	-9.287507	1.562786	-0.316744
H	-8.363251	-1.236119	-0.611288
H	-5.997839	0.463631	-5.675260
H	-5.486735	2.466460	-4.265465
H	-5.377599	1.441899	-2.829390
H	-4.193173	1.246049	-4.133064
H	-5.205074	-0.854205	-3.381501
H	-7.803182	1.768595	-4.409663
H	-7.769021	-0.604234	-2.656734
H	-10.282143	-3.603157	-6.561236
H	-9.950374	-3.222923	-4.877680
H	-7.717070	-2.172328	-5.676291
H	-8.054657	-2.832115	-7.249459
H	-6.856780	-4.675274	-6.472934
H	-8.340228	-5.107550	-5.654266
H	-6.729277	-5.259252	-3.949882
H	-7.404304	-3.654133	-3.643270
H	-4.762184	-4.339490	-5.001724
H	-5.100588	-3.571262	-3.496474
H	-5.503783	-2.720011	-4.821138
H	-10.170066	-1.086574	-7.086132
H	-9.855363	-0.675542	-4.146007
H	-4.177355	5.011698	0.867376
H	-6.560697	4.908523	0.125836
H	-6.083383	5.425493	-1.504875
H	-5.559956	3.858122	-0.888919
H	-2.757195	4.762517	-0.734900
H	-5.663964	7.139294	0.797469
H	-4.270797	8.411267	5.095488
H	-3.083732	9.720253	5.111425
H	-4.498126	9.838874	4.039281
H	-2.387516	9.183162	2.780817
H	-4.792533	7.555351	2.779912
H	-0.780906	5.974134	5.643979
H	-1.513342	4.469371	5.024194
H	-3.217067	6.015414	3.998404
H	-4.295678	2.154549	8.239748
H	-2.656227	1.991579	8.856369
H	-1.595248	0.950469	5.699590
H	-1.693078	0.612197	7.335335
H	-4.198988	4.538381	8.273015
H	-1.377939	4.315208	7.507324
H	4.689007	-4.608490	0.938296
H	4.879215	-5.874632	2.157010
H	2.431283	-6.328355	2.125690
H	2.192169	-5.250831	0.786958
H	10.249287	-3.773949	0.767944
H	10.986024	-2.765717	-2.033515
H	11.014042	-1.804119	-0.546173
H	12.164372	-3.134388	-0.752816
H	8.485995	-2.321195	-0.292615
H	8.568571	-3.319537	-1.740683
H	7.943148	-4.007291	-0.225461
H	10.226045	-5.252398	-1.916972
H	6.386767	-5.622552	6.019239
H	5.298769	-5.478715	4.623599
H	6.456002	-4.367268	2.442446
H	7.778609	-3.719952	6.475152
H	7.535565	-2.265200	1.742946
H	8.857678	-1.606219	5.779500
H	8.728755	-0.850171	3.401266
H	-1.352166	-6.607601	4.666404
H	-1.247787	-8.378226	4.608108
H	0.822287	-8.168793	3.148848
H	0.645556	-6.423448	3.193314
H	-1.469128	-8.318453	2.029999
H	-0.173397	-7.634035	1.063650
H	-2.232095	-5.904288	2.535848
H	-2.382207	-6.424731	0.848937
H	-1.415735	-4.209799	1.103965
H	-0.113693	-4.812642	1.881661
H	-0.377766	-5.241487	0.285284
H	-3.812374	-0.799383	9.353859
H	-5.086463	-1.876230	8.775890
H	3.674164	-4.012246	6.275966
H	3.575452	-2.313960	6.690747
H	2.456566	-4.586287	8.422506
H	9.763211	-0.354849	-4.809202
H	5.150436	-1.623745	-3.022060
H	6.507465	-1.871303	-1.943512
H	4.875905	-2.874703	-0.651682
H	5.407953	-4.274160	-1.548180
H	3.102719	-4.515771	-1.740288
H	3.406428	-3.616655	-3.221964
H	2.636349	-1.501219	-2.072594
H	2.578942	-2.362396	-0.542407
H	0.369088	-2.252571	-1.309743
H	0.725898	-2.787737	-2.822978
H	0.872234	-3.857483	-1.548700
H	7.273854	-3.833767	-3.266262
H	6.697974	-1.377883	-4.810803
H	0.301055	-9.048314	-2.879080
H	0.635412	-7.996613	-4.250621
H	2.259926	-6.663097	-2.848772
H	1.988172	-7.715387	-1.487358
H	-1.068647	-6.324596	-6.713039
H	-1.410859	-7.170521	-5.199582
H	2.259599	4.365759	-6.544110
H	4.324078	2.270427	-2.815601
H	2.163134	1.607399	-4.533024
H	8.127625	2.054344	-4.399754
H	5.709571	3.506670	-4.378623
H	9.221127	6.208705	-5.652715
H	9.220352	3.449488	-6.750191
H	2.162364	7.961575	-3.326612
H	2.440469	6.454649	-4.205213
H	0.609523	7.011655	-5.814937
H	0.367943	8.579337	-5.073848
H	-0.027510	5.944281	-3.561390
H	-1.332911	6.727750	-4.451714
H	-1.328769	8.635284	-2.946492
H	0.237822	8.183658	-2.266806
H	-1.631184	7.527513	-0.776579
H	-2.220855	6.520705	-1.886088
H	-0.674694	6.205967	-1.348244
H	2.912023	10.056701	1.978895
H	4.618559	9.590025	1.967374
H	4.322857	8.612597	-0.361071
H	2.633324	9.079613	-0.344120
H	2.208135	7.527879	1.582956
H	3.888973	7.045292	1.582148
H	1.772347	6.212902	-0.286021
H	2.124705	4.757595	-1.774780
H	3.733894	4.464228	-2.404941
H	5.294678	6.255727	0.228920
H	5.482578	4.810897	-0.744280
H	-1.791375	2.333996	-2.698426
H	-0.663797	3.096772	-3.843795
H	1.198735	2.783241	-2.344596
H	0.039882	0.648657	-1.304971
H	-8.355773	-4.337993	-0.193347
H	-7.230393	-4.503072	1.183816
H	8.760847	0.330895	-1.401066
H	6.241139	-0.324176	2.210615
H	2.069864	1.191476	-0.658451
H	5.703076	0.672532	-1.328638
H	6.425273	2.660974	0.811713
H	6.621052	3.184881	-1.865258
H	10.485351	0.474471	0.147857
H	9.372845	-0.588196	1.024233
Mg	3.178571	-1.726397	3.946678
Mg	0.050852	-2.418417	3.636317
Mg	-2.637116	-2.495084	-1.530195
O	-2.989447	-2.361712	0.487669
O	5.011020	-1.790950	4.917831
H	5.573239	-2.555715	4.699076
H	5.567781	-1.016648	4.766619
O	2.728070	0.083122	5.096927
H	2.236895	0.575615	4.421464
H	2.030526	-0.392506	5.584849
O	-1.235742	-0.763437	4.202824
H	-1.967800	-1.015640	4.814258
H	-1.596648	-0.470415	3.325205
O	-1.916428	-4.526020	-4.966781
O	-3.506923	-0.660524	-2.097044
H	-3.836366	-0.294829	-1.222024
H	-2.798874	-0.102443	-2.492604
O	-2.340958	-4.588499	-1.305541
H	-3.267758	-4.842400	-1.434185
H	-1.769869	-5.254878	-1.788743
H	3.598033	1.892966	-1.029710
H	-0.385284	1.706226	-0.165363
O	0.914495	-0.612695	0.758390
H	11.366477	-5.560755	-0.594579
H	9.642983	-5.916587	-0.380213
H	1.920058	-9.044550	-3.612128
H	-2.547100	-7.331758	-6.574478
H	-7.632537	-5.926289	0.180670
H	-11.426271	-1.478070	-5.880219
H	-11.723528	-2.241120	-1.049189
H	-2.983881	4.217112	9.521971
H	3.985685	-3.732442	8.733509
H	6.817151	-6.364150	4.470018
H	-3.400964	-2.479049	8.913669
H	5.828867	-3.738376	-4.279438
O	-1.186722	-3.538063	4.881696
H	-0.542637	-3.867311	5.566580
H	-1.868663	-3.050539	5.386584
H	8.289184	-0.308421	-5.811389
H	9.462840	5.873981	-7.385044
H	7.562321	2.093911	-6.084038
H	4.382362	-6.300135	0.511771

H	8.771391	3.359592	-2.031460
H	10.721799	5.423501	-6.205421
H	9.301399	2.189868	1.263295
H	2.440174	-2.877423	8.856230
H	4.027106	10.801680	0.822209
H	2.770205	8.000732	-4.978908
H	3.829095	0.643242	-3.317811
H	1.180747	3.096949	-5.891602
H	1.276249	4.697842	-5.118341
H	-4.759690	7.587544	-0.655091
H	9.685626	-1.325815	-6.281471
H	0.036046	-7.422055	5.373548
O	-1.549149	-2.581119	-3.278952
H	-1.776473	-3.260251	-3.983590
H	-1.432916	-1.676859	-3.664957

INT2

C	-10.910394	-1.048421	0.191569
C	-10.234800	0.314136	0.197488
O	-10.870654	1.359241	0.321347
N	-8.879776	0.255102	0.097451
C	-8.066821	1.449614	0.006537
C	-7.785293	1.897768	-1.438684
O	-7.402547	3.046133	-1.656723
C	-6.771124	1.268188	0.824035
C	-7.071748	1.202339	2.338914
C	-6.164729	0.256504	3.141894
C	-4.695674	0.672425	3.163970
N	-3.834373	-0.429901	3.704476
N	-8.011982	0.980447	-2.438751
C	-7.664104	1.326381	-3.808225
C	-8.711549	0.943894	-4.873087
O	-8.458073	1.126688	-6.063199
C	-6.289245	0.751011	-4.230968
O	-6.331643	-0.679256	-4.223196
C	-5.150914	1.266415	-3.361509
N	-9.889503	0.407601	-4.446516
C	-10.821127	-0.256532	-5.368876
C	-10.510672	-1.765404	-5.441942
C	-9.158671	-2.097340	-6.100217
C	-8.708774	-3.554686	-5.861684
C	-7.887145	-3.748547	-4.581642
N	-6.454551	-3.377374	-4.784481
C	-4.079065	7.571402	0.815196
C	-2.745439	7.965419	1.420304
O	-1.722750	8.173694	0.750489
C	-4.128509	6.067581	0.412422
O	-3.234916	5.871760	-0.672555
C	-5.547476	5.658502	0.020465
N	-2.706976	8.072468	2.774638
C	-1.497878	8.480841	3.483770
C	-0.634932	7.318065	4.009788
O	0.588340	7.414904	4.041367
C	-1.859791	9.373477	4.678250
N	-1.334626	6.268023	4.517649
C	-0.787017	5.396167	5.532494
C	-1.629385	5.540199	6.813961
O	-2.558787	6.335637	6.875562
N	-1.272106	4.703184	7.837211
C	-2.292695	4.232135	8.785386
C	-2.645060	2.755922	8.478080
C	-2.792383	2.565260	6.963893
O	-3.543697	3.269327	6.300679
N	-1.918796	1.676089	6.394715
C	3.909684	-5.703695	0.838383
C	2.487736	-5.472679	1.342920
C	2.349261	-4.451021	2.463273
O	1.180441	-4.179085	2.848615
O	3.414047	-3.922859	2.941208
C	9.766786	-5.799608	-1.635189
C	9.748651	-4.344127	-1.153461
C	10.857497	-3.530574	-1.834347
C	8.384244	-3.705251	-1.437517
C	6.225380	-5.924385	4.312766
C	7.018384	-4.719439	3.848823
C	6.889884	-4.239628	2.533735
C	7.882322	-4.029327	4.715860
C	7.596878	-3.118721	2.101334
C	8.586711	-2.895605	4.285746
C	8.447772	-2.433412	2.976479
C	-0.935706	-7.317910	4.361398
C	-0.421610	-7.046123	2.939806
C	-1.506147	-7.021868	1.844131
C	-2.299527	-5.719998	1.690557
N	-1.559438	-4.634527	0.974539
C	-3.527256	-1.384496	8.879779
C	-3.148499	-1.155260	7.383828
O	-1.929617	-1.427315	7.057383
O	-4.017304	-0.690016	6.619078
C	3.263284	-3.931286	7.953342
C	3.111453	-4.728497	6.651618
C	1.827731	-4.294887	5.973332
O	0.813784	-4.989993	6.110679
O	1.856800	-3.144150	5.346985
C	8.604292	-1.265705	-6.105041
C	8.040930	-2.460435	-5.337005
O	8.712185	-3.468000	-5.112656
N	6.736352	-2.322250	-4.983985
C	6.000980	-3.318339	-4.221136
C	5.310708	-2.639113	-3.012590
C	4.382173	-3.561330	-2.185743
C	2.854509	-3.405525	-2.409501
C	2.170402	-2.465470	-1.404505
N	0.681724	-2.483690	-1.571809
C	0.166303	-8.152203	-3.639273
C	0.706413	-6.979472	-2.804387
C	-0.339387	-6.189602	-1.995511
O	-1.534546	-6.557209	-2.046995
O	0.102787	-5.208629	-1.291380
C	-2.801470	-6.077724	-6.082643
C	-3.869106	-5.029495	-5.755938
O	-5.019258	-5.145141	-6.246082
C	1.774392	4.156971	-5.641988
C	2.907351	3.660980	-4.763999
O	3.744499	4.461545	-4.286252
N	2.962096	2.343008	-4.538330
C	3.964164	1.766862	-3.652709
C	5.343775	1.557032	-4.288749
O	5.835626	0.428838	-4.399898
N	5.999196	2.673468	-4.668462
C	7.352603	2.586948	-5.170518
C	7.838460	3.967643	-5.602157
O	7.281659	4.999614	-5.244073
N	8.949760	3.944479	-6.395966
C	9.601279	5.147885	-6.889575
C	2.381466	7.640926	-4.238852
C	0.973026	7.789719	-4.824190
C	-0.118309	7.150317	-3.954814
C	-0.371919	7.921204	-2.659642
N	-1.128674	7.091544	-1.692120
C	4.647021	9.698140	1.327213
C	4.040779	8.651995	0.374940
C	3.510235	7.383687	1.074109
N	2.975222	6.402078	0.126391
C	3.698639	5.548165	-0.629445
N	3.083096	4.794927	-1.537512
N	5.029797	5.431916	-0.481494
P	-0.780733	-0.932339	1.574415
O	-1.318228	0.162413	2.489292
O	-1.037763	-0.985580	0.089388
O	-0.689709	-2.297785	2.269286
P	-4.402846	-2.204187	0.828269
O	-4.433284	-2.594512	2.292844
O	-4.212565	-0.731490	0.492750
P	-6.521541	-2.658960	-1.237795
O	-7.755432	-1.802658	-1.425015
O	-5.401293	-2.438063	-2.276945
O	-5.987982	-2.575689	0.265488
O	-6.905558	-4.229359	-1.433862
C	-7.811349	-4.810212	-0.494279
C	1.934238	-0.747436	2.182987
C	8.327055	0.366488	-0.814678
C	8.102824	1.871415	-0.680448
O	-0.529060	-0.467826	-2.977609
C	0.034275	0.571600	-3.461495
O	1.013260	0.548524	-4.243376
C	-0.528587	1.922749	-2.980630
C	0.301817	2.497585	-1.807064
C	-0.322775	3.834880	-1.352207
O	-1.435496	3.812811	-0.803124
O	0.357574	4.913028	-1.598195
N	0.400575	1.563233	-0.672323
O	1.779393	-1.395067	3.272932
C	3.264215	-0.408963	1.797245
N	4.328568	-0.913808	2.558454
C	5.422444	-0.570028	1.923794
N	5.138663	0.138913	0.774912
C	3.766170	0.273402	0.678781
N	3.163851	0.922372	-0.337298
C	6.100374	0.521161	-0.268677
O	7.224071	-0.296142	-0.107445
O	8.144966	2.455529	-2.053550
C	6.619607	1.979325	-0.229603
O	5.863669	2.818224	-1.128328
C	9.632221	-0.071859	-0.128951
O	9.909945	0.750922	0.979387
H	-10.311068	-1.799731	-0.330631
H	-11.062160	-1.382878	1.223471
H	-6.094500	2.096526	0.593239
H	-6.256908	0.357735	0.500098
H	-8.099951	0.847655	2.470492
H	-7.040999	2.213562	2.767475
H	-6.229368	-0.745152	2.705289
H	-6.534962	0.179422	4.172248
H	-4.533678	1.565044	3.778125
H	-4.336816	0.845110	2.149474
H	-2.862157	-0.255846	3.390729
H	-4.106297	-1.351877	3.224663
H	-3.886716	-0.529888	4.739911
H	-8.659038	2.267340	0.426758
H	-8.440321	-0.629139	-0.163744
H	-6.145540	1.047283	-5.273610
H	-5.127414	2.359397	-3.358596
H	-5.262389	0.930767	-2.326787
H	-4.195307	0.875245	-3.716465
H	-5.966617	-1.031334	-3.385920
H	-7.582676	2.417326	-3.832262
H	-7.999045	-0.010607	-2.183411
H	-11.316586	-2.277851	-5.985872
H	-10.530107	-2.163855	-4.417672
H	-8.389934	-1.416997	-5.726153
H	-9.233280	-1.898651	-7.175024
H	-8.116424	-3.919813	-6.709238
H	-9.587458	-4.209217	-5.806206
H	-7.889042	-4.790015	-4.254431
H	-8.266486	-3.136525	-3.758773
H	-5.929536	-4.032506	-5.452247
H	-5.950813	-3.398936	-3.883172
H	-6.367968	-2.390240	-5.069885
H	-10.695138	0.221039	-6.342940
H	-9.985290	0.251567	-3.451689
H	-3.798898	5.470704	1.275279
H	-6.240752	5.764086	0.861810
H	-5.905642	6.282960	-0.804657
H	-5.580739	4.620386	-0.318807

H	-2.707394	5.044602	-0.580409
H	-4.897465	7.781671	1.510873
H	-2.437887	8.813010	5.421048
H	-0.944720	9.720108	5.164009
H	-2.434947	10.243456	4.348683
H	-0.876561	9.018525	2.767422
H	-3.570841	7.987850	3.291257
H	0.257254	5.676868	5.699637
H	-0.814259	4.346750	5.215650
H	-2.338166	6.373359	4.590411
H	-3.592596	2.493025	8.958777
H	-1.874957	2.087297	8.878894
H	-2.116088	1.480000	5.420754
H	-1.620380	0.853471	6.911769
H	-3.161523	4.873546	8.637276
H	-0.570743	4.012641	7.595502
H	4.383904	-4.760466	0.560704
H	4.530570	-6.176690	1.604157
H	2.051613	-6.407431	1.713664
H	1.820999	-5.166747	0.527076
H	9.921575	-4.334836	-0.067064
H	10.681257	-3.489111	-2.915726
H	10.882090	-2.502292	-1.458547
H	11.844645	-3.975456	-1.663330
H	8.323988	-2.682004	-1.054894
H	8.223710	-3.664752	-2.518077
H	7.565939	-4.281822	-0.989410
H	9.583731	-5.842518	-2.715322
H	6.335858	-6.086360	5.388435
H	5.162667	-5.788052	4.086294
H	6.212484	-4.738656	1.852419
H	8.002110	-4.382796	5.737272
H	7.466841	-2.755303	1.088037
H	9.246040	-2.380016	4.978166
H	8.978417	-1.547113	2.636255
H	-1.674962	-6.566771	4.661636
H	-1.410794	-8.303802	4.432017
H	0.308577	-7.824080	2.678757
H	0.120370	-6.097638	2.936630
H	-2.233059	-7.821221	2.039507
H	-1.065842	-7.256106	0.867213
H	-2.620197	-5.301668	2.648011
H	-3.196336	-5.899082	1.093468
H	-2.284875	-3.944351	0.619786
H	-0.932076	-4.088656	1.580137
H	-1.017569	-4.969865	0.146972
H	-2.960637	-0.685781	9.505702
H	-4.596044	-1.226687	9.027153
H	3.038968	-5.797037	6.864715
H	3.971418	-4.552538	6.001989
H	2.383168	-4.061019	8.590017
H	9.658800	-1.150108	-5.847249
H	4.748189	-1.772327	-3.379679
H	6.097115	-2.227440	-2.371163
H	4.592653	-3.406366	-1.121408
H	4.652874	-4.606129	-2.379026
H	2.366726	-4.379973	-2.304957
H	2.654276	-3.054513	-3.429943
H	2.501564	-1.428415	-1.502979
H	2.383541	-2.791179	-0.382299
H	0.164601	-1.990797	-0.802081
H	0.380283	-1.941030	-2.406514
H	0.337274	-3.469887	-1.618961
H	6.703204	-4.091028	-3.905172
H	6.325262	-1.395909	-5.032652
H	-0.380000	-8.856139	-3.006482
H	-0.528547	-7.796691	-4.404750
H	1.229141	-6.257231	-3.445125
H	1.462890	-7.327049	-2.088636
H	-1.901476	-5.585246	-6.462780
H	-2.521255	-6.591385	-5.155534
H	2.191094	4.803262	-6.417899
H	4.107707	2.395691	-2.769484
H	2.120783	1.740740	-4.665295
H	8.010173	2.203949	-4.376725
H	5.572641	3.592433	-4.553860
H	9.021271	5.998019	-6.528810
H	9.336453	3.046958	-6.647161
H	2.460085	8.083498	-3.238645
H	2.687572	6.593569	-4.167309
H	0.946832	7.321067	-5.814387
H	0.738195	8.850758	-4.986668
H	0.177791	6.131381	-3.683629
H	-1.058733	7.074053	-4.514580
H	-0.917003	8.852435	-2.841182
H	0.571788	8.181793	-2.172787
H	-1.319893	7.561116	-0.779710
H	-2.060915	6.798629	-2.002194
H	-0.577535	6.181779	-1.519934
H	3.911823	10.037283	2.064397
H	5.503552	9.289248	1.874316
H	4.787678	8.352649	-0.370574
H	3.212978	9.099933	-0.188473
H	2.705846	7.623769	1.776446
H	4.297654	6.909629	1.673252
H	1.988923	6.438914	-0.094676
H	2.049736	4.842585	-1.636410
H	3.606285	4.507415	-2.366661
H	5.485359	5.955439	0.247969
H	5.444103	4.515654	-0.734194
H	-1.561256	1.797723	-2.646120
H	-0.504053	2.636682	-3.811523
H	1.310327	2.705912	-2.185121
H	-0.045791	0.666854	-0.877546
H	-8.778490	-4.294243	-0.515176
H	-7.395730	-4.761912	0.516761
H	8.308702	0.031676	-1.864766
H	6.434569	-0.808253	2.199562
H	2.127934	1.106202	-0.319728
H	5.599530	0.348911	-1.235434
H	6.481488	2.381140	0.784867
H	6.523415	2.909312	-1.869602
H	10.434773	-0.024517	-0.888368
H	9.562953	-1.115007	0.203286
Mg	3.414835	-2.302197	4.109680
Mg	0.243362	-2.669602	3.982451
Mg	-3.419207	-2.796964	-1.994835
O	-3.504442	-3.135497	-0.048942
O	5.167674	-2.553464	5.151736
H	5.933639	-3.038543	4.787408
H	5.501026	-1.739397	5.548848
O	3.013769	-0.793567	5.662450
H	2.397751	-0.191220	5.215515
H	2.408524	-1.427608	6.096357
O	-0.381899	-0.875627	4.936156
H	-1.083000	-1.000435	5.639194
H	-0.750675	-0.359105	4.180979
O	-3.534369	-4.096093	-4.945407
O	-3.086795	-0.775320	-1.851242
H	-3.411554	-0.570804	-0.923011
H	-2.145697	-0.526243	-1.897824
O	-3.482743	-4.804420	-2.373923
H	-3.498583	-4.838882	-3.354075
H	-2.768773	-5.437741	-2.093782
H	3.726105	1.608862	-0.823600
H	-0.137608	1.916424	0.116450
O	0.967181	-0.411526	1.405831
H	10.734026	-6.275837	-1.437697
H	8.990603	-6.393421	-1.138954
H	0.985827	-8.683463	-4.134570
H	-3.166831	-6.805314	-6.808608
H	-7.946964	-5.853326	-0.786791
H	-11.844259	-0.086894	-5.019557
H	-11.891584	-0.955047	-0.278228
H	-1.942679	4.339766	9.816266
H	4.142034	-4.260966	8.516439
H	6.556552	-6.833504	3.798913
H	-3.243676	-2.395858	9.184080
H	5.244504	-3.800511	-4.857952
O	-1.175934	-3.617329	5.141280
H	-0.600647	-4.261920	5.632183
H	-1.590720	-3.028994	5.805041
H	8.058945	-0.346295	-5.878291
H	9.620282	5.165090	-7.984538
H	7.403224	1.884012	-6.010509
H	3.902348	-6.353810	-0.042008
H	8.748185	3.199609	-1.974320
H	10.627668	5.228467	-6.514847
H	9.524117	1.618954	0.653876
H	3.382865	-2.863211	7.748370
H	4.992533	10.570445	0.766217
H	3.117699	8.150078	-4.868869
H	3.594124	0.791512	-3.341608
H	1.214135	3.339013	-6.098725
H	1.093179	4.758674	-5.035526
H	-4.236520	8.168320	-0.087928
H	8.535706	-1.465170	-7.179652
H	-0.119273	-7.266564	5.086461
O	-1.897044	-2.777806	-3.392489
H	-2.239310	-3.250628	-4.191329
H	-1.506718	-1.910388	-3.619245

TS4

C	-10.980318	-1.070744	0.038404
C	-10.119714	0.141308	-0.264491
O	-10.605696	1.269636	-0.339353
N	-8.796057	-0.125406	-0.414229
C	-7.859909	0.919338	-0.776481
C	-7.471312	0.890218	-2.269284
O	-7.001449	1.903219	-2.790963
C	-6.672309	0.946310	0.213741
C	-7.165409	1.347586	1.633066
C	-6.608320	0.516994	2.809390
C	-5.201638	0.933921	3.246222
N	-4.525849	-0.120700	4.083596
N	-7.761202	-0.254613	-2.973107
C	-7.461395	-0.328784	-4.395501
C	-8.520112	-1.041511	-5.280742
O	-8.273472	-1.204597	-6.474689
C	-6.066053	-0.949086	-4.686088
O	-5.978367	-2.276597	-4.171493
C	-4.920494	-0.091637	-4.157705
N	-9.696560	-1.452368	-4.719251
C	-10.595237	-2.412607	-5.402603
C	-10.241629	-3.846254	-4.952978
C	-8.830010	-4.309603	-5.363796
C	-8.363372	-5.585811	-4.626040
C	-7.570885	-5.316887	-3.338664
N	-6.136860	-5.025915	-3.625985
C	-4.120530	7.238445	-2.254656
C	-2.978895	7.938619	-1.531581
O	-1.812800	7.938819	-1.948120
C	-4.294837	5.749464	-1.883798
O	-3.109120	5.050546	-2.302978
C	-5.538062	5.152351	-2.537837
N	-3.303245	8.602735	-0.394958
C	-2.336894	9.465324	0.287206
C	-1.350978	8.731639	1.209085
O	-0.266606	9.240527	1.457855
C	-3.077989	10.508830	1.130894
N	-1.797568	7.567775	1.764295
C	-1.241314	7.129717	3.023519
C	-2.045770	7.729783	4.194223
O	-2.876501	8.609209	4.018112
N	-1.751888	7.192728	5.420601

C	-2.778726	7.140147	6.466306
C	-3.269756	5.691817	6.635648
C	-3.677471	5.095425	5.282365
O	-4.243923	5.775826	4.428893
N	-3.330729	3.800058	5.078526
C	3.747513	-4.970682	3.137704
C	2.336184	-4.413511	3.362470
C	2.180055	-2.950885	3.774427
O	1.009901	-2.564596	4.052050
O	3.200423	-2.182265	3.793869
C	9.772225	-5.910644	0.952825
C	9.693043	-4.384463	1.084488
C	10.777479	-3.713152	0.230568
C	8.304810	-3.890418	0.663154
C	5.877409	-3.849972	6.556774
C	6.640854	-2.830981	5.733506
C	6.581250	-2.851292	4.327835
C	7.395846	-1.817741	6.340996
C	7.240872	-1.886896	3.560160
C	8.058991	-0.851887	5.576564
C	7.982688	-0.875849	4.180593
C	-1.287683	-5.209340	6.754995
C	-0.496825	-5.175367	5.441197
C	-1.171546	-5.892779	4.254132
C	-2.222875	-5.101193	3.463185
N	-1.628840	-4.179377	2.452885
C	-4.062363	1.951204	8.589212
C	-3.860944	1.280153	7.196211
O	-2.730721	1.462811	6.603767
O	-4.825249	0.636047	6.726589
C	2.746191	-0.676295	9.031438
C	2.831697	-0.289737	7.552063
C	1.579317	-0.626290	6.747776
O	0.675533	-1.281050	7.276343
O	1.534470	-0.174078	5.511825
C	8.833183	-3.392694	-4.710347
C	8.237095	-4.124428	-3.510490
O	8.951058	-4.644892	-2.652044
N	6.878051	-4.144717	-3.477088
C	6.118769	-4.623642	-2.332153
C	5.436493	-3.455774	-1.588099
C	4.505564	-3.899144	-0.442984
C	3.048646	-4.244771	-0.830845
C	2.124411	-3.020547	-0.821012
N	0.678362	-3.398455	-0.888696
C	0.222371	-8.959082	-0.286300
C	0.785553	-7.714467	0.398238
C	-0.170005	-6.517616	0.455874
O	-1.360866	-6.646240	0.117676
O	0.355141	-5.417080	0.891227
C	-2.597246	-7.983074	-3.476759
C	-3.599813	-6.835106	-3.672063
O	-4.733632	-7.131282	-4.169052
C	2.032948	1.728706	-6.658494
C	3.073509	1.189041	-5.695944
O	3.768727	1.935634	-4.993308
N	3.199868	-0.154663	-5.658568
C	4.059996	-0.841677	-4.705901
C	5.512946	-0.987448	-5.168496
O	5.984731	-2.110293	-5.381077
N	6.225603	0.149939	-5.306697
C	7.644787	0.101702	-5.611423
C	8.100563	1.364539	-6.338025
O	7.511455	2.433322	-6.216459
N	9.239699	1.197763	-7.075641
C	9.921881	2.274657	-7.782503
C	2.594558	5.491553	-6.629735
C	1.086825	5.201241	-6.575219
C	0.517835	4.886366	-5.175302
C	0.269032	6.131674	-4.321938
N	-0.477778	5.816548	-3.068571
C	4.584121	9.506110	-2.127404
C	4.147042	8.093444	-2.542251
C	4.189728	7.067346	-1.395041
N	3.652186	5.772148	-1.787423
C	4.330230	4.734526	-2.298037
N	3.645000	3.643079	-2.661315
N	5.664831	4.739425	-2.473222
P	-0.922091	-0.805429	1.120233
O	-1.468735	0.622788	1.211788
O	-1.273810	-1.701530	-0.065730
O	-0.934766	-1.509406	2.486638
P	-4.493396	-2.238855	1.594152
O	-4.840983	-2.604927	3.028295
O	-4.071547	-0.786189	1.398596
P	-6.203561	-3.045223	-0.714217
O	-7.542113	-2.545957	-1.231330
O	-5.006175	-2.922371	-1.665222
O	-5.960636	-2.367783	0.719811
O	-6.289116	-4.643564	-0.407667
C	-7.097240	-5.069739	0.692906
C	1.565610	0.214810	1.468279
C	8.518597	-0.089100	-0.561719
C	8.356965	1.271008	-1.221319
O	-1.430726	0.366311	-2.487712
C	-0.284142	0.744180	-2.093635
O	0.808646	0.202818	-2.382759
C	-0.312947	2.058592	-1.283424
C	0.932840	2.402080	-0.456037
C	1.228877	3.918121	-0.435088
O	1.499752	4.488337	0.620459
O	1.248751	4.479092	-1.618219
N	0.875419	1.903023	0.935673
O	1.391920	0.235791	2.770054
C	3.004902	0.247744	1.053683
N	3.927413	0.302265	2.099911
C	5.131362	0.314111	1.569652
N	5.053971	0.261529	0.210025
C	3.711094	0.230963	-0.146995
N	3.372551	0.164711	-1.483130
C	6.203918	0.185764	-0.697082
O	7.207622	-0.514837	-0.017330
O	8.578943	1.089409	-2.701539
C	6.839898	1.548332	-1.091870
O	6.297019	2.036140	-2.330844
C	9.501828	-0.053559	0.617454
O	9.474357	1.192697	1.284537
H	-10.446920	-2.015703	-0.097786
H	-11.334243	-1.009821	1.072275
H	-5.916615	1.641778	-0.162946
H	-6.199612	-0.035478	0.243373
H	-8.253920	1.237986	1.656089
H	-6.974751	2.415537	1.807848
H	-6.597559	-0.536622	2.521417
H	-7.285938	0.604172	3.667647
H	-5.240370	1.845037	3.853442
H	-4.572550	1.076918	2.371394
H	-3.524031	-0.107404	3.889198
H	-4.799292	-1.108346	3.785180
H	-4.660268	0.057776	5.116750
H	-8.415001	1.857704	-0.685350
H	-8.449844	-1.086419	-0.415673
H	-6.006278	-1.039873	-5.773898
H	-5.016684	0.944230	-4.492205
H	-4.884608	-0.093792	-3.064797
H	-3.965600	-0.487922	-4.519072
H	-5.653779	-2.270876	-3.242799
H	-7.429640	0.705208	-4.753099
H	-7.774476	-1.134012	-2.443053
H	-10.988090	-4.548001	-5.350836
H	-10.334808	-3.888900	-3.858361
H	-8.109068	-3.510461	-5.175790
H	-8.816497	-4.473711	-6.447004
H	-7.744041	-6.205173	-5.285513
H	-9.234103	-6.201868	-4.368943
H	-7.575502	-6.192766	-2.685988
H	-7.981274	-4.473591	-2.776031
H	-5.571057	-5.913030	-3.989598
H	-5.635063	-4.753691	-2.771622
H	-6.037633	-4.208967	-4.242233
H	-10.438706	-2.275586	-6.474059
H	-9.782128	-1.343822	-3.716775
H	-4.379594	5.673443	-0.788982
H	-6.441514	5.668097	-2.197439
H	-5.474112	5.246732	-3.626655
H	-5.665540	4.090509	-2.299677
H	-3.239628	4.112948	-2.099975
H	-5.067626	7.747424	-2.051589
H	-3.607156	10.028719	1.959572
H	-2.348916	11.195393	1.564390
H	-3.778537	11.080895	0.514102
H	-1.723546	9.947139	-0.477438
H	-4.275408	8.670225	-0.128226
H	-0.201428	7.464220	3.062474
H	-1.257870	6.036976	3.083577
H	-2.779599	7.362238	1.640262
H	-4.143090	5.674617	7.299053
H	-2.500885	5.070419	7.110471
H	-3.688970	3.385038	4.229315
H	-2.998448	3.149231	5.797950
H	-3.590400	7.784122	6.126971
H	-1.117166	6.403678	5.406126
H	4.310928	-4.345582	2.440555
H	4.311841	-5.028366	4.073422
H	1.813989	-5.003847	4.120325
H	1.735799	-4.546883	2.454545
H	9.858620	-4.115227	2.138503
H	10.603102	-3.925187	-0.829927
H	10.770756	-2.626680	0.364665
H	11.776861	-4.077050	0.497697
H	8.212216	-2.804955	0.743327
H	8.137936	-4.154128	-0.383529
H	7.512583	-4.344736	1.270917
H	9.606943	-6.203292	-0.090445
H	6.031711	-3.692594	7.627260
H	4.803055	-3.782493	6.352290
H	5.994959	-3.613133	3.829468
H	7.454037	-1.777553	7.425555
H	7.161036	-1.912487	2.479077
H	8.636188	-0.078008	6.074752
H	8.490565	-0.125513	3.579215
H	-2.269463	-4.734745	6.644619
H	-1.451878	-6.239480	7.093084
H	0.471330	-5.660448	5.621567
H	-0.262451	-4.142318	5.168710
H	-1.654394	-6.808587	4.619124
H	-0.405124	-6.220708	3.541417
H	-2.883866	-4.503737	4.096451
H	-2.848945	-5.790052	2.891233
H	-2.391003	-3.815318	1.812438
H	-1.187212	-3.330399	2.835651
H	-0.917479	-4.658613	1.856388
H	-4.232297	3.024505	8.442797
H	-4.928949	1.520385	9.091242
H	3.679404	-0.782537	7.064584
H	3.027725	0.783339	7.433478
H	2.566580	-1.748626	9.138854
H	9.264976	-2.452095	-4.351283
H	4.871910	-2.848536	-2.309249
H	6.221561	-2.805348	-1.188140
H	4.481889	-3.110819	0.319417
H	4.954128	-4.769750	0.050293
H	2.642016	-4.960875	-0.108813
H	3.020249	-4.738057	-1.812333
H	2.328505	-2.311760	-1.625731
H	2.238992	-2.476669	0.117798

H	0.036424	-2.603082	-0.635139
H	0.358849	-3.723329	-1.802925
H	0.466731	-4.175910	-0.204173
H	6.811888	-5.150576	-1.675758
H	6.387613	-3.587701	-4.170229
H	-0.705769	-9.275931	0.195991
H	-0.014989	-8.755055	-1.334392
H	1.704539	-7.372528	-0.093938
H	1.079766	-7.934349	1.433424
H	-1.740905	-7.831998	-4.143172
H	-2.220371	-7.955359	-2.448797
H	2.480287	2.533508	-7.243799
H	4.022092	-0.328648	-3.744183
H	2.589454	-0.716291	-6.231408
H	8.219187	0.033901	-4.674700
H	5.789569	1.051760	-5.133863
H	9.343445	3.184787	-7.618388
H	9.639217	0.272152	-7.118289
H	2.858455	6.385053	-6.052516
H	3.167562	4.654145	-6.216017
H	0.872445	4.344980	-7.222699
H	0.528186	6.042700	-7.006548
H	1.185168	4.201816	-4.638892
H	-0.440146	4.363137	-5.294823
H	-0.318087	6.868194	-4.878826
H	1.206943	6.606132	-4.020915
H	-0.826404	6.678414	-2.599439
H	-1.347443	5.294345	-3.232641
H	0.136653	5.257946	-2.393858
H	3.942045	9.899388	-1.332397
H	5.615602	9.511963	-1.757692
H	4.772351	7.726250	-3.365536
H	3.119237	8.125417	-2.925251
H	3.587027	7.417034	-0.551359
H	5.212405	6.958846	-1.012043
H	2.651334	5.573340	-1.622109
H	2.631216	3.671950	-2.544158
H	4.063718	2.955652	-3.276995
H	6.194388	5.480437	-2.042672
H	6.096808	3.797311	-2.487320
H	-1.202074	2.045561	-0.650978
H	-0.440602	2.849260	-2.030300
H	1.799627	1.953171	-0.948843
H	-0.107177	1.703062	1.192628
H	-8.134760	-4.739121	0.564584
H	-6.701579	-4.672537	1.632313
H	8.838611	-0.848357	-1.289991
H	6.074638	0.325571	2.089223
H	2.356490	0.171341	-1.698114
H	5.853242	-0.336475	-1.598947
H	6.594824	2.276243	-0.305534
H	7.028819	1.774041	-2.947966
H	10.507139	-0.269063	0.214428
H	9.269561	-0.836348	1.346891
Mg	3.021990	-0.163900	3.984855
Mg	-0.030779	-0.744163	4.089447
Mg	-2.969328	-2.586113	-1.031956
O	-3.568585	-3.230172	0.835365
O	4.800605	-0.292970	5.015516
H	5.202859	-1.165700	4.851703
H	5.526356	0.340924	4.978124
O	2.834495	1.932274	4.572375
H	2.367425	2.285319	3.801842
H	2.122128	1.648643	5.178719
O	-1.131774	1.092774	3.851348
H	-1.821637	1.318383	4.496390
H	-1.489544	1.043238	2.923052
O	-3.257350	-5.664992	-3.344458
O	-3.621723	-0.540612	-1.105518
H	-3.762817	-0.397691	-0.125429
H	-2.857617	-0.002312	-1.391823
O	-2.058055	-4.443998	-1.352984
H	-2.416577	-4.942848	-2.143291
H	-1.987934	-5.134355	-0.654851
H	3.863654	0.866984	-2.027894
H	1.263616	2.636098	1.533061
O	0.774147	-0.593767	0.728901
H	10.753295	-6.290787	1.260646
H	9.011011	-6.404892	1.567782
H	0.939424	-9.786179	-0.249336
H	-3.058774	-8.948502	-3.688746
H	-7.060919	-6.160813	0.706824
H	-11.631312	-2.165069	-5.153773
H	-11.860492	-1.047579	-0.609317
H	-2.389928	7.527648	7.413543
H	3.677462	-0.422289	9.547610
H	6.190042	-4.870752	6.312422
H	-3.161991	1.836442	9.198087
H	5.365334	-5.346911	-2.672828
O	-1.367002	-1.006884	5.669479
H	-0.744644	-1.249561	6.405537
H	-1.872199	-0.231392	5.999783
H	8.096552	-3.169296	-5.481216
H	9.971671	2.070140	-8.857102
H	7.837843	-0.798399	-6.199012
H	3.684106	-5.979498	2.718854
H	9.259339	1.737757	-2.900867
H	10.937458	2.419619	-7.398725
H	9.423530	1.818961	0.518484
H	1.920902	-0.157627	9.526185
H	4.529907	10.193451	-2.976171
H	2.922366	5.656596	-7.660322
H	3.677397	-1.851651	-4.564117
H	1.632612	0.967193	-7.331503
H	1.207180	2.145050	-6.075344
H	-3.922086	7.308874	-3.328131
H	9.648666	-3.997302	-5.114396
H	-0.751902	-4.670400	7.542531
O	-2.061608	-2.122270	-2.889603
H	-2.683914	-2.164736	-3.625925
H	-1.723894	-1.171216	-2.843650

INT3

C	-10.970113	-1.030384	0.025972
C	-10.107919	0.178844	-0.283428
O	-10.591793	1.307735	-0.363943
N	-8.784720	-0.091346	-0.431290
C	-7.846258	0.949230	-0.799442
C	-7.454889	0.910094	-2.291372
O	-6.982960	1.919608	-2.818883
C	-6.659965	0.980687	0.191882
C	-7.153720	1.394002	1.607368
C	-6.607469	0.564349	2.788980
C	-5.199418	0.973048	3.229547
N	-4.533865	-0.083463	4.071642
N	-7.744664	-0.238449	-2.988661
C	-7.445503	-0.321486	-4.410696
C	-8.504605	-1.039432	-5.291530
O	-8.257243	-1.211426	-6.484150
C	-6.049377	-0.941966	-4.698159
O	-5.957090	-2.266562	-4.177925
C	-4.905054	-0.078719	-4.175652
N	-9.682545	-1.443633	-4.728148
C	-10.585479	-2.403273	-5.407324
C	-10.235431	-3.836400	-4.953454
C	-8.825026	-4.305010	-5.362525
C	-8.360542	-5.577893	-4.617738
C	-7.570891	-5.302918	-3.329777
N	-6.135527	-5.015197	-3.614293
C	-4.079774	7.242349	-2.306966
C	-2.909137	7.912793	-1.604320
O	-1.750369	7.879155	-2.041205
C	-4.266658	5.750687	-1.949968
O	-3.109040	5.032166	-2.416775
C	-5.541024	5.184147	-2.570483
N	-3.194741	8.583727	-0.462290
C	-2.185024	9.393809	0.220924
C	-1.240134	8.604892	1.141137
O	-0.134496	9.058707	1.400920
C	-2.869588	10.474511	1.065382
N	-1.744476	7.455105	1.679452
C	-1.231142	6.991224	2.949073
C	-2.020201	7.642245	4.104184
O	-2.822550	8.543754	3.902398
N	-1.749054	7.125075	5.340867
C	-2.745112	7.187061	6.415469
C	-3.309427	5.778722	6.672473
C	-3.794722	5.166965	5.353256
O	-4.461872	5.823515	4.557985
N	-3.380358	3.898209	5.099902
C	3.741680	-4.964301	3.158287
C	2.331255	-4.392380	3.346706
C	2.175104	-2.924426	3.740108
O	1.000713	-2.533243	3.990649
O	3.196844	-2.157434	3.771990
C	9.735718	-5.934950	0.947460
C	9.690812	-4.409914	1.106645
C	10.800907	-3.749269	0.277910
C	8.319874	-3.876205	0.676227
C	5.872783	-3.832290	6.572868
C	6.622725	-2.810901	5.739880
C	6.555603	-2.841286	4.335096
C	7.373418	-1.788237	6.335858
C	7.205002	-1.878907	3.556602
C	8.027791	-0.824749	5.560728
C	7.945656	-0.859603	4.165341
C	-1.297126	-5.165756	6.772802
C	-0.502580	-5.100496	5.462323
C	-1.150884	-5.837200	4.271981
C	-2.210933	-5.069605	3.469071
N	-1.625776	-4.146891	2.454641
C	-4.048386	2.014271	8.565674
C	-3.850615	1.343119	7.171074
O	-2.723821	1.525940	6.572089
O	-4.817710	0.699081	6.706984
C	2.750678	-0.634368	9.027715
C	2.876427	-0.413558	7.517274
C	1.587394	-0.678194	6.742160
O	0.670071	-1.299437	7.292121
O	1.531566	-0.214311	5.516470
C	8.838910	-3.446858	-4.694212
C	8.239719	-4.171144	-3.491244
O	8.952119	-4.687468	-2.629147
N	6.880403	-4.190753	-3.460781
C	6.118392	-4.655386	-2.311502
C	5.440661	-3.473913	-1.582873
C	4.502093	-3.893490	-0.434535
C	3.040622	-4.222271	-0.820778
C	2.125574	-2.990756	-0.805477
N	0.677686	-3.362813	-0.880664
C	0.205403	-8.959136	-0.246702
C	0.775701	-7.703430	0.412982
C	-0.178579	-6.504399	0.465697
O	-1.372819	-6.636944	0.139970
O	0.350589	-5.399628	0.884446
C	-2.608299	-7.990838	-3.444686
C	-3.602611	-6.836334	-3.646945
O	-4.739206	-7.127186	-4.141312
C	2.057988	1.687336	-6.675743
C	3.086418	1.179114	-5.683390
O	3.757990	1.950377	-4.983399
N	3.225548	-0.161443	-5.616438
C	4.085928	-0.825258	-4.646855
C	5.533170	-0.991771	-5.118530
O	5.993940	-2.119595	-5.327633

N	6.253232	0.139308	-5.272566
C	7.672129	0.079342	-5.576047
C	8.133009	1.326550	-6.325199
O	7.554417	2.402313	-6.213557
N	9.263498	1.138690	-7.070684
C	9.949647	2.199818	-7.796409
C	2.632628	5.448322	-6.667083
C	1.127867	5.152332	-6.569949
C	0.598239	4.815330	-5.158882
C	0.362309	6.048060	-4.283361
N	-0.404454	5.727955	-3.041177
C	4.632594	9.480523	-2.185157
C	4.159556	8.067039	-2.559893
C	4.228339	7.056524	-1.399555
N	3.661864	5.760700	-1.754654
C	4.311138	4.718205	-2.297581
N	3.604960	3.631717	-2.633578
N	5.635915	4.715250	-2.530962
P	-0.924976	-0.783215	1.131606
O	-1.473793	0.659123	1.213429
O	-1.293724	-1.679963	-0.049883
O	-0.988801	-1.464917	2.506990
P	-4.510846	-2.213169	1.595245
O	-4.870315	-2.567232	3.029303
O	-4.080041	-0.763610	1.391850
P	-6.217953	-3.027235	-0.713525
O	-7.548547	-2.519906	-1.243854
O	-5.015257	-2.924260	-1.660787
O	-5.974641	-2.336717	0.713595
O	-6.320215	-4.621183	-0.391578
C	-7.131978	-5.028803	0.713328
C	1.492267	0.392341	1.442850
C	8.459035	-0.088784	-0.541055
C	8.315145	1.279786	-1.194533
O	-1.444633	0.345254	-2.512317
C	-0.294811	0.678667	-2.091104
O	0.786289	0.107420	-2.361226
C	-0.300213	1.987685	-1.269868
C	0.931873	2.294674	-0.409213
C	1.212455	3.813060	-0.311450
O	1.421140	4.330438	0.785432
O	1.267777	4.422813	-1.462383
N	0.836481	1.776550	0.990696
O	1.350138	0.365826	2.791256
C	2.958281	0.353080	1.046768
N	3.868497	0.358649	2.101123
C	5.079367	0.330407	1.585868
N	5.013788	0.286600	0.228263
C	3.675968	0.303505	-0.144222
N	3.364756	0.237593	-1.492267
C	6.162893	0.201125	-0.670200
O	7.164326	-0.477058	0.035764
O	8.565313	1.103287	-2.670841
C	6.794353	1.557475	-1.090432
O	6.265784	2.014325	-2.348749
C	9.488164	-0.082881	0.601381
O	9.527616	1.161823	1.268384
H	-10.439421	-1.976783	-0.110970
H	-11.318457	-0.966686	1.061569
H	-5.902964	1.672295	-0.189630
H	-6.189266	-0.001891	0.229158
H	-8.243294	1.294979	1.628123
H	-6.953215	2.460894	1.777320
H	-6.603229	-0.490547	2.505554
H	-7.287788	0.660435	3.644165
H	-5.235660	1.885597	3.834911
H	-4.566158	1.111299	2.357073
H	-3.531768	-0.081470	3.877338
H	-4.816382	-1.069955	3.777504
H	-4.666469	0.102749	5.103367
H	-8.399744	1.889143	-0.715285
H	-8.441384	-1.053563	-0.428769
H	-5.990173	-1.038559	-5.785401
H	-5.005204	0.955374	-4.514509
H	-4.869051	-0.076063	-3.082769
H	-3.949472	-0.473090	-4.537092
H	-5.646366	-2.257011	-3.244410
H	-7.414645	0.710389	-4.774331
H	-7.762515	-1.114748	-2.453160
H	-10.983896	-4.537223	-5.348951
H	-10.328526	-3.875117	-3.858711
H	-8.102497	-3.505958	-5.179812
H	-8.812305	-4.475309	-6.444782
H	-7.740391	-6.200839	-5.273048
H	-9.232136	-6.192220	-4.359202
H	-7.578412	-6.174694	-2.671854
H	-7.982283	-4.455917	-2.773430
H	-5.568583	-5.906052	-3.970070
H	-5.637241	-4.735636	-2.759884
H	-6.034171	-4.202434	-4.235662
H	-10.429786	-2.269954	-6.479327
H	-9.767577	-1.330521	-3.726125
H	-4.317121	5.660634	-0.854218
H	-6.423471	5.708521	-2.190421
H	-5.512465	5.295918	-3.659137
H	-5.677266	4.120191	-2.345296
H	-3.271656	4.092334	-2.250145
H	-5.013664	7.761183	-2.071107
H	-3.426702	10.022101	1.891237
H	-2.104937	11.118812	1.502511
H	-3.535411	11.085470	0.447601
H	-1.548281	9.842770	-0.544387
H	-4.159144	8.678254	-0.176039
H	-0.174835	7.266246	3.003114
H	-1.307967	5.901109	3.013626
H	-2.736267	7.309038	1.548407
H	-4.159965	5.844270	7.360957
H	-2.558307	5.136452	7.147129
H	-3.784315	3.470196	4.277672
H	-3.004203	3.251245	5.799927
H	-3.532217	7.853417	6.062158
H	-1.145577	6.312082	5.354422
H	4.324602	-4.353695	2.464143
H	4.286469	-5.014111	4.105934
H	1.789354	-4.969837	4.100661
H	1.747888	-4.532466	2.429059
H	9.848807	-4.163157	2.167123
H	10.636850	-3.943572	-0.787753
H	10.814893	-2.664832	0.427099
H	11.788657	-4.138233	0.552751
H	8.249140	-2.791910	0.785739
H	8.163924	-4.109229	-0.379689
H	7.508592	-4.329047	1.259374
H	9.573292	-6.204131	-0.102506
H	6.041088	-3.675359	7.641359
H	4.795405	-3.767581	6.383302
H	5.972441	-3.611093	3.845639
H	7.437048	-1.739025	7.419771
H	7.117850	-1.912328	2.476363
H	8.603277	-0.044151	6.050376
H	8.449677	-0.113234	3.555576
H	-2.294910	-4.726761	6.658790
H	-1.425259	-6.201985	7.107822
H	0.480844	-5.552470	5.645419
H	-0.301363	-4.059517	5.193208
H	-1.618334	-6.760898	4.637146
H	-0.372733	-6.152901	3.566672
H	-2.882975	-4.476734	4.095006
H	-2.823652	-5.772406	2.899554
H	-2.393093	-3.786825	1.818808
H	-1.185898	-3.296242	2.836535
H	-0.916349	-4.626730	1.856681
H	-4.277759	3.075901	8.414726
H	-4.881340	1.545626	9.090226
H	3.647612	-1.066299	7.092144
H	3.215818	0.602954	7.288771
H	2.416980	-1.652558	9.238890
H	9.281844	-2.510580	-4.337715
H	4.883428	-2.871970	-2.314143
H	6.228980	-2.823531	-1.188943
H	4.489381	-3.098133	0.320712
H	4.937704	-4.765954	0.067002
H	2.628205	-4.938111	-0.101923
H	3.005776	-4.711240	-1.804365
H	2.333960	-2.280577	-1.608051
H	2.237920	-2.449868	0.135618
H	0.037548	-2.568157	-0.623373
H	0.362852	-3.679037	-1.799464
H	0.461476	-4.146672	-0.205353
H	6.808664	-5.176269	-1.646998
H	6.392355	-3.627923	-4.150925
H	-0.717435	-9.268692	0.250275
H	-0.043226	-8.771056	-1.295180
H	1.689016	-7.368704	-0.094601
H	1.082189	-7.908558	1.447600
H	-1.749459	-7.848560	-4.109795
H	-2.233595	-7.960796	-2.416003
H	2.502578	2.494081	-7.260103
H	4.059393	-0.282272	-3.701814
H	2.642267	-0.739777	-6.200925
H	8.245261	0.026647	-4.637479
H	5.827681	1.044807	-5.095829
H	9.380664	3.117212	-7.639965
H	9.654975	0.209260	-7.102450
H	2.910237	6.338973	-6.091733
H	3.220047	4.610268	-6.275692
H	0.896986	4.304030	-7.222087
H	0.553752	5.997142	-6.973374
H	1.284752	4.126429	-4.652658
H	-0.359469	4.288369	-5.263070
H	-0.211568	6.802588	-4.829399
H	1.303854	6.504164	-3.966075
H	-0.750386	6.594767	-2.578207
H	-1.282802	5.225854	-3.225171
H	0.181441	5.167133	-2.353112
H	4.026071	9.896491	-1.373997
H	5.677087	9.476990	-1.854291
H	4.748880	7.678051	-3.399666
H	3.119018	8.109402	-2.905645
H	3.659283	7.426967	-0.541709
H	5.261657	6.939383	-1.049103
H	2.675790	5.563744	-1.525626
H	2.599994	3.659947	-2.451814
H	3.987721	2.952807	-3.282831
H	6.187469	5.456999	-2.130078
H	6.060777	3.768356	-2.547786
H	-1.206785	2.004649	-0.663702
H	-0.376525	2.788197	-2.012556
H	1.812295	1.859588	-0.880571
H	-0.187545	1.567181	1.199702
H	-8.167090	-4.692241	0.580639
H	-6.734012	-4.624054	1.648517
H	8.740321	-0.852591	-1.282625
H	6.015006	0.307294	2.117725
H	2.354730	0.182250	-1.722579
H	5.824465	-0.342548	-1.564020
H	6.535115	2.304461	-0.326518
H	7.016404	1.755520	-2.945901
H	10.469195	-0.331327	0.159005
H	9.258789	-0.860163	1.337830
Mg	2.976499	-0.137023	3.978676
Mg	-0.037408	-0.706168	4.081998
Mg	-2.982324	-2.583455	-1.019972
O	-3.588992	-3.215259	0.847670
O	4.776378	-0.256078	4.982446
H	5.145259	-1.140447	4.803684

H	5.518834	0.353940	4.900138
O	2.709310	1.983376	4.514209
H	2.061336	2.005951	3.784876
H	2.151132	1.780175	5.283624
O	-1.192704	1.119853	3.911505
H	-1.871752	1.333769	4.576703
H	-1.564510	1.082066	2.994524
O	-3.251495	-5.667124	-3.327545
O	-3.636722	-0.538076	-1.109673
H	-3.774247	-0.389503	-0.128420
H	-2.869417	-0.008225	-1.401151
O	-2.064122	-4.436311	-1.328865
H	-2.414996	-4.936046	-2.120996
H	-1.994286	-5.127611	-0.631309
H	3.828569	0.971205	-2.019819
H	1.188388	2.535704	1.586663
O	0.748428	-0.573953	0.764357
H	10.704660	-6.343667	1.257219
H	8.957409	-6.422154	1.546439
H	0.923051	-9.785449	-0.204757
H	-3.075837	-8.954134	-3.653111
H	-7.103721	-6.119887	0.739433
H	-11.620394	-2.151833	-5.157682
H	-11.853522	-1.006365	-0.617248
H	-2.306282	7.602746	7.328313
H	3.713110	-0.466959	9.521365
H	6.184450	-4.852066	6.323312
H	-3.129576	1.949309	9.153693
H	5.362329	-5.379413	-2.644363
O	-1.350407	-1.019171	5.675564
H	-0.722220	-1.256635	6.410857
H	-1.856903	-0.248302	6.006276
H	8.101836	-3.216976	-5.462936
H	9.989617	1.981093	-8.868669
H	7.860388	-0.832632	-6.146403
H	3.677823	-5.978185	2.751698
H	9.243812	1.757342	-2.857271
H	10.969521	2.340907	-7.422571
H	9.443249	1.791218	0.506540
H	2.014595	0.046636	9.464294
H	4.556131	10.153529	-3.043483
H	2.927842	5.621648	-7.705942
H	3.695469	-1.826815	-4.471040
H	1.688821	0.910569	-7.349300
H	1.209405	2.092715	-6.118176
H	-3.908449	7.322739	-3.384226
H	9.646552	-4.060146	-5.100983
H	-0.784450	-4.609278	7.563530
O	-2.102427	-2.144990	-2.893039
H	-2.741775	-2.197039	-3.614213
H	-1.762779	-1.196147	-2.867345

TS5

C	-10.964430	-0.937294	0.060343
C	-10.106952	0.269904	-0.271582
O	-10.587446	1.402830	-0.314416
N	-8.793187	-0.004169	-0.486282
C	-7.871906	1.038165	-0.893822
C	-7.446644	0.934399	-2.373393
O	-6.943292	1.916374	-2.924883
C	-6.702622	1.174104	0.108923
C	-7.231064	1.613359	1.506210
C	-6.767823	0.756336	2.704658
C	-5.365771	1.126765	3.197813
N	-4.740130	0.052936	4.052794
N	-7.741526	-0.231566	-3.033755
C	-7.439245	-0.372409	-4.449659
C	-8.511872	-1.108863	-5.295802
O	-8.285416	-1.311296	-6.487653
C	-6.059159	-1.036067	-4.715555
O	-6.016624	-2.362540	-4.196739
C	-4.896441	-0.209231	-4.173152
N	-9.675859	-1.500376	-4.697286
C	-10.595170	-2.471862	-5.330603
C	-10.258575	-3.893898	-4.837492
C	-8.869288	-4.399367	-5.270009
C	-8.413498	-5.660016	-4.501070
C	-7.615391	-5.359665	-3.224853
N	-6.178215	-5.098005	-3.529161
C	-4.009227	7.207386	-2.517772
C	-2.815571	7.908102	-1.888080
O	-1.697829	7.928237	-2.421444
C	-4.126904	5.711564	-2.151979
O	-2.990446	5.021131	-2.710106
C	-5.428645	5.113189	-2.677976
N	-3.037706	8.528796	-0.703340
C	-2.009609	9.343456	-0.054954
C	-1.088822	8.578840	0.909789
O	0.001956	9.051510	1.199759
C	-2.665511	10.484009	0.732394
N	-1.594660	7.430126	1.446191
C	-1.123395	6.994296	2.741702
C	-1.942438	7.686345	3.854259
O	-2.741957	8.577290	3.598309
N	-1.694403	7.226827	5.117159
C	-2.668222	7.396859	6.201802
C	-3.264088	6.027338	6.569810
C	-3.841906	5.369745	5.311923
O	-4.582286	5.995062	4.559330
N	-3.423093	4.101282	5.053741
C	3.717520	-4.897042	3.298529
C	2.302605	-4.339575	3.549476
C	2.156773	-2.854785	3.888548
O	0.995222	-2.434961	4.149819
O	3.187794	-2.099778	3.872074
C	9.738778	-5.983276	1.225113
C	9.669320	-4.451130	1.228371
C	10.768563	-3.858078	0.336043
C	8.291081	-3.982858	0.748645
C	5.860358	-3.682641	6.677407
C	6.673332	-2.732154	5.823275
C	6.672205	-2.845337	4.421674
C	7.403227	-1.680221	6.394700
C	7.358430	-1.927501	3.621023
C	8.089771	-0.758979	5.597129
C	8.066046	-0.870894	4.203353
C	-1.319953	-4.951472	6.920143
C	-1.646910	-4.361321	5.541105
C	-1.904482	-5.447649	4.485953
C	-2.621852	-4.962590	3.229747
N	-1.815286	-4.051107	2.374718
C	-4.011627	2.300069	8.503451
C	-3.852822	1.582646	7.124785
O	-2.726294	1.717072	6.511389
O	-4.843535	0.957631	6.686473
C	2.766177	-0.388941	9.039210
C	2.835648	-0.039202	7.549318
C	1.571394	-0.390606	6.768482
O	0.658090	-0.997040	7.334967
O	1.528825	0.001789	5.510034
C	8.820725	-3.651586	-4.597775
C	8.218143	-4.354006	-3.382747
O	8.927150	-4.882636	-2.525443
N	6.859696	-4.339409	-3.338919
C	6.092337	-4.767710	-2.179141
C	5.434904	-3.557829	-1.481206
C	4.477340	-3.929694	-0.334274
C	3.031949	-4.303008	-0.735634
C	2.095507	-3.091390	-0.834381
N	0.659491	-3.511193	-0.898150
C	0.146379	-8.961046	0.014066
C	0.728868	-7.691988	0.632854
C	-0.231928	-6.498723	0.680482
O	-1.436817	-6.656736	0.398019
O	0.298696	-5.377492	1.038567
C	-2.661888	-8.064040	-3.209373
C	-3.677234	-6.942117	-3.475611
O	-4.802698	-7.266351	-3.968593
C	2.080000	1.477153	-6.725215
C	3.092826	1.039378	-5.685199
O	3.759321	1.856716	-5.032781
N	3.229979	-0.292924	-5.529389
C	4.091218	-0.891347	-4.521276
C	5.522615	-1.122180	-5.011831
O	5.953793	-2.269281	-5.171154
N	6.267024	-0.021576	-5.250507
C	7.671272	-0.146178	-5.597418
C	8.163386	1.086001	-6.350067
O	7.628512	2.182595	-6.220993
N	9.266538	0.857363	-7.123218
C	9.974674	1.892567	-7.864714
C	2.685072	5.231997	-6.827096
C	1.182724	4.969633	-6.629047
C	0.722509	4.709307	-5.176052
C	0.539211	5.988572	-4.354936
N	-0.197263	5.757586	-3.073304
C	4.721078	9.377437	-2.466355
C	4.204549	7.956800	-2.734454
C	4.458242	6.974581	-1.576392
N	3.838356	5.676934	-1.807606
C	4.388245	4.607089	-2.406325
N	3.600159	3.565043	-2.687695
N	5.692105	4.535176	-2.737943
P	-0.755864	-1.059960	1.095024
O	-1.466829	0.433740	0.988081
O	-1.385404	-2.046059	0.106898
O	-0.992312	-1.432909	2.580318
P	-4.707619	-2.080931	1.594670
O	-5.174255	-2.408607	3.007007
O	-4.171758	-0.660588	1.428318
P	-6.332582	-2.949249	-0.724684
O	-7.660360	-2.500812	-1.315233
O	-5.116583	-2.914444	-1.664240
O	-6.123813	-2.109974	0.627556
O	-6.428143	-4.504829	-0.261318
C	-7.206358	-4.816746	0.899508
C	1.692385	0.805469	1.550292
C	8.565724	-0.204826	-0.542714
C	8.390558	1.131964	-1.248873
O	-1.178100	0.133701	-2.293927
C	-0.031376	0.617099	-2.068892
O	1.064609	0.147083	-2.462745
C	-0.046857	2.000309	-1.378784
C	1.084624	2.365550	-0.386458
C	1.113001	3.904265	-0.251664
O	0.868253	4.444115	0.823845
O	1.392210	4.517545	-1.376418
N	0.907474	1.784256	0.945903
O	1.470396	0.618906	2.808748
C	3.073209	0.488792	1.093342
N	3.970775	0.300220	2.151994
C	5.172959	0.154642	1.644082
N	5.120916	0.199378	0.284826
C	3.802134	0.407362	-0.099262
N	3.515593	0.421061	-1.437281
C	6.256216	0.066889	-0.630685
O	7.273326	-0.589742	0.067280
O	8.599920	0.897507	-2.721790
C	6.873466	1.409658	-1.113697
O	6.305822	1.819066	-2.372645
C	9.591015	-0.133626	0.597477
O	9.579139	1.130428	1.228079
H	-10.450193	-1.885704	-0.118813
H	-11.261085	-0.887823	1.112726
H	-5.992253	1.901770	-0.295238

H	-6.168772	0.226233	0.185119
H	-8.324392	1.582006	1.487381
H	-6.974334	2.665958	1.688846
H	-6.783171	-0.297017	2.416095
H	-7.478877	0.870240	3.532006
H	-5.397509	2.037864	3.805268
H	-4.707062	1.255953	2.342703
H	-3.751538	-0.022631	3.808849
H	-5.090539	-0.920990	3.791011
H	-4.810458	0.279030	5.080875
H	-8.455059	1.963760	-0.868931
H	-8.456508	-0.968077	-0.485042
H	-5.986911	-1.131319	-5.802462
H	-4.963513	0.830325	-4.503758
H	-4.868970	-0.215881	-3.079590
H	-3.949680	-0.629747	-4.530445
H	-5.709792	-2.354931	-3.260656
H	-7.387005	0.643117	-4.854380
H	-7.793590	-1.092248	-2.473616
H	-11.029477	-4.593682	-5.189402
H	-10.321685	-3.894604	-3.740051
H	-8.128150	-3.608840	-5.126929
H	-8.888520	-4.600727	-6.346959
H	-7.802995	-6.303940	-5.145288
H	-9.289616	-6.259647	-4.224084
H	-7.627517	-6.211588	-2.541510
H	-8.012023	-4.490694	-2.693020
H	-5.631514	-6.005168	-3.847248
H	-5.669939	-4.779265	-2.694106
H	-6.073797	-4.319404	-4.192181
H	-10.453322	-2.375357	-6.408643
H	-9.740209	-1.362588	-3.696939
H	-4.092368	5.617823	-1.055639
H	-6.292316	5.611390	-2.226754
H	-5.488318	5.232103	-3.764459
H	-5.522659	4.045169	-2.451454
H	-3.147922	4.075129	-2.578266
H	-4.941046	7.697178	-2.218607
H	-3.243770	10.085826	1.571723
H	-1.883814	11.122440	1.147324
H	-3.307472	11.084657	0.080754
H	-1.364484	9.735287	-0.843522
H	-3.983016	8.582788	-0.349674
H	-0.068183	7.266746	2.820139
H	-1.200948	5.905938	2.826120
H	-2.576227	7.263211	1.273938
H	-4.074464	6.162958	7.295043
H	-2.509101	5.386422	7.040467
H	-3.890746	3.647370	4.280455
H	-3.003680	3.470705	5.742127
H	-3.445878	8.055184	5.814061
H	-1.100940	6.409468	5.183363
H	4.249867	-4.287467	2.564755
H	4.310222	-4.915643	4.217929
H	1.806344	-4.891907	4.356980
H	1.672611	-4.517778	2.668900
H	9.823751	-4.097507	2.258802
H	10.603107	-4.150845	-0.706601
H	10.768898	-2.764232	0.383345
H	11.762226	-4.206470	0.642069
H	8.203924	-2.893363	0.742579
H	8.138207	-4.323311	-0.277928
H	7.485830	-4.385360	1.375565
H	9.584347	-6.362529	0.208337
H	6.034597	-3.509623	7.742341
H	4.789754	-3.552608	6.482011
H	6.112742	-3.645470	3.950405
H	7.418320	-1.571460	7.476000
H	7.327369	-2.026660	2.542154
H	8.644472	0.048536	6.066795
H	8.587215	-0.152591	3.575054
H	-2.156287	-5.547907	7.303080

H	-0.441174	-5.606234	6.871722
H	-0.826537	-3.713413	5.214277
H	-2.533025	-3.719531	5.622986
H	-2.539180	-6.230273	4.923413
H	-0.961002	-5.938524	4.209686
H	-3.536713	-4.417551	3.481538
H	-2.888826	-5.803490	2.585425
H	-2.480215	-3.661465	1.658157
H	-1.436612	-3.209987	2.840493
H	-1.030376	-4.536567	1.890222
H	-4.207179	3.363859	8.323152
H	-4.853795	1.875896	9.050736
H	3.677689	-0.543988	7.064285
H	3.030783	1.031382	7.406334
H	2.591161	-1.458813	9.175304
H	9.296941	-2.727705	-4.251883
H	4.897925	-2.961967	-2.232411
H	6.234442	-2.916576	-1.095193
H	4.433396	-3.095160	0.376344
H	4.915644	-4.766852	0.222365
H	2.611259	-4.967186	0.027239
H	3.030976	-4.869802	-1.677422
H	2.294385	-2.439450	-1.688802
H	2.164478	-2.468016	0.058327
H	-0.008689	-2.738683	-0.650688
H	0.354077	-3.872413	-1.802194
H	0.460739	-4.256769	-0.176951
H	6.772000	-5.288335	-1.503596
H	6.379024	-3.766678	-4.025884
H	-0.764789	-9.259928	0.538179
H	-0.126343	-8.796059	-1.032419
H	1.628530	-7.364641	0.097058
H	1.059021	-7.872931	1.664606
H	-1.787688	-7.919751	-3.853504
H	-2.317616	-7.994848	-2.171771
H	2.533018	2.242204	-7.357665
H	4.091946	-0.267717	-3.628326
H	2.638946	-0.907416	-6.067314
H	8.268941	-0.234956	-4.677550
H	5.875067	0.907049	-5.124797
H	9.443303	2.830294	-7.697010
H	9.621422	-0.086274	-7.165706
H	3.009472	6.145846	-6.316109
H	3.279279	4.403392	-6.426280
H	0.903296	4.095266	-7.225261
H	0.600091	5.803929	-7.041916
H	1.427323	4.036544	-4.673320
H	-0.244711	4.190539	-5.207712
H	-0.038367	6.725250	-4.920790
H	1.499300	6.441202	-4.092852
H	-0.517017	6.659523	-2.674216
H	-1.094101	5.268253	-3.208377
H	0.389233	5.235514	-2.345775
H	4.242988	9.809333	-1.581041
H	5.803941	9.383532	-2.299446
H	4.659662	7.549189	-3.645813
H	3.122554	7.990054	-2.914217
H	4.037432	7.372997	-0.647924
H	5.533843	6.858718	-1.396720
H	2.853709	5.541794	-1.534194
H	2.620898	3.642129	-2.395956
H	3.889629	2.865456	-3.365648
H	6.302940	5.254960	-2.385018
H	6.075506	3.572636	-2.726299
H	-1.019625	2.150870	-0.904198
H	0.022448	2.720031	-2.202721
H	2.040563	2.057121	-0.803971
H	-0.710565	1.041868	0.832373
H	-8.244893	-4.489445	0.770405
H	-6.779282	-4.339977	1.786479
H	8.850589	-0.996623	-1.251600
H	6.092531	0.006746	2.182982

H	2.523759	0.367363	-1.751512
H	5.893132	-0.504559	-1.498047
H	6.632150	2.182291	-0.370062
H	7.031747	1.524429	-2.983199
H	10.581675	-0.357386	0.162997
H	9.388240	-0.899039	1.354513
Mg	3.042161	-0.083951	4.030557
Mg	-0.066493	-0.620241	4.128040
Mg	-3.068298	-2.675482	-0.955849
O	-3.843414	-3.152511	0.885554
O	4.830754	-0.297611	5.036331
H	5.192168	-1.179409	4.834670
H	5.583320	0.305270	5.043820
O	3.048448	2.023598	4.647347
H	2.680996	2.469463	3.870234
H	2.270581	1.815482	5.199704
O	-1.242310	1.177690	3.741973
H	-1.916066	1.411130	4.404842
H	-1.660176	0.985395	2.874359
O	-3.346524	-5.753814	-3.200627
O	-3.545562	-0.591650	-1.063809
H	-3.743599	-0.410634	-0.097926
H	-2.717378	-0.120792	-1.278924
O	-2.224690	-4.561324	-1.182640
H	-2.563271	-5.078632	-1.970562
H	-2.111099	-5.227924	-0.468918
H	4.122950	1.023452	-1.981028
H	0.593321	2.483464	1.618166
O	0.733088	-0.853034	0.721934
H	10.713184	-6.342446	1.576138
H	8.966469	-6.419662	1.869190
H	0.865080	-9.786634	0.056765
H	-3.098498	-9.045550	-3.399733
H	-7.178478	-5.902282	1.011522
H	-11.624521	-2.200712	-5.077226
H	-11.877931	-0.892404	-0.538019
H	-2.198524	7.866071	7.072467
H	3.702183	-0.119638	9.538529
H	6.105086	-4.726275	6.454382
H	-3.088255	2.220694	9.082267
H	5.323558	-5.484709	-2.496908
O	-1.385749	-0.751553	5.730318
H	-0.772584	-0.974605	6.478002
H	-1.890384	0.039701	6.026939
H	8.078180	-3.402723	-5.355692
H	9.982896	1.671781	-8.937171
H	7.803269	-1.059713	-6.181721
H	3.649234	-5.919678	2.916012
H	9.262634	1.552184	-2.957146
H	11.006900	1.996394	-7.513369
H	9.471457	1.731540	0.446925
H	1.944264	0.139632	9.528851
H	4.510600	10.032734	-3.315878
H	2.920967	5.346364	-7.889046
H	3.685801	-1.865930	-4.252861
H	1.721056	0.655116	-7.348435
H	1.223703	1.920344	-6.209682
H	-3.908720	7.293526	-3.602979
H	9.602747	-4.289742	-5.016214
H	-1.104839	-4.160637	7.645660
O	-2.044201	-2.282173	-2.784465
H	-2.638358	-2.227477	-3.542787
H	-1.606059	-1.381935	-2.696943

E:ADP:SAICAR:P_i

C	-10.889318	-0.800124	-0.234660
C	-10.051869	0.451928	-0.446081
O	-10.564486	1.566427	-0.528335
N	-8.708742	0.230644	-0.493168
C	-7.777999	1.302954	-0.788759
C	-7.399124	1.395198	-2.279079

O	-6.913029	2.437625	-2.716963
C	-6.546616	1.234026	0.141905
C	-6.930269	1.590114	1.601083
C	-6.271625	0.733364	2.697369
C	-4.766016	0.949115	2.874532
N	-4.171127	-0.166087	3.687212
N	-7.670677	0.305328	-3.072342
C	-7.276065	0.330871	-4.472099
C	-8.303281	-0.252630	-5.467621
O	-8.010647	-0.315726	-6.661235
C	-5.908228	-0.356738	-4.719311
O	-5.998468	-1.753402	-4.432971
C	-4.771642	0.278299	-3.927135
N	-9.502994	-0.683604	-4.983577
C	-10.419428	-1.510645	-5.786921
C	-10.149575	-3.005491	-5.523272
C	-8.781914	-3.494263	-6.034599
C	-8.380155	-4.872897	-5.467870
C	-7.631201	-4.798835	-4.132118
N	-6.185616	-4.479199	-4.329112
C	-3.865606	7.601944	-1.430139
C	-2.744069	8.257035	-0.647486
O	-1.588800	8.404716	-1.071197
C	-3.828434	6.050866	-1.433588
O	-2.863556	5.622311	-2.387117
C	-5.200840	5.473904	-1.777154
N	-3.071265	8.695258	0.596054
C	-2.110462	9.397826	1.440871
C	-1.209142	8.473310	2.272917
O	-0.169485	8.904860	2.753407
C	-2.846223	10.330054	2.410678
N	-1.678530	7.206742	2.487701
C	-1.284820	6.525982	3.699434
C	-2.148260	7.005191	4.885368
O	-3.062071	7.806246	4.732873
N	-1.802469	6.458736	6.083093
C	-2.693218	6.474218	7.242636
C	-3.046957	5.037879	7.637754
C	-3.745103	4.350404	6.462030
O	-4.737255	4.846165	5.946936
N	-3.144484	3.214894	5.995420
C	3.717514	-5.278641	2.647726
C	2.230799	-4.968032	2.873946
C	1.887085	-3.564713	3.377026
O	0.722876	-3.397013	3.847006
O	2.764465	-2.645815	3.258646
C	9.832415	-6.046602	0.425480
C	9.709274	-4.559118	0.779817
C	10.773532	-3.738875	0.038410
C	8.307167	-4.049883	0.435681
C	5.797459	-4.596545	6.210011
C	6.403920	-3.390852	5.521218
C	6.582175	-3.364655	4.127743
C	6.786266	-2.256417	6.251236
C	7.118976	-2.245865	3.486416
C	7.329356	-1.134636	5.615271
C	7.498525	-1.120433	4.226592
C	-1.389304	-5.849218	6.124806
C	-0.823555	-6.109450	4.719842
C	-1.858107	-6.460465	3.633280
C	-2.691527	-5.315292	3.050588
N	-1.930486	-4.390199	2.153079
C	-4.093352	1.096981	8.727570
C	-3.648346	0.633698	7.316309
O	-2.382980	0.476042	7.134514
O	-4.526684	0.517499	6.433595
C	2.666160	-1.677956	8.980808
C	2.827374	-1.294363	7.503691
C	1.617095	-1.702355	6.674114
O	0.821708	-2.525367	7.168554
O	1.457422	-1.161015	5.507037
C	8.975580	-2.890130	-4.875196
C	8.338911	-3.727708	-3.769365
O	9.023651	-4.307136	-2.925335
N	6.978666	-3.771609	-3.791926
C	6.198220	-4.341849	-2.703171
C	5.629029	-3.241931	-1.781253
C	4.756520	-3.767600	-0.624032
C	3.249830	-3.951369	-0.934636
C	2.406442	-2.773889	-0.432819
N	0.945437	-2.979451	-0.664817
C	0.200665	-8.784999	-1.276081
C	0.712041	-7.477320	-0.660086
C	-0.383460	-6.517542	-0.168058
O	-1.578050	-6.880031	-0.258332
O	0.013617	-5.406136	0.337537
C	-2.542610	-7.399700	-4.380513
C	-3.556529	-6.269841	-4.567075
O	-4.641970	-6.512904	-5.150852
C	2.289849	2.535511	-6.334953
C	3.265980	1.767202	-5.466161
O	3.933260	2.313774	-4.572754
N	3.399714	0.455744	-5.742532
C	4.199335	-0.448778	-4.929348
C	5.692515	-0.479876	-5.273782
O	6.223183	-1.558686	-5.559298
N	6.375876	0.684615	-5.215854
C	7.816515	0.709423	-5.409599
C	8.279739	2.020305	-6.051074
O	7.610163	3.045501	-5.991544
N	9.513288	1.944194	-6.638480
C	10.206074	3.075153	-7.250792
C	2.905892	6.259864	-5.860712
C	1.419870	6.154818	-6.223236
C	0.494970	5.887294	-5.026726
C	0.361919	7.080683	-4.080843
N	-0.486853	6.734881	-2.917164
C	4.867290	9.693365	-0.889757
C	4.318275	8.394823	-1.512122
C	4.140273	7.238523	-0.507119
N	3.510953	6.055650	-1.094980
C	4.111885	5.045260	-1.765770
N	3.341173	4.252312	-2.498523
N	5.446618	4.864374	-1.725041
P	-0.750071	-0.866232	2.024333
O	-1.373462	0.657455	2.126022
O	-0.645746	-1.302441	0.579368
O	-1.660823	-1.670479	2.992862
P	-4.623657	-2.207685	1.144274
O	-5.003254	-2.425531	2.596231
O	-4.077731	-0.847396	0.740636
P	-6.510429	-2.932975	-1.091830
O	-7.740105	-2.233672	-1.631991
O	-5.297166	-2.976963	-2.053241
O	-6.134470	-2.305747	0.322509
O	-6.816923	-4.499507	-0.797543
C	-7.552775	-4.836197	0.383444
C	1.822229	1.593633	0.462585
C	8.720015	-0.215361	-0.107610
C	8.625827	1.156583	-0.753409
O	-0.311082	-1.183096	-2.553495
C	0.438604	-0.178735	-2.353287
O	1.664918	-0.125958	-2.656633
C	-0.248804	1.036332	-1.688184
C	0.622983	2.287987	-1.680645
C	-0.086294	3.649544	-1.612270
O	-1.314300	3.714296	-1.458043
O	0.699052	4.665850	-1.823863
N	1.752071	2.223975	-0.748091
O	0.962106	1.717291	1.356003
C	3.070366	0.832748	0.619950
N	3.563215	0.271497	1.802057
C	4.810497	-0.090032	1.547105
N	5.152993	0.162881	0.241713
C	4.052216	0.713837	-0.368922
N	4.010482	1.044944	-1.704108
C	6.455781	0.103932	-0.429881
O	7.348632	-0.625980	0.347611
O	8.843444	1.025435	-2.238940
C	7.133899	1.491971	-0.620557
O	6.648907	2.183778	-1.784273
C	9.614605	-0.181585	1.138491
O	9.573453	1.092609	1.744709
H	-10.360912	-1.708406	-0.538737
H	-11.149191	-0.886428	0.825664
H	-5.785530	1.919521	-0.242271
H	-6.107536	0.234537	0.097574
H	-8.012416	1.458441	1.711316
H	-6.737770	2.655700	1.785817
H	-6.440241	-0.320876	2.458610
H	-6.774308	0.923096	3.654376
H	-4.553615	1.897006	3.381697
H	-4.257216	0.921086	1.914391
H	-3.182091	-0.318942	3.454187
H	-4.600702	-1.101051	3.384274
H	-4.275726	-0.009882	4.712950
H	-8.319463	2.234871	-0.604115
H	-8.364361	-0.726358	-0.559824
H	-5.722712	-0.276685	-5.793880
H	-4.722801	1.355749	-4.102996
H	-4.900915	0.119994	-2.852941
H	-3.820070	-0.184063	-4.202682
H	-5.726541	-1.928499	-3.507580
H	-7.159588	1.386935	-4.733647
H	-7.758274	-0.609399	-2.617951
H	-10.947407	-3.606637	-5.981495
H	-10.219993	-3.175299	-4.439764
H	-8.012000	-2.761220	-5.781160
H	-8.808548	-3.532548	-7.129288
H	-7.754232	-5.417326	-6.185350
H	-9.276380	-5.489004	-5.323596
H	-7.664751	-5.750600	-3.597929
H	-8.047759	-4.028349	-3.477469
H	-5.618697	-5.271040	-4.780009
H	-5.736738	-4.277737	-3.420918
H	-6.069707	-3.591306	-4.840322
H	-10.239779	-1.252279	-6.832543
H	-9.630249	-0.648669	-3.980804
H	-3.524698	5.702208	-0.435233
H	-5.946337	5.744652	-1.021752
H	-5.537278	5.852605	-2.747922
H	-5.168365	4.383856	-1.844045
H	-2.358786	4.849478	-2.015809
H	-4.830789	7.930030	-1.030775
H	-3.450982	9.748745	3.113315
H	-2.111616	10.890040	2.991631
H	-3.478711	11.034524	1.861976
H	-1.446803	9.964770	0.784994
H	-4.035408	8.653607	0.894424
H	-0.231261	6.745587	3.887358
H	-1.400017	5.443655	3.580062
H	-2.634745	7.047554	2.202358
H	-3.741547	5.051477	8.484474
H	-2.150867	4.491532	7.956894
H	-3.743101	2.620174	5.434176
H	-2.517081	2.686128	6.590685
H	-3.593127	7.009746	6.937721
H	-1.088702	5.741947	6.068890
H	4.191021	-4.505927	2.039129
H	4.256974	-5.327653	3.598706
H	1.790975	-5.680102	3.578364
H	1.659053	-5.104682	1.944774
H	9.870871	-4.443915	1.862159
H	10.605506	-3.795755	-1.042375
H	10.737058	-2.685413	0.333808
H	11.782542	-4.110681	0.252962

H	8.189937	-2.986336	0.656355
H	8.137941	-4.180628	-0.635316
H	7.531669	-4.600348	0.982508
H	9.677491	-6.187059	-0.650375
H	6.071868	-4.628914	7.267745
H	4.702467	-4.569057	6.153126
H	6.300801	-4.230078	3.536730
H	6.658739	-2.251813	7.330615
H	7.239401	-2.244174	2.409091
H	7.629161	-0.274819	6.207766
H	7.930499	-0.259391	3.722528
H	-2.068560	-4.989604	6.128470
H	-1.939610	-6.718653	6.504854
H	-0.113047	-6.945112	4.780568
H	-0.247400	-5.236370	4.405444
H	-2.569333	-7.196180	4.032919
H	-1.357078	-6.957225	2.793095
H	-3.159437	-4.692022	3.816317
H	-3.492378	-5.726927	2.432466
H	-2.631276	-3.928588	1.509482
H	-1.490862	-3.612300	2.660715
H	-1.222372	-4.860063	1.543786
H	-4.038861	2.191297	8.761168
H	-5.127236	0.802233	8.913039
H	3.712840	-1.767443	7.060237
H	2.978234	-0.217467	7.373313
H	2.511781	-2.753820	9.081393
H	9.303840	-1.944188	-4.430208
H	5.051377	-2.528081	-2.386564
H	6.477410	-2.686830	-1.369718
H	4.857891	-3.081380	0.225277
H	5.175757	-4.718872	-0.277788
H	2.872213	-4.860867	-0.454853
H	3.095942	-4.083613	-2.014085
H	2.675346	-1.838108	-0.928425
H	2.547486	-2.644322	0.643920
H	0.359086	-2.306790	-0.072086
H	0.670197	-2.736480	-1.625949
H	0.632387	-3.947152	-0.421855
H	6.856565	-5.006567	-2.142201
H	6.506794	-3.160544	-4.451460
H	-0.442839	-9.315714	-0.570123
H	-0.396351	-8.586465	-2.170459
H	1.334071	-6.922170	-1.374610
H	1.368042	-7.678202	0.196905
H	-1.554186	-7.074369	-4.719162
H	-2.456493	-7.629926	-3.311915
H	2.828680	3.345096	-6.832758
H	4.060724	-0.202494	-3.875838
H	2.809921	0.048314	-6.451241
H	8.323086	0.599893	-4.437492
H	5.896359	1.548814	-4.978192
H	9.559453	3.946655	-7.141140
H	9.972264	1.045359	-6.648779
H	3.106917	7.108478	-5.197743
H	3.253203	5.356384	-5.347691
H	1.284532	5.342557	-6.946132
H	1.094760	7.068804	-6.738091
H	0.862366	5.029876	-4.450815
H	-0.505836	5.618864	-5.387357
H	-0.073335	7.942276	-4.597133
H	1.338972	7.384405	-3.692723
H	-0.706034	7.520274	-2.273406
H	-1.435999	6.379452	-3.143494
H	-0.036129	5.929789	-2.358534
H	4.200532	10.068852	-0.106757
H	5.854691	9.534657	-0.442647
H	4.976471	8.055020	-2.321178
H	3.341650	8.594636	-1.971081
H	3.505755	7.555194	0.326087
H	5.102916	6.963647	-0.060797
H	2.497636	6.026806	-1.128489

H	2.314127	4.321942	-2.359449
H	3.725167	3.490843	-3.054642
H	5.968362	5.405733	-1.053732
H	5.827595	3.920839	-1.886865
H	-0.539818	0.728471	-0.680575
H	-1.183611	1.242168	-2.216089
H	1.114536	2.306484	-2.663254
H	-0.738241	1.269837	1.699280
H	-8.560495	-4.405019	0.351326
H	-7.031802	-4.476837	1.275044
H	9.065437	-0.975332	-0.820114
H	5.492131	-0.559599	2.234305
H	3.205576	0.643540	-2.212616
H	6.280810	-0.357243	-1.415549
H	6.904702	2.092553	0.270118
H	7.382950	1.986074	-2.416442
H	10.639181	-0.441491	0.816979
H	9.304258	-0.933965	1.870987
Mg	2.409088	-0.621413	3.593482
Mg	-0.283153	-1.672597	4.511234
Mg	-3.384676	-3.333400	-1.419195
O	-3.799529	-3.375531	0.519240
O	4.263152	-0.918959	4.522689
H	4.458535	-1.854026	4.347672
H	5.107657	-0.485643	4.696554
O	1.769315	1.323144	4.043091
H	1.462990	1.569017	3.140232
H	0.945497	1.158536	4.548700
O	-0.859813	0.471264	4.913928
H	-1.505859	0.561979	5.672004
H	-1.270776	0.795990	4.090487
O	-3.254531	-5.136720	-4.055413
O	-2.880898	-1.354761	-1.493641
H	-3.198107	-0.973258	-0.621817
H	-1.931192	-1.155182	-1.620876
O	-3.425463	-5.371773	-1.387272
H	-3.280701	-5.606554	-2.324189
H	-2.768395	-5.883993	-0.844849
H	4.891271	1.213879	-2.174248
H	2.626582	2.159311	-1.252131
O	0.615724	-0.808201	2.785354
H	10.823481	-6.439869	0.679872
H	9.084996	-6.647529	0.956714
H	1.034367	-9.438602	-1.554701
H	-2.845325	-8.299095	-4.918878
H	-7.623710	-5.925076	0.406819
H	-11.449685	-1.248955	-5.526878
H	-11.818972	-0.699737	-0.798592
H	-2.226507	7.007315	8.078613
H	3.552214	-1.389565	9.556934
H	6.124865	-5.527795	5.738912
H	-3.427652	0.697930	9.495720
H	5.384833	-4.947770	-3.122440
O	-1.505516	-2.123111	6.101430
H	-0.772196	-2.395452	6.716274
H	-1.934702	-1.337835	6.504791
H	8.291048	-2.671412	-5.693301
H	10.388086	2.894359	-8.315234
H	8.086620	-0.154018	-6.021448
H	3.834073	-6.242875	2.143375
H	9.612365	1.582475	-2.385302
H	11.161120	3.269367	-6.751587
H	9.541668	1.671764	0.931606
H	1.794687	-1.181665	9.416153
H	4.965568	10.470340	-1.652358
H	3.519481	6.396600	-6.756380
H	3.829360	-1.461023	-5.082822
H	1.793519	1.914325	-7.083093
H	1.530142	2.986900	-5.691291
H	-3.806965	7.938731	-2.469509
H	9.863105	-3.414538	-5.236044
H	-0.580736	-5.619829	6.825704

O	-1.688422	-3.592764	-2.606808
H	-1.974198	-4.182650	-3.346075
H	-1.250127	-2.784722	-2.930176

E:ATP:CAIR:Asp’

C	-10.836098	-2.335820	-0.743061
C	-10.164057	-1.029150	-1.128881
O	-10.773583	-0.167542	-1.759293
N	-8.874169	-0.838102	-0.715296
C	-8.278544	0.491840	-0.821698
C	-7.961568	0.917819	-2.268296
O	-7.748269	2.100847	-2.514966
C	-7.071596	0.617877	0.135465
C	-7.530453	0.681834	1.613250
C	-6.519963	0.167436	2.657742
C	-5.310087	1.068442	2.900471
N	-4.267326	0.386924	3.729221
N	-7.976735	-0.060181	-3.229005
C	-7.556523	0.227697	-4.589110
C	-8.407807	-0.428678	-5.705787
O	-7.990625	-0.391445	-6.862428
C	-6.058865	-0.132809	-4.791368
O	-5.847489	-1.440129	-4.240371
C	-5.128835	0.901289	-4.164281
N	-9.587933	-1.022476	-5.372183
C	-10.306056	-1.904535	-6.318537
C	-9.804472	-3.356297	-6.155825
C	-8.327574	-3.567327	-6.544893
C	-7.750638	-4.925428	-6.091434
C	-7.122778	-4.915242	-4.688357
N	-5.740525	-4.385173	-4.698209
C	-4.931116	6.946763	-0.175660
C	-3.824341	7.542602	0.679892
O	-2.762507	7.986725	0.216843
C	-4.718454	5.452844	-0.533577
O	-3.769730	5.363262	-1.588474
C	-6.033838	4.801887	-0.960406
N	-4.058916	7.575265	2.015795
C	-3.155343	8.243856	2.951783
C	-2.155183	7.330365	3.680540
O	-1.184575	7.839978	4.231406
C	-3.968625	8.977215	4.026351
N	-2.454823	6.006515	3.733074
C	-1.992187	5.208499	4.849294
C	-2.907273	5.440158	6.069122
O	-3.883668	6.178648	6.013103
N	-2.541216	4.754020	7.202556
C	-3.586458	4.332120	8.144204
C	-3.976068	2.830926	7.918504
C	-3.703756	2.481558	6.454200
O	-4.414424	2.929910	5.554000
N	-2.510410	1.855712	6.233023
C	4.231296	-5.431643	1.518532
C	2.775621	-5.544104	1.979834
C	2.335770	-4.510387	3.022250
O	1.104341	-4.450178	3.305626
O	3.238333	-3.768671	3.534864
C	10.366479	-5.125776	-0.203922
C	10.076227	-3.627532	-0.059354
C	11.126543	-2.788565	-0.799351
C	8.677036	-3.293740	-0.587911
C	6.225182	-5.191312	5.186040
C	6.865359	-3.906228	4.700108
C	6.975996	-3.632029	3.325584
C	7.347215	-2.940735	5.597939
C	7.540009	-2.442495	2.867284
C	7.915298	-1.742938	5.140481
C	8.012223	-1.483720	3.772279
C	-0.747029	-7.288717	4.722158
C	-0.491459	-7.643360	3.254883
C	-1.671738	-7.316092	2.328298

C	-2.074591	-5.845445	2.298178
N	-0.992393	-4.998275	1.725376
C	-4.291850	-1.361581	8.548259
C	-3.926954	-0.976623	7.087049
O	-2.690490	-1.053982	6.796957
O	-4.849837	-0.595914	6.317807
C	2.764219	-3.270900	8.400524
C	2.753555	-2.929357	6.910889
C	1.377019	-2.853139	6.243782
O	0.401432	-3.437118	6.728659
O	1.345272	-2.153819	5.142080
C	9.112395	-1.009879	-5.305538
C	8.586286	-2.234168	-4.548694
O	9.246259	-3.268861	-4.449991
N	7.333141	-2.086660	-4.047755
C	6.549744	-3.184892	-3.505262
C	5.612331	-2.722477	-2.387445
C	4.497676	-3.760146	-2.123540
C	3.141955	-3.370911	-2.745920
C	2.543967	-2.243146	-1.920560
N	1.231555	-1.720038	-2.396700
C	1.165770	-8.530324	-3.070175
C	1.366030	-7.626637	-1.854606
C	0.247608	-6.610973	-1.610466
O	-0.728556	-6.588304	-2.397997
O	0.411484	-5.847998	-0.592811
C	-1.744145	-6.927683	-5.906689
C	-2.696444	-5.780489	-5.605880
O	-3.969445	-6.109345	-5.726789
C	1.790415	3.720350	-5.834652
C	2.861766	3.381092	-4.827079
O	3.580186	4.268326	-4.322544
N	3.017230	2.078070	-4.540310
C	4.016602	1.656847	-3.573402
C	5.444233	1.545019	-4.099547
O	6.059914	0.474644	-4.037527
N	6.009535	2.678567	-4.552030
C	7.415579	2.705434	-4.883493
C	7.793545	4.099053	-5.365392
O	7.067635	5.072175	-5.190886
N	9.007414	4.163206	-5.983019
C	9.571554	5.406121	-6.474983
C	1.935007	7.331935	-4.646828
C	0.488986	7.236658	-5.144930
C	-0.474374	6.609792	-4.127394
C	-0.750840	7.520875	-2.931958
N	-1.530016	6.806089	-1.893575
C	3.471427	9.965485	0.925356
C	3.058727	8.776361	0.038246
C	2.688248	7.500262	0.821468
N	2.316933	6.386118	-0.053279
C	3.175297	5.590232	-0.722843
N	2.690244	4.668154	-1.556489
N	4.506048	5.704139	-0.578307
P	-0.980188	1.112851	2.717770
O	-2.014633	1.953826	3.472161
O	-0.003508	1.771479	1.785113
O	-0.350652	0.016595	3.633092
P	-2.196513	-1.240921	1.128376
O	-1.984885	-1.273318	-0.381047
O	-1.459609	-2.269443	1.996678
P	-4.632725	-2.826302	0.806775
O	-6.055123	-2.424646	0.579293
O	-3.802772	-3.438265	-0.316207
O	-3.817886	-1.483449	1.365154
O	-4.451501	-3.856720	2.049680
C	-4.664274	-3.406697	3.396924
C	1.654234	-1.221044	1.304017
C	8.234030	0.990427	-0.387960
C	7.839926	2.463715	-0.356016
O	-1.259368	-0.378756	-3.798111
C	-0.364037	0.522384	-3.837907
O	0.825967	0.316921	-4.220066
C	-0.764382	1.907897	-3.342443
C	-0.045814	2.306253	-2.032467
C	-0.747208	3.582645	-1.520853
O	-1.959153	3.511511	-1.252524
O	-0.036881	4.666330	-1.472151
N	-0.030071	1.199728	-1.076874
O	1.427695	-1.797349	2.438442
C	2.971556	-0.594530	1.203419
N	3.958405	-0.917818	2.131537
C	5.060820	-0.328719	1.731211
N	4.849005	0.388447	0.577343
C	3.508684	0.253061	0.250815
N	2.933705	0.856999	-0.838658
C	5.926872	0.914369	-0.277630
O	7.085141	0.205626	0.078708
O	7.998799	2.989679	-1.756102
C	6.300085	2.410694	-0.125485
O	5.612186	3.215038	-1.104027
C	9.414986	0.677584	0.547813
O	9.439681	1.531592	1.671016
H	-10.130017	-3.118254	-0.450159
H	-11.507178	-2.142974	0.099898
H	-6.519692	1.520874	-0.139667
H	-6.410064	-0.243086	-0.006926
H	-8.428085	0.060971	1.717332
H	-7.846391	1.706250	1.852866
H	-6.179967	-0.813618	2.316962
H	-7.038717	0.019436	3.612878
H	-5.567255	1.987964	3.429963
H	-4.811768	1.330144	1.963438
H	-3.414632	1.009950	3.743611
H	-3.988912	-0.465986	3.239433
H	-4.544179	0.116626	4.715203
H	-9.041991	1.220587	-0.525612
H	-8.356394	-1.553073	-0.214833
H	-5.889033	-0.171904	-5.871950
H	-5.329247	1.893940	-4.575677
H	-5.270601	0.959209	-3.081571
H	-4.084032	0.647897	-4.367683
H	-5.055881	-1.427847	-3.668345
H	-7.665707	1.308236	-4.719468
H	-7.839174	-1.010807	-2.910361
H	-10.436961	-4.026465	-6.754703
H	-9.955702	-3.649175	-5.107070
H	-7.714403	-2.765665	-6.128470
H	-8.237082	-3.463885	-7.631844
H	-6.979189	-5.260885	-6.794145
H	-8.541563	-5.686503	-6.116862
H	-7.069280	-5.940416	-4.306370
H	-7.767394	-4.348019	-3.998326
H	-4.596663	-5.351013	-5.414006
H	-5.371981	-4.395163	-3.746567
H	-5.742307	-3.393788	-4.952167
H	-10.106088	-1.525664	-7.322798
H	-9.883171	-0.977159	-4.404367
H	-4.324679	4.931248	0.351146
H	-6.760061	4.789222	-0.140388
H	-6.472518	5.344490	-1.804056
H	-5.869387	3.772507	-1.286115
H	-3.149757	4.602125	-1.414603
H	-5.890901	7.053371	0.340595
H	-4.503514	8.255872	4.651998
H	-3.283846	9.527958	4.673200
H	-4.673253	9.677820	3.567638
H	-2.562240	8.948238	2.366519
H	-4.969768	7.298038	2.354388
H	-0.966178	5.500301	5.084438
H	-2.003508	4.150910	4.563254
H	-3.347837	5.726106	3.355048
H	-5.036134	2.685183	8.138686
H	-3.394136	2.181820	8.580574
H	-2.246150	1.739965	5.245832
H	-2.254655	1.073993	6.830625
H	-4.440531	4.980512	7.948866
H	-1.829570	4.047733	7.041906
H	4.462261	-4.406562	1.215818
H	4.919853	-5.696624	2.324361
H	2.586924	-6.537728	2.407944
H	2.092447	-5.470744	1.125037
H	10.115193	-3.371804	1.009702
H	11.076094	-2.984356	-1.877297
H	10.954745	-1.717901	-0.646317
H	12.141243	-3.019635	-0.455395
H	8.425732	-2.237956	-0.442572
H	8.634854	-3.496420	-1.662074
H	7.904991	-3.897145	-0.095092
H	10.328019	-5.423085	-1.258757
H	6.260486	-5.267276	6.276028
H	5.176957	-5.228161	4.868047
H	6.602988	-4.354288	2.608678
H	7.276474	-3.127289	6.666622
H	7.596691	-2.248754	1.801599
H	8.278333	-1.014422	5.859636
H	8.445438	-0.555217	3.406366
H	-0.789119	-6.206766	4.877853
H	-1.687940	-7.724090	5.079049
H	-0.275047	-8.714882	3.163788
H	0.407066	-7.119424	2.910902
H	-2.551930	-7.889323	2.645900
H	-1.440723	-7.639575	1.304950
H	-2.289589	-5.457584	3.296025
H	-2.966403	-5.682159	1.693145
H	-1.313778	-4.015914	1.614927
H	-0.137855	-4.938809	2.348632
H	-0.673383	-5.361915	0.809959
H	-3.802700	-0.662000	9.234671
H	-5.371540	-1.335472	8.707909
H	3.313236	-3.681572	6.336801
H	3.267037	-1.980436	6.738282
H	2.224446	-4.202691	8.587424
H	10.176373	-0.891845	-5.090438
H	5.172218	-1.763330	-2.685243
H	6.190648	-2.513512	-1.483396
H	4.353664	-3.900001	-1.046774
H	4.806460	-4.738104	-2.511077
H	2.458547	-4.228832	-2.736993
H	3.276876	-3.068161	-3.792563
H	3.210956	-1.383461	-1.867386
H	2.374687	-2.587933	-0.902563
H	0.848527	-1.138009	-1.629724
H	1.277531	-1.110828	-3.242595
H	0.543151	-2.470816	-2.565204
H	7.236945	-3.960428	-3.162319
H	6.868385	-1.189985	-4.144518
H	0.233562	-9.095318	-2.985217
H	1.104187	-7.939712	-3.988344
H	2.300887	-7.057121	-1.933282
H	1.467536	-8.214323	-0.933645
H	-0.941682	-6.570633	-6.556474
H	-1.291859	-7.232214	-4.955577
H	2.258705	4.238401	-6.675876
H	4.061123	2.358949	-2.738594
H	2.223623	1.436971	-4.675073
H	8.007097	2.464151	-3.987794
H	5.503317	3.562730	-4.516930
H	8.834940	6.188788	-6.290942
H	9.542427	3.312870	-6.076521
H	2.015378	7.943314	-3.740643
H	2.358618	6.347750	-4.425663
H	0.464766	6.635392	-6.061030
H	0.120398	8.231437	-5.430753
H	-0.059093	5.668969	-3.748689
H	-1.429201	6.366360	-4.610315

H	-1.299800	8.417917	-3.234708
H	0.183696	7.844174	-2.464685
H	-1.816795	7.389647	-1.081649
H	-2.440509	6.419912	-2.203367
H	-0.972314	5.950273	-1.566418
H	2.665923	10.247402	1.611307
H	4.353989	9.724729	1.528016
H	3.868242	8.535436	-0.661515
H	2.196307	9.056421	-0.579175
H	1.836753	7.685957	1.481670
H	3.513130	7.191952	1.476875
H	1.336358	6.172134	-0.192960
H	1.659441	4.602140	-1.658112
H	3.274206	4.381700	-2.340595
H	4.858228	6.315694	0.140513
H	5.049057	4.838109	-0.768422
H	-1.840280	1.926041	-3.159994
H	-0.531905	2.651554	-4.113591
H	0.993370	2.562426	-2.273974
H	-0.888686	0.651199	-1.107058
H	-5.530604	-2.742724	3.469029
H	-3.766648	-2.909937	3.773790
H	8.473102	0.659060	-1.411619
H	6.036904	-0.400470	2.177675
H	1.903579	0.787268	-0.852325
H	5.626013	0.719980	-1.317482
H	5.986257	2.754208	0.869693
H	6.351652	3.348512	-1.757842
H	10.340232	0.772386	-0.050027
H	9.362249	-0.359709	0.900000
Mg	2.902970	-1.783436	3.858281
Mg	-0.220044	-1.962172	3.650143
Mg	-2.247739	-2.964730	-1.585260
O	-2.083180	0.213419	1.696386
O	4.650116	-1.377176	4.946590
H	5.319997	-2.079153	5.023974
H	5.105308	-0.635856	4.524884
O	2.274873	0.242321	4.323168
H	1.382658	0.344420	3.891639
H	2.024112	0.186679	5.255841
O	-0.421333	-3.405703	-0.754138
H	-0.000353	-4.310654	-0.728225
H	-0.018183	-2.763535	-0.128049
O	-2.309037	-4.662751	-5.275564
O	-3.441102	-1.387218	-2.589219
H	-3.554673	-0.908715	-1.749461
H	-2.749065	-0.855727	-3.064989
O	-2.681187	-4.911129	-2.136969
H	-3.281508	-5.194385	-1.434500
H	-1.929881	-5.583140	-2.225079
H	3.239172	1.814946	-0.967886
H	0.028067	1.518615	-0.096726
O	0.872638	-1.257734	0.330677
H	11.360829	-5.379992	0.180430
H	9.630271	-5.727886	0.340674
H	1.994125	-9.239352	-3.170919
H	-2.262161	-7.772087	-6.361254
H	-4.854893	-4.296217	4.000374
H	-11.377681	-1.833119	-6.113573
H	-11.444032	-2.681524	-1.581126
H	-3.254972	4.489395	9.174881
H	3.791221	-3.377730	8.764187
H	6.730983	-6.066798	4.765230
H	-3.897887	-2.357810	8.768567
H	5.945998	-3.635849	-4.310269
O	-1.527164	-2.632124	5.052338
H	-0.943202	-3.048917	5.742633
H	-2.044760	-1.953245	5.587409
H	8.568901	-0.100402	-5.039713
H	9.774682	5.350210	-7.549976
H	7.643268	1.957937	-5.652135
H	4.420219	-6.099681	0.671971
H	8.528667	3.782661	-1.639932
H	10.500820	5.659783	-5.952335
H	9.126484	2.388544	1.274782
H	2.273442	-2.486747	8.985044
H	3.712613	10.835870	0.309461
H	2.574757	7.797925	-5.402892
H	3.729386	0.685236	-3.185471
H	1.261653	2.836919	-6.197660
H	1.073489	4.410020	-5.383286
H	-4.987788	7.514013	-1.108920
H	9.001472	-1.189991	-6.380070
H	0.058853	-7.665770	5.359285
O	-1.182291	-2.945579	-3.435963
H	-1.492733	-3.609450	-4.090999
H	-1.158421	-2.051885	-3.863455

INT-phospho-Glu193

C	-10.799786	-2.056239	-0.393997
C	-10.457674	-0.586317	-0.198503
O	-11.323834	0.282926	-0.113526
N	-9.124906	-0.347506	-0.090406
C	-8.588726	0.993353	-0.012693
C	-8.328329	1.635034	-1.392129
O	-8.188480	2.853399	-1.477297
C	-7.329373	0.998645	0.887246
C	-7.687222	0.584799	2.332890
C	-6.597040	-0.174306	3.110560
C	-5.408263	0.684242	3.535120
N	-4.362475	-0.157715	4.204427
N	-8.303347	0.792135	-2.482580
C	-7.935979	1.335179	-3.783452
C	-8.776399	0.844765	-4.993095
O	-8.433255	1.177565	-6.126346
C	-6.434573	1.107336	-4.103582
O	-6.166704	-0.292664	-4.166072
C	-5.487843	1.786289	-3.117271
N	-9.875137	0.064765	-4.764435
C	-10.512523	-0.724586	-5.843502
C	-9.882535	-2.136593	-5.873041
C	-8.384809	-2.162493	-6.247107
C	-7.680765	-3.499639	-5.901826
C	-6.922776	-3.495594	-4.565983
N	-5.565820	-2.875487	-4.676964
C	-4.732981	6.961848	1.401416
C	-3.442448	7.544569	1.937785
O	-2.615612	8.141859	1.231317
C	-4.540271	5.528651	0.830426
O	-3.613444	5.581341	-0.243336
C	-5.864395	4.943412	0.347103
N	-3.216094	7.355365	3.263523
C	-2.001599	7.817084	3.926460
C	-1.200992	6.692637	4.605767
O	0.026805	6.669464	4.549974
C	-2.307194	8.922461	4.947037
N	-1.942279	5.814791	5.319791
C	-1.399616	4.834579	6.229993
C	-2.490697	4.534592	7.266068
O	-3.637955	4.937334	7.096214
N	-2.094424	3.804525	8.342209
C	-3.058914	3.013210	9.112878
C	-2.712257	1.524722	9.027697
C	-2.530443	0.993909	7.595719
O	-3.065269	1.521241	6.622599
N	-1.706622	-0.087109	7.509837
C	4.303917	-5.599369	0.629963
C	2.816784	-5.635464	0.979611
C	2.403268	-4.800675	2.182123
O	1.178658	-4.802220	2.513299
O	3.307133	-4.119450	2.767620
C	10.340973	-5.029922	-1.424336
C	10.099984	-3.608316	-0.903384
C	11.316392	-2.711545	-1.164710
C	8.839134	-3.006723	-1.530544
C	6.462938	-5.977023	4.190934
C	7.112094	-4.648068	3.846529
C	7.045842	-4.132668	2.538865
C	7.767007	-3.875394	4.818030
C	7.594232	-2.888643	2.217723
C	8.317213	-2.628473	4.500838
C	8.229856	-2.121956	3.200983
C	-0.554586	-7.915068	3.716812
C	0.474124	-8.573830	2.798036
C	-0.129109	-9.084082	1.481289
C	-0.978343	-8.030542	0.761966
N	-0.292875	-6.755838	0.525555
C	-3.833326	-2.664354	8.617420
C	-3.721585	-2.237968	7.106311
O	-2.652614	-2.547088	6.469234
O	-4.669643	-1.544662	6.666346
C	3.178829	-4.579872	7.832624
C	3.116142	-3.923009	6.465591
C	1.757521	-4.026421	5.789815
O	0.987406	-4.949162	6.076378
O	1.512886	-3.104080	4.891683
C	8.943478	-0.161481	-5.602990
C	8.344760	-1.517563	-5.219770
O	8.751375	-2.568469	-5.711248
N	7.320475	-1.452949	-4.330815
C	6.430069	-2.581013	-4.063496
C	5.048486	-1.996699	-3.718795
C	3.916356	-3.003158	-3.490345
C	2.540736	-2.326325	-3.327766
C	2.385505	-1.585444	-1.999710
N	1.063935	-0.893925	-1.913678
C	0.989323	-7.882819	-4.254296
C	1.399092	-6.541525	-3.644590
C	0.250564	-5.808176	-2.971966
O	-0.916912	-5.963996	-3.272142
O	0.709298	-4.980871	-2.014914
C	-2.019066	-5.815256	-6.653444
C	-2.600873	-4.565170	-5.979461
O	-3.760100	-4.713508	-5.441317
C	1.678880	4.649601	-5.013936
C	2.842767	4.160956	-4.178906
O	3.641401	4.973791	-3.664108
N	2.971183	2.831887	-4.041018
C	3.984685	2.259866	-3.164427
C	5.424365	2.201934	-3.683254
O	6.072582	1.150014	-3.620017
N	5.960175	3.362019	-4.106858
C	7.369811	3.444023	-4.417760
C	7.729240	4.892049	-4.729019
O	7.012959	5.829434	-4.393918
N	8.915808	5.050368	-5.383924
C	9.448828	6.355493	-5.732686
C	1.932077	8.017478	-3.262123
C	0.621204	8.199302	-4.038274
C	-0.571841	7.480745	-3.387312
C	-1.086953	8.215477	-2.144934
N	-1.759026	7.279025	-1.206735
C	3.755944	9.693473	2.586795
C	3.236712	8.767645	1.474966
C	2.708166	7.406182	1.971179
N	2.372766	6.507999	0.865472
C	3.271299	5.828817	0.126496
N	2.848181	5.144171	-0.938427
N	4.578284	5.814105	0.437273
P	-0.280982	-3.887964	-1.096063
O	-0.765955	-2.865947	-2.123060
O	0.652916	-3.471550	-0.006107
O	-1.487957	-4.820815	-0.681633
P	-3.697531	-1.351611	0.705422
O	-3.616245	-1.710819	2.213858

O	-3.575591	0.135776	0.393405
P	-5.836990	-2.242961	-1.116216
O	-7.241031	-1.767297	-1.419107
O	-4.821858	-1.822774	-2.209785
O	-5.337106	-1.745557	0.329907
O	-5.913699	-3.850277	-0.999668
C	-5.005654	-4.601538	-0.171388
C	1.465788	-0.907758	1.622690
C	8.121671	1.060114	-0.302352
C	7.824996	2.522792	0.022832
O	-1.535141	1.069617	-4.090808
C	-0.549157	1.659010	-3.590739
O	0.674632	1.314846	-3.701455
C	-0.864867	2.914695	-2.735481
C	0.024805	3.053763	-1.482254
C	-0.440761	4.296566	-0.711366
O	-1.375572	4.183428	0.101899
O	0.131656	5.425735	-1.001268
N	-0.012168	1.875903	-0.610824
O	1.336455	-1.764110	2.620419
C	2.847757	-0.414928	1.462466
N	3.879179	-1.012136	2.187462
C	5.006020	-0.477640	1.783012
N	4.778895	0.469003	0.816297
C	3.403117	0.543847	0.615256
N	2.833735	1.392281	-0.286448
C	5.843791	1.062040	-0.013207
O	6.988426	0.270762	0.184099
O	7.962852	3.287824	-1.261375
C	6.292243	2.506504	0.315695
O	5.607206	3.459293	-0.522296
C	9.393188	0.546901	0.396743
O	9.630075	1.196689	1.626462
H	-9.951564	-2.634299	-0.770493
H	-11.117201	-2.484577	0.562670
H	-6.885149	1.997466	0.855683
H	-6.590257	0.307241	0.473339
H	-8.553605	-0.082294	2.281509
H	-8.015523	1.465496	2.902950
H	-6.231759	-1.001985	2.495408
H	-7.044070	-0.612653	4.012565
H	-5.708805	1.477427	4.226399
H	-4.922094	1.127342	2.660624
H	-3.604812	0.425325	4.570863
H	-4.000318	-0.813476	3.432670
H	-4.673292	-0.719028	5.037866
H	-9.362230	1.627025	0.431052
H	-8.457795	-1.079919	-0.352887
H	-6.277922	1.503163	-5.110530
H	-5.734039	2.844419	-2.996994
H	-5.542762	1.316361	-2.129250
H	-4.458169	1.692343	-3.477621
H	-5.741742	-0.595649	-3.336903
H	-8.101051	2.415037	-3.718060
H	-8.036931	-0.177655	-2.292135
H	-10.445300	-2.774810	-6.568604
H	-10.015442	-2.581478	-4.876406
H	-7.863276	-1.349770	-5.737629
H	-8.288984	-1.952669	-7.318046
H	-6.975467	-3.780534	-6.693365
H	-8.420259	-4.309073	-5.864991
H	-6.757173	-4.510040	-4.197351
H	-7.463223	-2.941989	-3.793337
H	-4.848893	-3.510494	-5.140288
H	-5.203492	-2.662132	-3.721392
H	-5.614804	-1.946154	-5.110251
H	-10.333478	-0.182497	-6.773807
H	-10.042411	-0.205960	-3.803455
H	-4.131567	4.896140	1.631353
H	-6.595793	4.867310	1.158324
H	-6.292498	5.563991	-0.446048
H	-5.705890	3.945192	-0.066102
H	-2.853346	4.982671	-0.052804
H	-5.502164	6.938306	2.180938
H	-2.981347	8.559470	5.730333
H	-1.381882	9.255906	5.424840
H	-2.776527	9.775635	4.450253
H	-1.366192	8.207192	3.132704
H	-3.960731	6.952328	3.813423
H	-0.479509	5.211041	6.689827
H	-1.158349	3.892704	5.718555
H	-2.943392	5.925722	5.444398
H	-3.518827	0.940625	9.488465
H	-1.806410	1.313073	9.610733
H	-1.869535	-0.752860	6.749071
H	-1.475441	-0.561536	8.371763
H	-4.036808	3.211611	8.672524
H	-1.144098	3.457896	8.322355
H	4.638284	-4.568475	0.490973
H	4.909614	-6.049228	1.420989
H	2.480484	-6.665500	1.144170
H	2.216373	-5.229765	0.157510
H	9.947872	-3.665282	0.184790
H	11.501277	-2.621369	-2.242199
H	11.159096	-1.703747	-0.767304
H	12.221897	-3.120239	-0.702084
H	8.617833	-2.009306	-1.137251
H	8.958402	-2.915066	-2.615429
H	7.965426	-3.640303	-1.339104
H	10.484331	-5.022249	-2.511520
H	6.570495	-6.207255	5.254048
H	5.394658	-5.954792	3.948092
H	6.544269	-4.706107	1.769896
H	7.828804	-4.246606	5.837654
H	7.509647	-2.504374	1.205768
H	8.812837	-2.049813	5.274952
H	8.645890	-1.148395	2.950768
H	-0.945395	-6.978381	3.313965
H	-1.404062	-8.582858	3.905071
H	0.957053	-9.412242	3.314614
H	1.270705	-7.850630	2.587699
H	-0.762106	-9.959519	1.680238
H	0.673814	-9.425566	0.813099
H	-1.879979	-7.813570	1.342124
H	-1.316821	-8.411235	-0.207708
H	-1.117398	-5.641368	-0.167758
H	0.039255	-6.303084	1.380664
H	0.527559	-6.884620	-0.060725
H	-3.565626	-1.800909	9.237955
H	-4.865696	-2.932777	8.849667
H	3.829776	-4.400158	5.782004
H	3.416063	-2.873713	6.516144
H	2.859664	-5.622915	7.773024
H	10.031701	-0.249890	-5.632826
H	4.758004	-1.323504	-4.535210
H	5.164291	-1.353686	-2.838162
H	4.125120	-3.627747	-2.610640
H	3.863409	-3.687875	-4.346399
H	1.749823	-3.078253	-3.390530
H	2.377506	-1.625121	-4.156926
H	3.155971	-0.825426	-1.857627
H	2.416541	-2.285382	-1.162111
H	0.892306	-0.569979	-0.944256
H	0.984906	-0.098226	-2.577840
H	0.288645	-1.578955	-2.102726
H	6.804311	-3.200859	-3.242104
H	6.998045	-0.538853	-4.028799
H	0.618240	-8.567116	-3.484763
H	0.191065	-7.745646	-4.986774
H	1.782553	-5.866518	-4.421646
H	2.209960	-6.653277	-2.918152
H	-1.189483	-5.552073	-7.312353
H	-1.651761	-6.468090	-5.854348
H	2.065672	5.298501	-5.803717
H	4.038828	2.820676	-2.228615
H	2.125524	2.250309	-4.164934
H	7.955861	3.109267	-3.549175
H	5.435314	4.232353	-4.009729
H	8.723164	7.097780	-5.398954
H	9.439905	4.225192	-5.633385
H	1.822542	8.284623	-2.203986
H	2.296353	6.988489	-3.310092
H	0.752371	7.814335	-5.055563
H	0.388610	9.267511	-4.148762
H	-0.259994	6.477138	-3.081320
H	-1.392265	7.363391	-4.104727
H	-1.779091	9.021186	-2.404475
H	-0.257761	8.657760	-1.586563
H	-2.117592	7.716332	-0.328604
H	-2.573582	6.787729	-1.587720
H	-1.048039	6.525768	-0.948805
H	2.972749	9.916531	3.319205
H	4.593055	9.235567	3.125383
H	4.038045	8.582091	0.749767
H	2.432886	9.264871	0.918474
H	1.811163	7.514397	2.588249
H	3.447890	6.918710	2.619789
H	1.412976	6.445821	0.536850
H	1.850616	5.237006	-1.184652
H	3.494642	4.952801	-1.702311
H	4.864054	6.219084	1.313946
H	5.108217	4.983457	0.101509
H	-1.916707	2.882423	-2.438101
H	-0.731017	3.799327	-3.371713
H	1.052243	3.214785	-1.829771
H	-0.555728	1.115399	-1.021802
H	-5.060721	-4.247889	0.861617
H	-3.982018	-4.516405	-0.529846
H	8.205126	0.892154	-1.389275
H	6.001493	-0.731910	2.102513
H	1.793072	1.471520	-0.272723
H	5.495324	1.023079	-1.055024
H	6.030518	2.735372	1.359152
H	6.340208	3.673291	-1.165395
H	10.232783	0.699343	-0.306281
H	9.319464	-0.531715	0.581455
Mg	3.035013	-2.340142	3.624282
Mg	0.075058	-3.230471	3.297331
Mg	-2.728802	-2.168329	-2.204934
O	-2.888992	-2.301182	-0.177668
O	4.923275	-2.441279	4.514832
H	5.501928	-3.112245	4.110736
H	5.424276	-1.616889	4.474738
O	2.574836	-0.635254	4.925068
H	2.105403	-0.064114	4.298389
H	1.868161	-1.155304	5.350770
O	-1.543890	-2.037600	3.687496
H	-2.012251	-2.174673	4.539008
H	-2.272826	-1.914640	2.986009
O	-1.909026	-3.523981	-5.949776
O	-2.183172	-0.144514	-1.782109
H	-2.739961	0.118940	-0.978187
H	-2.330370	0.462623	-2.528792
O	-3.224996	-4.145222	-2.831463
H	-3.420248	-4.375978	-3.771785
H	-2.535746	-4.773355	-2.556846
H	3.314338	2.274076	-0.410969
H	-0.524488	2.103916	0.238865
O	0.550849	-0.529535	0.890997
H	11.232513	-5.477348	-0.970419
H	9.487983	-5.682563	-1.207297
H	1.841921	-8.355305	-4.750680
H	-2.795454	-6.354737	-7.201815
H	-5.344035	-5.638550	-0.228762
H	-11.588729	-0.778123	-5.655852
H	-11.642585	-2.134037	-1.084983

H	-3.076505	3.337194	10.158657
H	4.195882	-4.544579	8.235517
H	6.911053	-6.796025	3.618009
H	-3.156566	-3.489586	8.849502
H	6.393151	-3.212799	-4.957046
O	-1.025692	-4.615616	4.436617
H	-0.344050	-4.924234	5.096565
H	-1.729518	-4.213853	4.973078
H	8.651048	0.634256	-4.913563
H	9.587023	6.450342	-6.815290
H	7.619456	2.792215	-5.263620
H	4.490883	-6.151292	-0.296986
H	8.521780	4.033053	-1.026013
H	10.407641	6.542534	-5.235987
H	9.303438	2.116513	1.434815
H	2.514917	-4.076544	8.542650
H	4.105577	10.640316	2.166501
H	2.719819	8.655038	-3.675318
H	3.684992	1.242000	-2.930456
H	1.103936	3.832446	-5.452821
H	1.020861	5.251237	-4.383321
H	-5.085316	7.609585	0.593914
H	8.600474	0.096850	-6.610411
H	-0.106094	-7.664487	4.683186
O	-2.590948	-1.637935	-4.211159
H	-2.321779	-2.324835	-4.892247
H	-2.115188	-0.805834	-4.396414

INT-cond

C	-10.688286	-1.373755	-0.505836
C	-10.196511	0.008251	-0.901304
O	-10.933525	0.833590	-1.438445
N	-8.892102	0.280087	-0.588335
C	-8.318689	1.547917	-1.006762
C	-8.010522	1.627607	-2.513798
O	-7.838966	2.726161	-3.034914
C	-7.073735	1.929622	-0.173975
C	-7.392068	2.649383	1.166862
C	-6.785359	2.014952	2.434654
C	-5.251844	2.008999	2.479377
N	-4.721953	0.991721	3.462943
N	-7.947684	0.448932	-3.222423
C	-7.457028	0.488847	-4.590301
C	-8.296390	-0.270496	-5.637852
O	-7.872398	-0.356291	-6.790608
C	-5.975408	0.028804	-4.661397
O	-5.849739	-1.232468	-4.001667
C	-5.027601	1.064956	-4.063314
N	-9.482316	-0.816283	-5.249038
C	-10.178674	-1.812999	-6.081771
C	-9.646267	-3.228633	-5.750481
C	-8.152897	-3.437190	-6.077692
C	-7.551549	-4.737981	-5.502480
C	-7.000698	-4.609288	-4.076726
N	-5.641423	-3.991314	-4.018484
C	-3.999702	7.343528	-1.291887
C	-2.716143	8.072488	-0.913717
O	-1.818785	8.315369	-1.726285
C	-3.890823	5.895061	-0.735805
O	-2.576583	5.436241	-1.046859
C	-4.950293	4.958224	-1.299289
N	-2.589607	8.380590	0.409147
C	-1.323745	8.825093	0.989885
C	-0.696161	7.682222	1.824960
O	0.264297	7.030634	1.402906
C	-1.502455	10.135374	1.760267
N	-1.313017	7.488233	3.010098
C	-1.031208	6.487305	4.020883
C	-1.945109	6.817919	5.219573
O	-2.841536	7.649431	5.090816
N	-1.698381	6.146226	6.379312
C	-2.802974	5.871363	7.326038
C	-3.186293	4.360192	7.258613
C	-2.890232	3.787445	5.865532
O	-3.445091	4.216440	4.859544
N	-1.835118	2.910993	5.818543
C	4.077440	-5.399850	2.230209
C	2.605132	-4.979741	2.363832
C	2.274079	-3.618882	2.993753
O	1.050836	-3.417646	3.277707
O	3.186040	-2.757615	3.178803
C	10.247521	-5.829652	0.028521
C	10.039945	-4.360903	0.420090
C	11.040970	-3.461212	-0.317183
C	8.604523	-3.931021	0.106230
C	6.120580	-4.797850	5.827447
C	6.725914	-3.572576	5.165498
C	6.687547	-3.411777	3.768857
C	7.306219	-2.546194	5.922356
C	7.188427	-2.261648	3.152950
C	7.829594	-1.401202	5.309952
C	7.771404	-1.247281	3.921047
C	-1.010349	-6.327597	5.657359
C	-0.178561	-6.477715	4.377467
C	-0.996416	-6.810100	3.112452
C	-1.762264	-5.636728	2.497756
N	-0.854573	-4.704701	1.773205
C	-3.992295	0.380701	8.560995
C	-3.659275	0.527821	7.048509
O	-2.590639	-0.057442	6.656517
O	-4.415610	1.239652	6.342474
C	2.870889	-2.133598	8.714853
C	2.892939	-1.696695	7.251382
C	1.618091	-2.053037	6.491238
O	0.810301	-2.837898	7.007279
O	1.453831	-1.486705	5.328506
C	9.253986	-2.466854	-5.155979
C	8.640313	-3.395607	-4.112592
O	9.344827	-4.101990	-3.389919
N	7.281714	-3.386367	-4.071428
C	6.531068	-4.124070	-3.063521
C	5.906137	-3.194241	-2.014703
C	5.097874	-3.977507	-0.964223
C	3.593882	-4.099473	-1.278444
C	2.873280	-2.843827	-0.797838
N	1.397885	-2.834184	-1.004318
C	0.710805	-8.860939	-1.862784
C	0.847763	-7.331210	-2.010349
C	-0.011833	-6.504437	-1.044243
O	-1.220376	-6.824660	-0.908246
O	0.543108	-5.515563	-0.445508
C	-2.078678	-7.444776	-4.911620
C	-2.589176	-6.045993	-4.543113
O	-3.818335	-5.807110	-4.714931
C	2.361908	2.751382	-6.395369
C	3.202541	2.367251	-5.184664
O	3.358430	3.144203	-4.233907
N	3.777492	1.140507	-5.236057
C	4.660567	0.625234	-4.199034
C	6.001112	0.217835	-4.811079
O	6.163635	-0.886791	-5.342434
N	6.934339	1.186620	-4.775693
C	8.282892	1.038460	-5.266109
C	8.794335	2.419053	-5.681160
O	8.450138	3.431791	-5.078120
N	9.656918	2.424089	-6.733450
C	10.252235	3.643613	-7.255139
C	2.828601	6.471610	-5.751811
C	1.323960	6.151056	-5.720259
C	0.785502	5.542813	-4.406645
C	0.670767	6.553700	-3.263763
N	-0.208688	6.066524	-2.156762
C	4.641948	9.751261	-0.622385
C	3.843124	8.507006	-1.061332
C	3.546672	7.507682	0.078731
N	2.972310	6.244067	-0.395331
C	3.685800	5.269566	-1.009876
N	3.098992	4.153216	-1.425440
N	5.000471	5.425432	-1.247943
P	-2.541505	-1.477189	1.716045
O	-2.380299	0.049694	1.966780
O	-2.480841	-1.936890	0.259038
O	-1.630024	-2.291790	2.639899
P	-5.476823	-1.476330	1.552084
O	-6.400662	-1.059573	2.662763
O	-5.230642	-0.564404	0.371236
P	-6.461355	-3.292775	-0.518803
O	-7.437695	-2.292585	-1.066380
O	-5.202474	-3.640685	-1.329494
O	-5.960802	-2.955091	0.995545
O	-7.147117	-4.740921	-0.259078
C	-8.310665	-4.775660	0.573683
C	1.089500	-0.156398	1.402888
C	8.144926	0.381636	-0.519327
C	8.007571	1.876867	-0.797102
O	-1.633690	-0.571530	-2.751777
C	-0.638698	-0.112172	-2.108605
O	0.561419	-0.413718	-2.329386
C	-1.036775	0.896488	-1.020683
C	0.071238	1.595182	-0.205575
C	-0.256658	3.105481	-0.178650
O	-1.246798	3.505619	0.455611
O	0.493193	3.836636	-0.937372
N	0.186102	1.023386	1.197366
O	1.013304	-0.361054	2.802658
C	2.583948	0.239210	1.098060
N	3.479633	0.202397	2.166310
C	4.695731	0.395215	1.696745
N	4.668421	0.540663	0.347389
C	3.330954	0.436911	-0.059710
N	3.028785	0.425983	-1.418241
C	5.860544	0.692448	-0.486725
O	6.885842	-0.093143	0.080286
O	8.167761	2.060466	-2.291345
C	6.484864	2.110721	-0.565150
O	5.929542	2.876872	-1.652287
C	9.303218	0.017790	0.428895
O	9.622213	1.015957	1.378693
H	-9.942304	-2.132698	-0.760971
H	-10.850527	-1.415973	0.576692
H	-6.470461	2.588358	-0.802184
H	-6.480969	1.027451	-0.000415
H	-8.478248	2.665816	1.313756
H	-7.087142	3.702667	1.093852
H	-7.127454	0.978827	2.490399
H	-7.171808	2.536594	3.319440
H	-4.825566	2.977174	2.753425
H	-4.846072	1.696865	1.515371
H	-3.803379	0.667835	3.105033
H	-5.351914	0.153307	3.424584
H	-4.616962	1.284706	4.460367
H	-9.093940	2.307873	-0.877897
H	-8.266707	-0.497756	-0.382775
H	-5.745265	-0.116496	-5.721593
H	-5.121256	2.016884	-4.593350
H	-5.257661	1.242319	-3.008981
H	-3.992311	0.717684	-4.128350
H	-5.252294	-1.151919	-3.217952
H	-7.495797	1.539821	-4.891157
H	-7.784504	-0.410514	-2.701971
H	-10.245188	-3.974980	-6.291142
H	-9.818649	-3.412501	-4.680652
H	-7.573111	-2.591016	-5.705188
H	-8.028106	-3.419353	-7.165653
H	-6.739942	-5.099782	-6.144722

H	-8.310220	-5.530850	-5.501138
H	-6.905074	-5.589274	-3.604100
H	-7.652709	-3.994127	-3.448501
H	-4.861505	-4.621584	-4.398282
H	-5.417220	-3.845799	-3.010147
H	-5.634786	-3.039673	-4.419254
H	-9.980666	-1.552345	-7.123481
H	-9.738743	-0.712314	-4.275458
H	-4.001470	5.943400	0.358791
H	-5.964198	5.291982	-1.056310
H	-4.863154	4.884753	-2.387184
H	-4.815039	3.957611	-0.880815
H	-2.264619	4.742583	-0.416339
H	-4.889607	7.835768	-0.887388
H	-2.259380	10.052952	2.547886
H	-0.563393	10.442267	2.229640
H	-1.827210	10.922642	1.074829
H	-0.652157	8.980028	0.145175
H	-3.309193	8.061296	1.043203
H	0.028564	6.484779	4.298303
H	-1.294997	5.480317	3.671446
H	-2.092952	8.077944	3.292723
H	-4.253280	4.229174	7.454298
H	-2.641850	3.797737	8.023254
H	-1.719881	2.401481	4.946876
H	-1.709057	2.287989	6.608009
H	-3.630288	6.504899	7.009386
H	-1.008405	5.405743	6.310016
H	4.660425	-4.627866	1.726869
H	4.524961	-5.579837	3.211911
H	2.058760	-5.732402	2.940000
H	2.125275	-4.987881	1.376401
H	10.209043	-4.260451	1.502472
H	10.866422	-3.513667	-1.397628
H	10.938950	-2.417078	-0.005016
H	12.073837	-3.771281	-0.118986
H	8.425084	-2.889150	0.384621
H	8.433454	-4.023654	-0.968468
H	7.870420	-4.552325	0.632133
H	10.084239	-5.955581	-1.048015
H	6.424160	-4.873782	6.874748
H	5.025511	-4.752162	5.797608
H	6.255383	-4.192240	3.155666
H	7.348292	-2.642230	7.004248
H	7.112103	-2.142575	2.077325
H	8.289363	-0.629965	5.921812
H	8.188857	-0.369025	3.433914
H	-1.713489	-5.492507	5.575425
H	-1.580425	-7.239360	5.872192
H	0.564756	-7.272378	4.524174
H	0.383703	-5.550870	4.225568
H	-1.722657	-7.597361	3.350738
H	-0.342490	-7.229138	2.337165
H	-2.276852	-5.040521	3.255740
H	-2.496168	-5.980279	1.769012
H	-1.348987	-3.822597	1.579723
H	-0.085863	-4.375574	2.390810
H	-0.435717	-5.095179	0.887906
H	-3.225610	0.896609	9.150328
H	-4.972795	0.804361	8.783072
H	3.734162	-2.151726	6.717880
H	3.054166	-0.616548	7.156509
H	2.711624	-3.211527	8.790759
H	9.865958	-1.721731	-4.638556
H	5.259468	-2.462657	-2.518271
H	6.700819	-2.615817	-1.535185
H	5.211579	-3.486534	0.008889
H	5.537034	-4.974915	-0.845275
H	3.154210	-4.971589	-0.785266
H	3.438306	-4.243054	-2.356000
H	3.274082	-1.950294	-1.279168
H	3.031878	-2.721390	0.277971
H	1.010114	-2.107095	-0.256967
H	1.121757	-2.431479	-1.905858
H	0.989934	-3.779601	-0.884400
H	7.228523	-4.820494	-2.595635
H	6.797385	-2.667293	-4.601437
H	1.093345	-9.199057	-0.893526
H	-0.341590	-9.149018	-1.927267
H	0.514624	-7.027453	-3.011349
H	1.892100	-7.022776	-1.903098
H	-1.167596	-7.362170	-5.511351
H	-1.814873	-7.964944	-3.984380
H	2.791316	3.649484	-6.845296
H	4.792219	1.418126	-3.462862
H	3.564798	0.528870	-6.010200
H	8.935019	0.659992	-4.467743
H	6.795010	2.006012	-4.201257
H	9.888657	4.467672	-6.640972
H	9.857942	1.548806	-7.192675
H	3.086064	7.272445	-5.048947
H	3.413650	5.590269	-5.469825
H	1.104669	5.438984	-6.522949
H	0.746286	7.053981	-5.959934
H	1.414833	4.700375	-4.099408
H	-0.217903	5.142790	-4.604886
H	0.228669	7.492150	-3.610992
H	1.644565	6.776169	-2.820948
H	-0.391518	6.820317	-1.486835
H	-1.161966	5.834390	-2.459893
H	0.115338	5.183870	-1.626329
H	4.111364	10.314069	0.153075
H	5.621664	9.471269	-0.219913
H	4.391585	7.986926	-1.855229
H	2.887276	8.812911	-1.502872
H	2.814959	7.923179	0.775118
H	4.457721	7.308602	0.661297
H	2.026043	6.022779	-0.103166
H	2.098293	3.942715	-1.238899
H	3.598765	3.583491	-2.101141
H	5.488969	6.184664	-0.803602
H	5.517277	4.540030	-1.419654
H	-1.717927	0.374457	-0.345998
H	-1.648405	1.654804	-1.519938
H	1.038633	1.462176	-0.674892
H	-0.765962	0.690006	1.497228
H	-9.122625	-4.186247	0.135808
H	-8.079754	-4.389379	1.571528
H	8.288861	-0.175591	-1.462175
H	5.616298	0.392065	2.256802
H	2.068523	0.110991	-1.645958
H	5.578729	0.349784	-1.491833
H	6.258299	2.635286	0.374483
H	6.711686	2.833308	-2.271700
H	10.175793	-0.208107	-0.208195
H	9.067250	-0.894537	0.985819
Mg	2.711000	-0.824557	3.845128
Mg	-0.191049	-1.702232	3.876046
Mg	-3.282246	-2.978688	-1.189796
O	-4.061910	-1.868284	2.265131
O	4.586796	-1.095929	4.701718
H	4.901223	-1.919958	4.288833
H	5.370563	-0.542987	4.802199
O	2.027032	1.110923	4.717926
H	1.394317	0.958479	3.971195
H	1.513528	0.790180	5.474582
O	-1.202912	0.152805	4.418250
H	-1.849892	0.095509	5.184534
H	-1.713367	0.214581	3.573329
O	-1.705716	-5.266283	-4.078816
O	-4.060476	-1.125240	-1.925917
H	-4.436443	-0.747322	-1.086311
H	-3.200273	-0.676486	-2.164772
O	-2.954404	-4.910799	-0.491572
H	-3.808453	-5.351373	-0.596577
H	-2.243741	-5.603719	-0.686236
H	3.227981	1.302097	-1.888467
H	0.445489	1.768017	1.846582
O	0.602255	-1.134083	0.678525
H	11.265109	-6.166203	0.258344
H	9.547481	-6.487612	0.556584
H	1.269014	-9.378415	-2.649165
H	-2.835972	-8.019101	-5.449284
H	-8.615387	-5.821592	0.645765
H	-11.253008	-1.744102	-5.890620
H	-11.632268	-1.569791	-1.014897
H	-2.510062	6.153657	8.340880
H	3.814690	-1.880614	9.209470
H	6.421962	-5.715661	5.313099
H	-3.959022	-0.674651	8.844343
H	5.747121	-4.713486	-3.556742
O	-1.271481	-2.565469	5.471615
H	-0.589117	-2.779555	6.163095
H	-1.890073	-1.938849	5.887853
H	8.500820	-1.962705	-5.763405
H	9.955289	3.812577	-8.295778
H	8.284940	0.324019	-6.091190
H	4.148676	-6.322692	1.647883
H	8.837103	2.744339	-2.377688
H	11.345291	3.607029	-7.198534
H	9.626107	1.845124	0.853179
H	2.054977	-1.648375	9.257905
H	4.805538	10.419578	-1.471704
H	3.136232	6.797885	-6.749874
H	4.214062	-0.238355	-3.701493
H	2.306627	1.963800	-7.150236
H	1.350521	2.991148	-6.059867
H	-4.064464	7.327048	-2.381940
H	9.920994	-3.052970	-5.793917
H	-0.363720	-6.115869	6.514457
O	-2.255962	-3.166923	-2.849306
H	-2.036915	-4.004024	-3.418970
H	-1.835372	-2.335918	-3.151239
